# Positive effects of tree diversity on tropical forest restoration in a field-scale experiment

**DOI:** 10.1126/sciadv.adf0938

**Published:** 2023-09-15

**Authors:** Ryan Veryard, Jinhui Wu, Michael J. O’Brien, Rosila Anthony, Sabine Both, David F.R.P. Burslem, Bin Chen, Elena Fernandez-Miranda Cagigal, H. Charles J. Godfray, Elia Godoong, Shunlin Liang, Philippe Saner, Bernhard Schmid, Yap Sau Wai, Jun Xie, Glen Reynolds, Andy Hector

**Affiliations:** ^1^ Department of Biology, University of Oxford, South Parks Road, Oxford OX1 3RB, UK.; ^2^ China Institute of Geo-Environment Monitoring, China Geological Survey, Beijing, China.; ^3^ Estación Experimental de Zonas Áridas, Consejo Superior de Investigaciones Científicas, Carretera de Sacramento s/n, E-04120 Almería, Spain.; ^4^ Sabah Forestry Department, 90000 Sandakan, Sabah, Malaysia.; ^5^ School of Environmental and Rural Science, University of New England, Armidale, NSW 2351 Australia.; ^6^ School of Biological Sciences, University of Aberdeen, Cruickshank Building, St Machar Drives, Aberdeen AB24 3UU, Scotland, UK.; ^7^ Division of Landscape Architecture, Faculty of Architecture, The University of Hong Kong, Hong Kong SAR, China.; ^8^ Dendra Systems, Unit A, Oakfield Industrial Estate, Stanton Harcourt Rd, Eynsham, Witney OX29 4TH, UK.; ^9^ Department of Biology, University of Oxford, 11a Mansfield Road, Oxford OX1 3SZ, UK.; ^10^ Faculty of Tropical Forestry, Universiti Malaysia Sabah, Jalan UMS, 88450 Kota Kinabalu, Sabah, Malaysia.; ^11^ Department of Geography, University of Hong Kong, Hong Kong, China.; ^12^ Rhino and Forest Fund e.V., Auf dem Stein 2, D-77694 Kehl, Germany.; ^13^ Department of Geography, Remote Sensing Laboratories, University of Zurich, Zürich, Switzerland.; ^14^ Conservation and Environmental Management Division, Yayasan Sabah Group, 88817 Kota Kinabalu, Sabah, Malaysia.; ^15^ Energy and Environment Institute, University of Hull, Hull, UK.; ^16^ The South East Asia Rainforest Research Partnership (SEARRP), Danum Valley Field Centre, Sabah, Malaysia.

## Abstract

Experiments under controlled conditions have established that ecosystem functioning is generally positively related to levels of biodiversity, but it is unclear how widespread these effects are in real-world settings and whether they can be harnessed for ecosystem restoration. We used remote-sensing data from the first decade of a long-term, field-scale tropical restoration experiment initiated in 2002 to test how the diversity of planted trees affected recovery of a 500-ha area of selectively logged forest measured using multiple sources of satellite data. Replanting using species-rich mixtures of tree seedlings with higher phylogenetic and functional diversity accelerated restoration of remotely sensed estimates of aboveground biomass, canopy cover, and leaf area index. Our results are consistent with a positive relationship between biodiversity and ecosystem functioning in the lowland dipterocarp rainforests of SE Asia and demonstrate that using diverse mixtures of species can enhance their initial recovery after logging.

## Teaser

Replanting with diverse mixtures of tree species enhances SE Asian tropical forest recovery.

## INTRODUCTION

A quarter century of ecological experimentation has demonstrated that when other factors are held constant, ecosystem functions like biomass production are generally positively related to levels of biodiversity (*1*–*4*). However, for practical reasons, the first generation of biodiversity manipulation experiments was conducted with systems that are relatively quick to respond, in particular, communities of grassland plants (*5*–*8*). More recent biodiversity experiments suggest that similar relationships between tree diversity and ecosystem functioning are present in many plantations and some forests (*9*), although there has been little research in tropical systems, in particular, outside of the Americas (*10*–*15*). It is also not clear as to what degree the results of biodiversity experiments extend to more natural settings (*16*) or whether they can be harnessed as a nature-based solution to forest restoration and carbon capture. Here, we report early results from a field-scale experiment that tests different approaches to the restoration of lowland tropical rainforests in Southeast (SE) Asia, focusing in particular on the role of the diversity of tree species used for replanting. Recent results from our lowland tropical forest study system in Sabah, Malaysian Borneo, show that active restoration, including enrichment tree planting, can accelerate recovery (*17*). Here, we go further in demonstrating that recovery (measured using remote-sensing estimates of aboveground biomass, canopy cover, and leaf area index) can be enhanced by replanting with ecologically diverse mixtures of tree species.

The Sabah Biodiversity Experiment (*18*–*20*) is designed to simultaneously test the applied question of whether increasing tree diversity in replanting schemes enhances restoration and the ecological hypothesis of whether there is a positive relationship between tree diversity and ecosystem functioning in tropical forests. There is ongoing debate over the generality of the positive relationship between diversity and ecosystem functioning demonstrated by experiments for real-world ecosystems (*16*), including the importance of tree diversity for the functioning of tropical forests with some predictions of no or small ecological differences among tree species in tropical forests. On the one hand, neutral theory and related ideas (*21*–*24*) hypothesize that tree species in tropical forests are ecologically identical (or near identical) and therefore predict a weak or absent link between diversity and functioning. In contrast, classical (Darwinian) niche theory predicts complementary differences among species coexisting within communities, leading to an ecological “division of labor” (*25*) and therefore, a positive, saturating relationship between diversity and function (*26*, *27*). The Sabah Biodiversity Experiment tests the hypothesis that increasing the diversity of tree species used to replant selectively logged forest enhances recovery rates. To address the underlying mechanisms, we tested the related hypothesis that enhanced recovery rates are associated with higher levels of functional (*28*), phylogenetic (*29*), and taxonomic (genus) diversity as predicted by niche theory (*26*, *27*). We also tested whether restoration rates were increased by the removal of the liana functional group, based on previous findings that lianas can reduce tree growth and survival (*30*, *31*).

To be relevant to forestry and forest restoration, the Sabah Biodiversity Experiment was designed to be field scale and covers 500 ha of selectively logged tropical forest in Malua forest reserve. The experimental treatments are applied to 4-ha plots and comprise different restoration approaches, including liana removal (“climber cutting”) and enrichment line planting where seedlings of the harvested native tree species are planted back into the resulting selectively logged vegetation (fig. S1). Approximately 100,000 seedlings of 16 different species of the dominant dipterocarp trees (table S1) have been planted along lines cut into the residual background vegetation left after selective logging in the 1980s, a setting unique among tree diversity experiments. The treatments include unplanted controls, single-species plots enrichment-planted with seedlings of one of 16 different species of dipterocarp, enriched plots planted with mixtures of 4 or 16 of these species, 16-species mixtures combined with liana removal, and manipulations of genus diversity (the number of genera planted) and (predicted) canopy structural complexity (Table 1). Enriched plots had equal density of saplings (planted every 3 m on parallel lines 10 m apart) with species equally represented in mixtures. Following standard practice in the study system, we used an initial round of enrichment planting in 2002–2003 followed by replacement of seedlings that died. The site has subsequently been monitored periodically for survival and growth of the planted seedlings. To gain an overview of the effects of the experimental treatments on the whole 500-ha area of the experiment over the initial stage of restoration, we used multiple sources of satellite remote-sensing data including RapidEye estimates of vegetation cover, aboveground biomass, and leaf area index in 2012 and longer-term estimates of changes in cover from Landsat from 1999 (before enrichment planting) to 2012 (*32*).

**Table 1.** Sabah Biodiversity Experiment treatments. Columns (left to right) indicate the number of species and genera of enrichment-planted trees, predicted canopy complexity, whether lianas are removed, and the number of replicate plots.

Number of species	Number of genera	Canopy complexity	Liana removal	Number of replicate plots
0	0	*	No	12
1	1	Low	No	32
4	2	Low	No	8
4	2	High	No	8
4	4	Low	No	8
4	4	High	No	8
16	5	High	No	32
16	5	High	Yes	16

*No experimental manipulation

## RESULTS

**Fig. 1.**
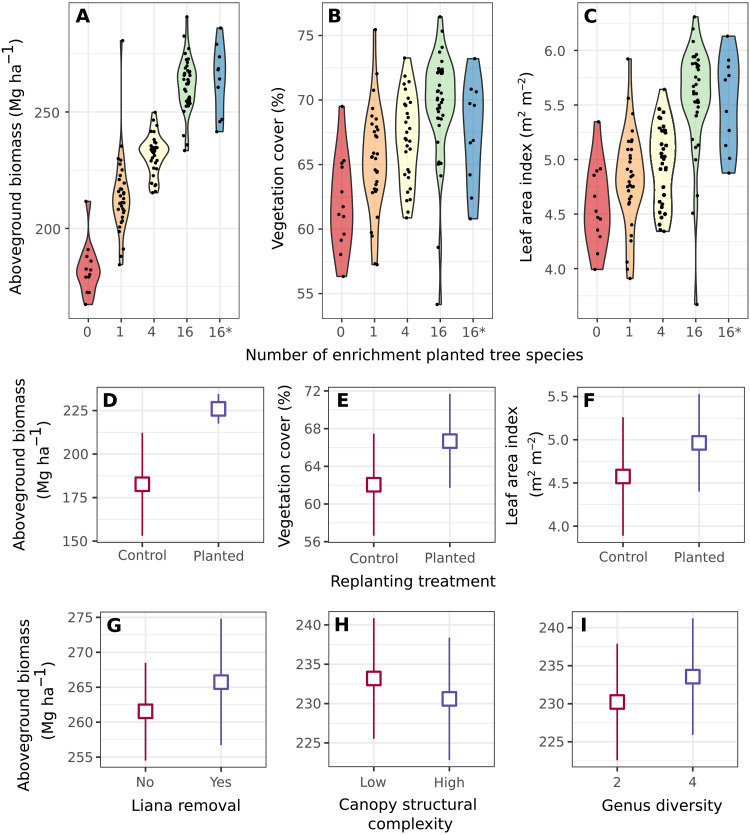
RapidEye satellite remote-sensing estimates as a function of restoration treatment a decade after initial planting. (**A** to **C**) Data points for individual plots overlaid on violin plots showing (left to right) aboveground biomass, percent vegetation cover, and leaf area index in relation to enrichment planting with seedlings of 0, 1, 4, or 16 species of dipterocarp tree species (16*: enrichment planting with 16 species plus liana cutting). (**D** to **F**) Treatment means (with 95% CIs) for unplanted controls versus enrichment-planted plots (panels as in top row). (**G** to **I**) Aboveground biomass as a function of (left to right) genus diversity of plots enrichment-planted with four-species (two genera versus four genera), canopy complexity (low versus high), and liana removal (climber cutting).

Analysis of estimates of vegetation cover, aboveground biomass, and leaf area index derived from RapidEye satellite data in 2012 revealed differences among the restoration treatments a decade after initial planting (Fig. 1 and table S2). Comparison of unplanted controls with enrichment-planted plots revealed that, after a decade, active restoration increased levels of estimated aboveground biomass {mean [95% confidence interval (CI)], 182.7 (153.1 to 212.3) Mg ha^−1^ versus 226.0 (217.5 to 234.6) Mg ha^−1^}, cover [62.1% (56.6 to 67.5) versus 66.7% (61.7 to 71.7)], and leaf area index [4.57 (3.89 to 5.26) m^2^ m^−2^ versus 4.96 (4.40 to 5.53) m^2^ m^−2^]. There were statistically significant differences for aboveground biomass [difference (95% CI), 43.3 (13.3 to 73.2) Mg ha^−1^] and cover [4.63% (1.08 to 8.07)] but not leaf area index [0.39 (−0.18 to 0.95) m^2^ m^−2^; Fig. 1 and table S3].

**Fig. 2.**
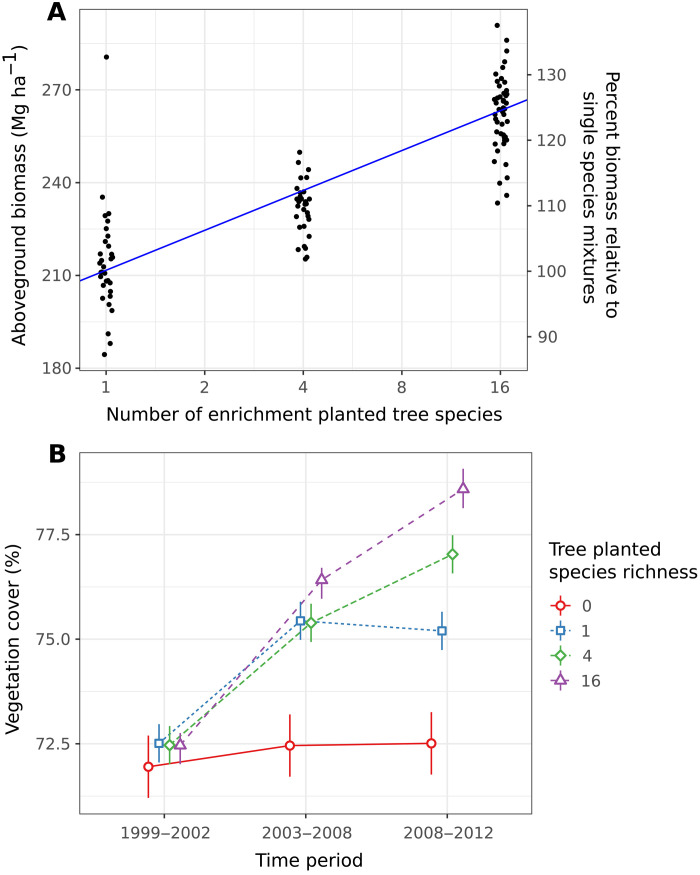
Effects of the diversity of enrichment-planted trees on aboveground biomass and vegetation cover. (**A**) Estimated aboveground biomass (RapidEye) as a function of the number of enrichment-planted tree species a decade after initial planting. The line is the regression slope with the log_2_ number of tree species from the mixed-effects model analysis (points jittered to reduce overlap). The second *y* axis re-expresses aboveground biomass values as percentages of the single-species treatment average. (**B**) Changes in vegetation cover over time as a function of the number of enrichment-planted tree species. Estimates of mean cover (with 95% CIs) for the Landsat monitoring periods 1999–2002 (before planting), 2003–2008, and 2008–2012 for plots enrichment-planted with seedlings of 0, 1, 4, or 16 species. Individual species richness mean values are jittered to avoid overlap.

While enrichment planting had a general positive effect on restoration, its effectiveness was positively related to the diversity of species used. The relationship was positive and approximately linear with the logarithm of the number of enrichment-planted species: Each doubling in tree species richness increased estimated aboveground biomass by 12.9 Mg ha^−1^ (10.3 to 15.1) (Fig. 2A), cover by 1.06% (0.44 to 1.66) (fig. S2), and leaf area index by 0.23 m^2^ m^−2^ (0.16 to 0.30) (fig. S3 and table S4).

These treatment differences in the RapidEye satellite data from 2012 were supported by estimates of changes in vegetation cover across three Landsat monitoring periods covering the preceding decade, which show the absence of treatment differences before restoration (1999–2002), the emergence of positive effects of enrichment planting (2003–2008), and the subsequent divergence of treatments (2008–2012) with those planted with a greater diversity of tree species showing stronger recovery of vegetation cover (Fig. 2B and table S5).

Our experimental design also contains a factorial manipulation of two other aspects of diversity within the four-species treatment level. Half of the four-species plots were enrichment-planted with seedlings of four species from four different genera and half with four species from only two genera. This manipulation of genus diversity is crossed orthogonally with a canopy structural complexity treatment that compares mixtures of four species with a lower or higher diversity of predicted mature tree height that is intended to produce canopies that are thinner and simpler or thicker and more complex (table S6). During the initial phase of the experiment, both manipulations produced only slight changes in estimated mean aboveground biomass with enhanced genus diversity and canopy structural complexity (Fig. 1 and table S7) that were statistically indistinguishable between treatments (vegetation cover and leaf area index showed qualitatively similar results; fig. S4 and table S7).

A subset of the plots planted with 16 species were also subjected to an additional treatment that reduced lianas in the tree canopy by stem cutting (climber cutting), another management practice often used in these forests (*18*). At the time of the RapidEye data snapshot in 2012, the liana removal treatment had only been applied to the southern block and the treatment had no clear effects on the satellite remote-sensing estimates of aboveground biomass (Fig. 1), cover, or leaf area index (fig. S4 and table S8). Previous analysis of longer-term field data extending to 2017 (*18*) has demonstrated positive effects of liana removal on the growth and survival of trees, in particular seedlings and saplings in the understory, most likely due to increased light availability [although with potential increased seedling mortality if cutting is followed by drought (*18*)]. A more complete test of the liana removal treatment will require a longer time series of more detailed field and remote-sensing data that can distinguish lianas from dipterocarp tree canopies when monitoring changes in tree canopy cover.

**Fig. 3.**
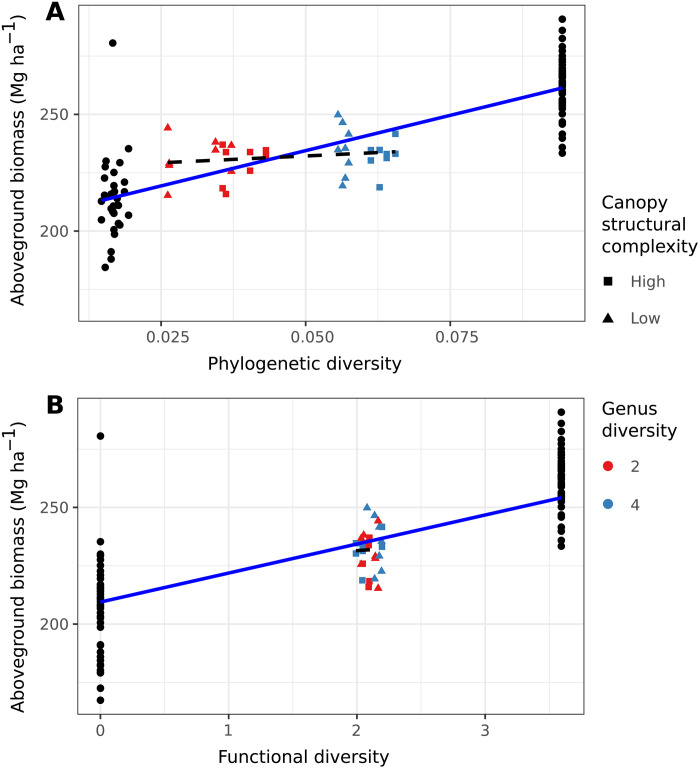
Estimated aboveground biomass as a function of PD and FD. Measures of (**A**) PD and (**B**) FD increase across the full diversity gradient from 1 to 16 species but not in relation to the treatments applied to the subset of four-species plots that manipulate genus diversity (two versus four genera: red versus blue points) and canopy complexity (lower versus higher: squares versus triangles). Solid blue lines show the positive relationship between estimated aboveground biomass and PD and FD across the full gradient from 1 to 16 species and dashed lines show the weaker, nonsignificant relationships for the subset of plots enrichment-planted with only four species.

To understand why the manipulation of diversity from 1 to 16 species had detectible impacts on multiple measures of restoration, while increasing genus diversity of the four-species mixtures from two to four genera did not, we calculated quantitative estimates of functional and phylogenetic diversity (FD and PD) for our species mixtures. Levels of aboveground biomass were positively related to levels of FD and PD across the full species richness gradient from 1 to 16 enrichment-planted species but showed only small, statistically indistinguishable increases from the two to four genera treatments and in relation to the manipulation of canopy structural complexity (Fig. 3 and table S9). The explanation for the lack of effect of our manipulation of genus diversity probably involves both the small increase in diversity from two to four genera relative to the increase across the whole gradient from 1 to 16 species and the fact that dipterocarp taxonomy when the experiment was designed did not accurately reflect the underlying evolutionary relationships (the genus *Shorea* is now thought to be polyphyletic, although dipterocarp taxonomy remains in flux). The analyses of FD and PD support this interpretation showing much smaller increases in diversity within the subset of treatments applied to the four-species mixtures than across the entire gradient from 1 to 16 species (Fig. 3). These results suggest that the benefits of low levels of diversification in enrichment planting can be increased by the use of seedling mixtures that are more species rich.

## DISCUSSION

Our results suggest that the positive relationship between biodiversity and ecosystem functioning observed in experiments in other ecosystems, including some forests, also applies to the lowland tropical rainforests of SE Asia. While our remote-sensing data have limitations (see Materials and Methods), the results reported here appear robust since the same qualitative patterns are evident in two different sources of satellite data one of which documents their progressive development over the first decade of the experiment. Comparing these satellite data with field data for a similar period (*19*) suggests that during the first decade of the experiment, the effects of diversity do not come simply through increased survival or higher trunk-diameter growth rates. Instead, we hypothesize that the differences detected by satellite remote-sensing are due to the development of different canopy architectures in monospecific and multispecies mixtures that are not captured by our standard measures of tree size (trunk diameter at base and breast height). Although there is no effect of our limited manipulation of canopy structural complexity at this stage of our experiment, diversity-dependent growth forms have previously been shown to play a role in generating biodiversity effects in the Wageningen biodiversity experiment (*8*), and evidence for complementarity among tree crowns has been observed for temperate-boreal species in another tree diversity experiment (*33*, *34*) and in a European network of permanent forest plots (*35*). Testing this hypothesis, and whether differences in canopy responses subsequently feed back to improve survival and diameter and breast height growth in mixtures, will require continued long-term monitoring, ideally including a coordinated combination of field and remote-sensing data providing more detailed measurements of individual tree architecture and canopy structure.

Because biodiversity was manipulated through the enrichment planting of tree seedlings into selectively logged forest vegetation, the Sabah Biodiversity Experiment provides a unique combination of experimental control within a real-world context. Although consistent with the results of the majority of other biodiversity experiments (*36*), the positive effects of biodiversity reported here (on estimates of forest biomass, cover, and leaf area index) contrast with some studies that have failed to find similar relationships in nonexperimental settings (*16*). Positive relationships between biodiversity and ecosystem processes like productivity are predicted when low levels of diversity are associated with vacant or underutilized niches (*37*), as is likely in the selectively logged forests of SE Asia where species of the dominant family of dipterocarp trees have been deliberately harvested. The satellite remote-sensing data presented here have limited ability to examine the detailed underlying biological mechanisms. However, this niche packing (niche complementarity) mechanism is supported by the positive effects of FD and PD seen in our results. In contrast, studies of real-world systems where diversity has not been reduced and where niches are occupied would not necessarily be expected to reveal a positive relationship between biodiversity and ecosystem functioning.

Recent analyses of secondary succession after deforestation provide a mixed picture of recovery rates. Some properties of forests at sites in the Americas and West Africa can recover old growth levels in as little as two decades so long as land-use intensity after deforestation is low (*38*), while other forests show longer-term reductions in biodiversity and carbon (*39*, *40*). Our results demonstrate the potential for the recovery of lowland forests in aseasonal SE Asia to be accelerated by active restoration through enrichment planting, especially with diverse mixtures of native tree species with complementary niches. Differences between our results from the forests of SE Asia and those from some other parts of the tropics may be due to characteristics of the dominant dipterocarp species that have the potential to slow the recovery of these forests, including the absence of a soil seedbank, intermittent mast fruiting, and the low dispersal ability of many species (*41*–*43*).

These initial results from our project suggest that the positive relationship between biodiversity and ecosystem functioning observed in many other experiments (*36*) is also found in the lowland tropical rainforests of SE Asia. This emphasizes the need to conserve the diversity of tree species in these forests to maintain the ecosystem functions and services that they provide, a matter of urgency given the recent estimate that 70% of Bornean dipterocarp species are threatened with extinction (*44*). Our results also suggest that replanting of these secondary forests with diverse mixtures of the native species previously removed by selective logging (*20*) may provide a nature-based solution for their accelerated restoration.

## MATERIALS AND METHODS

### Study system

The Sabah Biodiversity Experiment (http://sabahbiodiversityexperiment.org) occupies 500 ha in the southern part of the Malua Forest Reserve, in Sabah, Malaysian Borneo (fig. S1). The Malua Forest Reserve is an area of approximately 35,000 ha of predominantly selectively logged forest that is publicly owned through Yayasan Sabah (The Sabah Foundation), which holds a 100-year concession under its goal to increase socioeconomic standards in the state. Within the wider Yayasan Sabah logging concession is the Innoprise-FACE Foundation Rainforest Rehabilitation project (INFAPRO), a 25,000-ha area dedicated to promoting the rehabilitation of forests through large-scale enrichment planting within logged areas. To help provide practical recommendations, the Sabah Biodiversity Experiment followed INFAPRO enrichment planting techniques. The region experiences an average temperature of 27°C and an annual rainfall of >3000 mm, distributed between two wet seasons (*45*–*47*). The Malua Forest Reserve area has been logged twice, once in the 1980s and again in 2007. The 500-ha area of the Sabah Biodiversity Experiment itself was spared the second round of selective logging in 2007 due to the establishment of the experiment in 2002 and has therefore been recovering from the initial round of logging for nearly 40 years. Elevation at this site is under 250 m, with 0° to 20° range in topography. The prelogging timber volume of this region has been estimated at 193 to 221 m^3^ ha^−1^, of which dipterocarps account for the vast majority at 180 to 216 m^3^ ha^−1^ (*20*).

### Study species

The 16 species used in this experiment are native species belonging to the Dipterocarpaceae: *Dipterocarpus conformis* Slooten, *Dryobalanops lanceolata* Burck, *Hopea ferruginea* Parij, *H. sangal* Korth., *Parashorea malaanonan* (Blanco) Merr., *P. tomentella* (Blanco) Merr., *Shorea argentifolia* Sym., *S. beccariana* Bruck, *S. faguetiana* Heim., *S. gibbosa* Brandis., *S. johorensis* Foxw., *S. leprosula* Miq., *S. macrophylla* Ashton, *S. macroptera* King, *S. ovalis* Korth., and *S. parvifolia* Dyer. In general, dipterocarps in Sabah are emergent tree species, rarely found more than 1200 m above sea level (*48*). They have an array of characteristics which likely contribute to their dominance in SE Asian forests, including their symbiotic ectomycorrhizal associations (*49*) and wind-dispersed winged fruits (*50*). Reproduction takes place largely through “mast fruiting” events that occur between 2 to 10 years apart, where many or most of the dipterocarp species simultaneously produce fruit. Dipterocarps have recalcitrant seeds (*51*) and no soil seed bank (*17*). Instead, successful recruits form a seedling bank, which often suffers from heavy herbivory (*52*). Dipterocarps dominate the lowland forests of SE Asia in terms of biomass but have been heavily selectively logged (*53*).

### Experimental design

The Sabah Biodiversity Experiment features several experimental treatments within its replicated, randomized block design. The experiment consists of 124 4-ha (200 m by 200 m) plots, divided into two blocks separated by an old logging road (60 plots in the north block and 64 to the south). Each plot (apart from the unplanted controls) is enrichment-planted with a mixture of seedlings with a controlled species number (richness) and composition. The design ensures at least one replicate plot for each species richness and composition treatment level in each of the two blocks. Each plot contains 20 parallel planting lines, separated by 10-m areas of remnant vegetation left after the prior selective logging. Within each line, seedlings were planted with 3-m spacing, and planting lines were initially cleared of bamboo, lianas, and shrubs up to a maximum of 1 m either side of the line of planted seedlings. The experiment was primarily designed to manipulate the diversity and composition of enrichment-planted dipterocarps but also investigates the forest management practice of liana removal (climber cutting). One hundred fourteen of the plots make up a gradient in the diversity of enrichment-planted tree species comprising mixtures of 1, 4, or 16 species. The remaining 12 plots were left as naturally regenerating unplanted controls (six in each block). The design uses a set of 16 species that were available in the local seedling nursery in sufficient numbers. These 16 species were grown in single-species enrichment planting “monocultures” and combined together to form enrichment planting “polycultures” of 4 or 16 species (Table 1). The plots enrichment-planted with only a single species of dipterocarp allow a comparison of individual species identity effects since each species has a replicate in each of the two blocks (a total of 32 one-species plots).

The intermediate four-species diversity level is composed of 16 different species compositions that produce two further treatments that are factorially crossed. These two treatments manipulate genus diversity (two levels) and predicted canopy structural complexity (two levels). The genus diversity treatment compares mixtures of four species comprising two or four dipterocarp genera. The canopy structural complexity treatment also features two levels that either combine species with similar predicted mature heights or with a wider range of these predicted values. In total, this factorial manipulation of genus and canopy structural complexity comprises 32 plots (the 2^2^ factorial combination of the four treatments, each with four replicate species compositions, each replicated in the two blocks) (table S6).

Sixteen plots of the most diverse (16 species) mixtures underwent two rounds of liana removal (climber cutting), which were compared with 32 plots enrichment-planted with the same number of species but without this local climber cutting restoration strategy (*18*). Because of practical constraints, these cuttings took place in two stages. In July 2011, 10 plots were cut in the southern block, and in June 2014, these 10 plots, as well as six plots in the northern block, underwent a full round of liana removal. Therefore, at the time of the RapidEye satellite remote-sensing in 2012, only the 10 plots in the southern block had been subjected to the liana removal treatment. Nevertheless, to avoid the risk of missing effects of this treatment, we included the liana removal treatment in our statistical analysis but only as implemented at the time of data collection [i.e., the six plots in the northern block are treated as 16-species plots without liana removal for the purposes of the analyses reported in this article, as opposed to longer-term assessments reported elsewhere that assess the liana removal treatment as applied to all plots (*18*)].

In line with standard enrichment planting procedure, after the initial cohort of seedlings were planted (between January 2002 and September 2003), a second cohort was planted to replace initial mortalities (cohort 2 planted September 2008 to August 2009). In combination, the two cohorts planted and surveyed a total of 96,369 dipterocarp seedlings. Further details of the Malua reserve and the Sabah Biodiversity Experiment can be found in previous publications (*19*, *20*, *54*).

### Remote-sensing

Landsat Vegetation Continuous Fields tree cover, RapidEye, and MODIS imagery were selected to estimate variation in canopy structure based on the needs of data accuracy, the size of the study site and plots, and the time period of the experiment (table S10).

### Landsat vegetation continuous fields tree cover

The Landsat Continuous Fields tree cover (Landsat tree cover) estimates the percentage of horizontal ground per 30 m pixel, which is covered with vegetation of at least 5 m vertical height (*55*). In this study, we refer to Landsat tree cover as Landsat vegetation cover, as in Sabah virtually all vegetation detected by Landsat is higher than this minimum. The product is derived from all seven bands of Landsat-5 Thematic Mapper and/or Landsat Enhanced Thematic Mapper Plus. The partial resolution of the Landsat vegetation cover dataset is 30 m, which is appropriate for the Sabah Biodiversity Experiment’s plot size of 200 m by 200 m, giving c. 44 pixels per plot. This dataset contains three epochs, 2000, 2005, and 2010, each consisting of a composite of several years’ worth of images to minimize the effects of cloud cover. The 2000 epoch consists of data from 1999 to 2002, our 2005 epoch contains years 2003 to 2008, and the 2010 epoch ranges 2008 to 2012.

### MODIS MCD15A3H

MODIS MCD15A3H leaf area index is widely used in forest monitoring and exhibits very high accuracy (*56*–*58*). However, the spatial resolution of 500 m means that each plot does not even have a single complete pixel. Instead, a comparison of the entire Sabah Biodiversity Experiment site with the surrounding relogged area is reported elsewhere (*32*).

### RapidEye imagery

This study used a RapidEye satellite image of the Sabah Biodiversity Experiment site for August 2012. RapidEye imagery uses a higher spatial resolution of 5 m and a temporal resolution of 5.5 days (*59*, *60*). This multispectral scanner of the RapidEye satellites acquires data in five bands. The blue (0.44 to 0.51 μm), green (0.52 to 0.59 μm), red (0.63 to 0.68 μm), and near-infrared (0.76 to 0.85 μm) are very similar to that of the Landsat Spectral band equivalents, while also having an additional red-edge band (0.69 to 0.73 μm). This band allows RapidEye satellite images to provide greater sensitivity to spatiotemporal changes in vegetation (*61*, *62*).

### Vegetation metrics inversion from RapidEye image

A FLAASH atmospheric correction model was applied to the RapidEye image, and vegetation cover, leaf area index, and aboveground biomass were calculated using empirical formulae develop in Pfeifer *et al.* (*62*) (Eqs. 1, 2, and 3, respectively), which used RapidEye imagery of the nearby SAFE landscape. Although these equations were not derived for Malua (where the Sabah Biodiversity Experiment is located), the SAFE landscape is close by, and greatly more so than all other options. This provided us with high-resolution (5 m for RapidEye) estimates of leaf area index, vegetation cover, and aboveground biomass using a method developed and validated for lowland dipterocarp forests in the same part of Sabah. Further details of the inversion methodology used can be found in the previous publication (*62*).

$$\begin{array}{c} \text{Vegetation cover}=2.66-0.66\cdot Red+0.3\cdot RedEdge-0.08\;\cdot \\ NearIR-0.17\cdot DissB3+1.48\cdot DissB4-0.42\cdot DissB5 \end{array}$$ (1)

$$\begin{array}{c} \text{Leaf area index}=0.9-0.59\cdot Red+0.41\cdot RedEdge-0.11\;\cdot \\ NearIR-0.53\cdot DissB3+1.08\cdot DissB4-0.36\cdot DissB5 \end{array}$$ (2)

$$\begin{array}{c} \text{Aboveground biomass}=19.45-\exp(\mathrm{MSAV}12)-2.39\cdot Green\;+\\ 1.08\cdot RedEdge\;+2.65\cdot DissB2-0.28\cdot DissB3\;+0.09\cdot DissB4\;-\\ 0.13\cdot DissB5 \end{array}$$ (3)

where *MSAV12* is the Modified Soil-Adjusted Vegetation Index 2 (*63*). *Green*, *Red*, *RedEdge*, and *NearIR* all correspond to the RapidEye bands of the same name, and *DissB2*, *DissB3*, *DissB4*, and *DissB5* are the gray-level dissimilarities of green band, red band, near-infrared band, and red-edge band, respectively (*64*). Satellite imagery were preprocessed using ArcGIS and overlaid with the Sabah Biodiversity Experiment plot layout based on GPS coordinates were collected for the perimeter of each block.

### Landsat and RapidEye cover comparison

While the Landsat and RapidEye estimates of vegetation cover show the same qualitative relationships with the Sabah Biodiversity Experiment treatments they differ in the absolute value of the estimates. The two measures are positively correlated (Pearson product-moment correlation: 0.389, *P* = 7 × 10^−6^ and *df *= 122) but the Landsat cover estimates are greater than RapidEye cover estimates (by 9.76 ± 0.371%). There are at least three nonmutually exclusive explanations for these differences. First, the spatial resolution of Landsat vegetation cover is much lower than that of RapidEye, which means that less information can be extracted. However, it may be more difficult to extract information accurately from high–spatial resolution data because more kinds of information can have an influence that with lower spatial resolution, such as topography. Second, the RapidEye data were collected from a single time point in 2012, while the Landsat cover estimates are calculated across a range of years (epochs: for example, the 2010 epoch cover estimates come from the 2008–2012 period). Third, the calculation method of Landsat vegetation cover is developed for global coverage, while that of RapidEye is developed specifically for Borneo.

### Phylogenetic and functional diversity

To investigate the effects of a broader range of aspects of diversity we calculated measures of both phylogenetic and functional diversity. A phylogenetic tree was created using information specified in two papers detailing recent advancements in dipterocarp phylogeny (*65*, *66*). We calculated Faith’s PD (*29*), defined as the total branch length of the minimum spanning tree from each node to the tree root. We also calculated FD, a functional equivalent of Faith’s PD, by the sum of branch lengths on a functional dendrogram (*28*). Of 46 total trait measurements available, we opted a priori to use leaf nitrogen, leaf phosphorus, leaf thickness, dry weight, wood density, and specific leaf area as these measurements have been used to estimate FD in the literature most widely (*67*–*69*), and there are current associations with the trade-off between rapid resource acquisition and faster growth and enhanced environmental tolerance and reduced growth (*70*, *71*). This also avoided using a large number of partially correlated variables. Both PD and FD measurements are dependent on species richness and so contain information of phylogenetic and functional diversity both among and within species richness levels.

### Statistical analysis

We analyzed the satellite remote-sensing data using linear mixed-effects models that, after performing an initial overall test for the main experimental treatments, then implemented a series of a priori contrasts investigating the main comparisons of interest (fig. S5), specifically:

1. Replanting (enrichment-planted plots versus unplanted controls)

2. Species richness of enrichment-planted trees (1, 4, or 16 species)

3. Genus diversity (one, two, four, or five genera across the whole species richness gradient and two versus four genera for the balanced comparison within the four-species plots)

4. Predicted canopy structural complexity (mixtures of seedlings of species with similar predicted adult heights versus a greater diversity of adult tree heights)

5. Liana removal (climber cutting; 16-species plots with and without lianas removed)

The linear mixed-effects models were implemented using the lme4 (version 1.1-33) (*72*) and lmerTest (version 3.1-3) (*73*) packages for R (version 4.3.1) (*74*). As would be expected, the RapidEye estimates of vegetation cover, leaf area index, and aboveground biomass values were positively correlated with each other, so to reduce the number of statistical tests we focused on aboveground biomass when available (RapidEye) and vegetation cover when not (Landsat). Our design contains a “nested” series of comparisons (a two-factor factorial design is contained within the four-species enrichment planting treatment level, for example) requiring a series of models to address all of the a priori contrasts contained within the overall design. We first fitted a model containing a single fixed factor with levels for the main treatments (five levels: unplanted; 1-, 4-, and 16-species enrichment planting; 16-species with liana removal) and used a sequential type I analysis of variance with Satterthwaite degrees of freedom to test for differences among treatment levels before proceeding to fit a series of more focused single degree of freedom contrasts to extract point estimates with CIs for the comparisons contained within the overall design (enrichment-planted versus unenriched plots etc.) as specified below (models 1 to 6). Profile likelihood CIs were generated using the “confint” function within the “stats” package (version 4.3.1) (*74*) and were used as they generally outperform standard asymptotic normal CIs for mixed models (*75*). The marginal and conditional *R*-squared (*R2m* and *R2c*) values were created using the “R.squaredGLMM” function within the “MuMIn” package (version 1.47.5) (*76*). Note that post hoc adjustments for multiple comparisons were unnecessary due to the shrinkage involved in the point estimates and predictions produced by the mixed-effects models (*77*).

### Model 1: Initial overall test for differences among levels of the primary treatment

y∼Treatment+(1∣block)+(1∣Spp_comp)

where *y* is a continuous response variable (either aboveground biomass, vegetation cover, or leaf area index), treatment is a fixed factor with five levels (unplanted, 1, 4, 16 species, and 16-species with climber cutting), block is a random factor with two levels (northern or southern block), Spp_comp is a random factor with 33 levels (16 one-species compositions, 16 four-species compositions plus the full 16-species mixture), and (1|Factor) indicates random intercepts for levels of the factor in question.

### Model 2: Effect of planting (enrichment-planted plots versus unenriched controls)

y∼Planting+(1∣Block)+(1∣Spp_comp)

where *y* is as in model 1, planting is a fixed effect with two levels (enrichment-planted versus unplanted). This model fits the a priori contrast that compares the unenriched controls with all of the enrichment-planted treatment levels combined.

### Model 3: Linear (log_2_ scale) contrast for the number of enrichment-planted species

$$\begin{array}{c} y∼\log_{2}(\mathrm{Spp}\_\mathrm{richness})+\mathrm{factor}(\mathrm{Spp}\_\mathrm{richness})+\mathrm{Treatment}\;+\\ (1|\mathrm{block})+(1|\mathrm{Spp}\_\mathrm{comp}),\mathrm{subset}=\mathrm{Spp}\_\mathrm{richness}>0 \end{array}$$

where *y* is as in model 1, log2(Spp_richness) is a continuous fixed response variable for the (log2-transformed) number of enrichment-planted tree species (1, 4 or 16 species, excluding unplanted controls addressed by model 2), species richness as a factor fitted sequentially after the continuous response variable captures deviations from log-linearity (*5*), and treatment subsequently captures the effects of liana removal.

### Model 4: Effect of species richness for each of three Landsat time periods:

$$\begin{array}{c} y∼\mathrm{factor}(\mathrm{Spp}\_\mathrm{richness})*\mathrm{Year}+(1|\mathrm{block})\;+\\ (1|\mathrm{Spp}\_\mathrm{comp})+(1|\mathrm{Plot}) \end{array}$$

where *y* was Landsat estimated percent canopy cover (only), year is a fixed factor with 3 levels, and the asterisk (*) indicates an interaction between variables. Since there are three repeated measures per plot, one for each Landsat time period, we also added a random factor with a level for each plot (124 levels).

### Model 5: Effect of climber cutting, genus diversity, and liana removal

$$\begin{array}{c} y∼\mathrm{factor}(\mathrm{Spp}\_\mathrm{richness})+\mathrm{Gen}\_\mathrm{div}+\mathrm{Canopy}\_\mathrm{type}\;+\\ \mathrm{Climber}\_\mathrm{cutting}+(1|\mathrm{block})+(1|\mathrm{Spp}\_\mathrm{comp}) \end{array}$$

where *y* is as in model 1, Spp_richness is a factor with three levels (1, 4, and 16), Gen_div is a fixed factor with two levels (2 versus 4 genera), Canopy_type is a fixed factor with two levels (low versus high canopy structural complexity, and Climber_cutting is a fixed factor with 2 levels (liana removed versus not).

### Model 6: Effect of PD/FD

y∼Diversity+(1∣block)+(1∣Spp_comp)

where diversity is either phylogenetic or FD, each fitted as a continuous response variable. To identify an effect within just the four-species plots, this model was run with all data initially and then subsequently with a subset of only four-species plots. As these measures of FD and PD were not controlled components of the original experimental manipulation other fixed effects addressed in previous models have been omitted for simplicity (an approach that should be conservative as the variation explained by the omitted variables ends up in the error terms).
