# Supplementary material for: Positive effects of tree diversity on tropical forest restoration in a field-scale experiment
Source: Sci Adv. 2023 Sep 15;9(37):eadf0938. doi: 10.1126/sciadv.adf0938 (PMC10846868; doi:10.1126/sciadv.adf0938)
Supplement: Supplementary file 1 — Figs. S1 to S5 Tables S1 to S10 Legend for other file [file sciadv.adf0938_sm.pdf]

Science Advances
AAAS

Supplementary Materials for

Positive effects of tree diversity on tropical forest restoration in a field-scale experiment

Ryan Veryard *et al.*

Corresponding author: Andy Hector, andrew.hector@biology.ox.ac.uk

Sci. Adv. **9**, eadf0938 (2023)
DOI: 10.1126/sciadv.adf0938

The PDF file includes:

Figs. S1 to S5
Tables S1 to S10
Legend for other file

Other Supplementary Material for this manuscript includes the following:

Other file

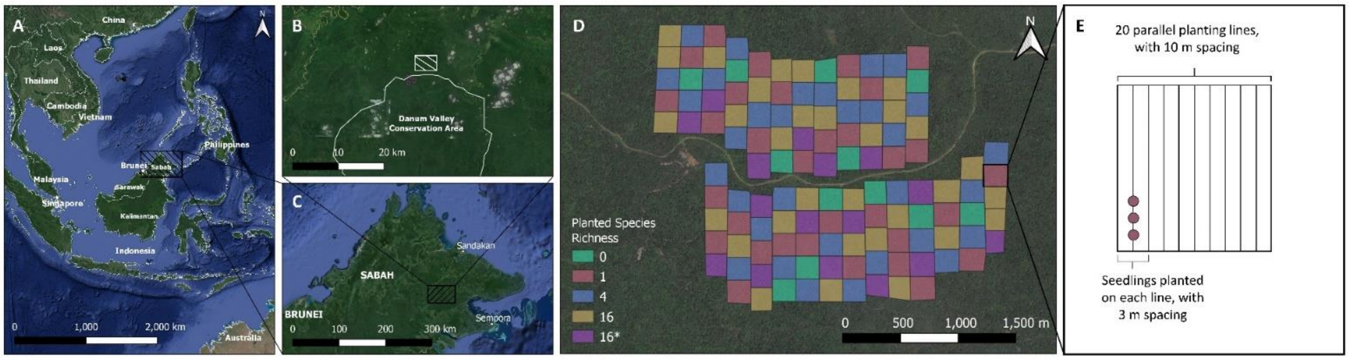

Fig. S1. Location and design of the Sabah Biodiversity Experiment. (**A**) The Sabah Biodiversity Experiment is located in Sabah, Malaysian Borneo, within South East Asia. (**B**, **C**) The experimental site is located in a selectively logged area of the Malua Forest Reserve just north of the Danum Valley Conservation Area, Sabah, Malaysian Borneo. (**D**) The Sabah Biodiversity Experiment consists of 124 four-hectare plots, separated into two blocks by a logging road, with a combination of treatments that vary the species richness of enrichment-planted dipterocarp seedlings (0, 1, 4, or 16 species) and restoration methodologies (liana removal (16*) or not (16)). Note that at the time of the RapidEye satellite remote sensing in 2012 only the ten plots in the southern block had been subjected to the liana removal treatment. The six liana removal plots in the northern block are therefore treated as 16-species plots without liana removal in this analysis. (**E**) In each plot, dipterocarp seedlings were planted every 3 m along parallel lines 10 m apart.

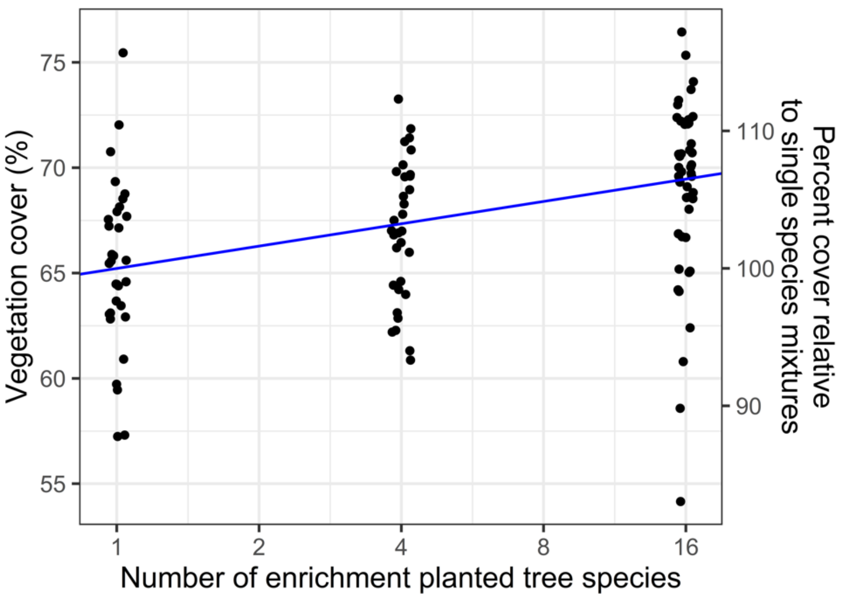

Fig. S2. Effect of diversity on enrichment planted dipterocarp estimated vegetation cover. Estimated vegetation cover (RapidEye) as a function of the number of enrichment-planted tree species a decade after initial planting. The line is the regression slope with the $\log_2$ number of tree species from the mixed-effects model analysis (points are jittered to avoid overlap).

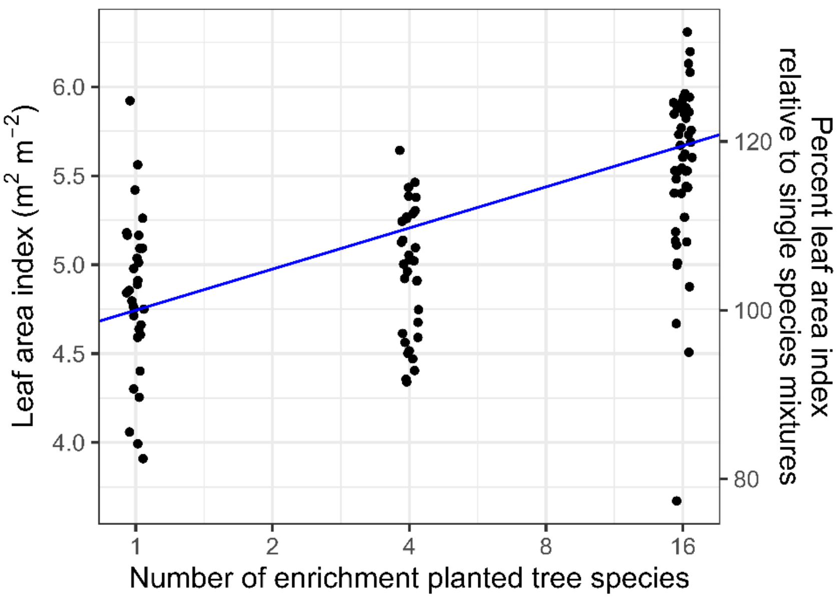

Fig. S3. Effect of diversity on enrichment planted tree's estimated leaf area index. Estimated leaf area index (RapidEye) as a function of the number of enrichment-planted tree species a decade after initial planting. The line is the regression slope with the $\log_2$ number of tree species from the mixed-effects model analysis (points are jittered to avoid overlap).

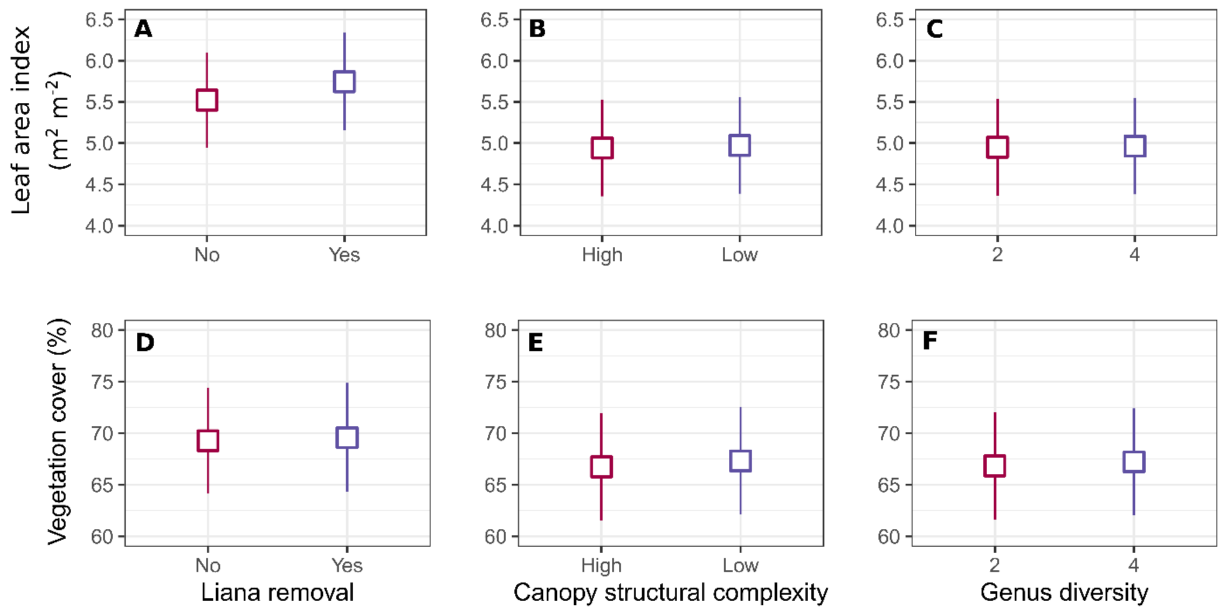

Fig. S4. RapidEye satellite remote sensing estimates as a function of restoration treatment a decade after initial planting. leaf area index (**A** to **C**) and cover (**D** to **F**) as a function of (from left to right) genus diversity of plots enrichment planted with four-species (2 genera vs 4 genera); canopy complexity with four species (low vs high); and liana ('climber') cutting with 16 species.

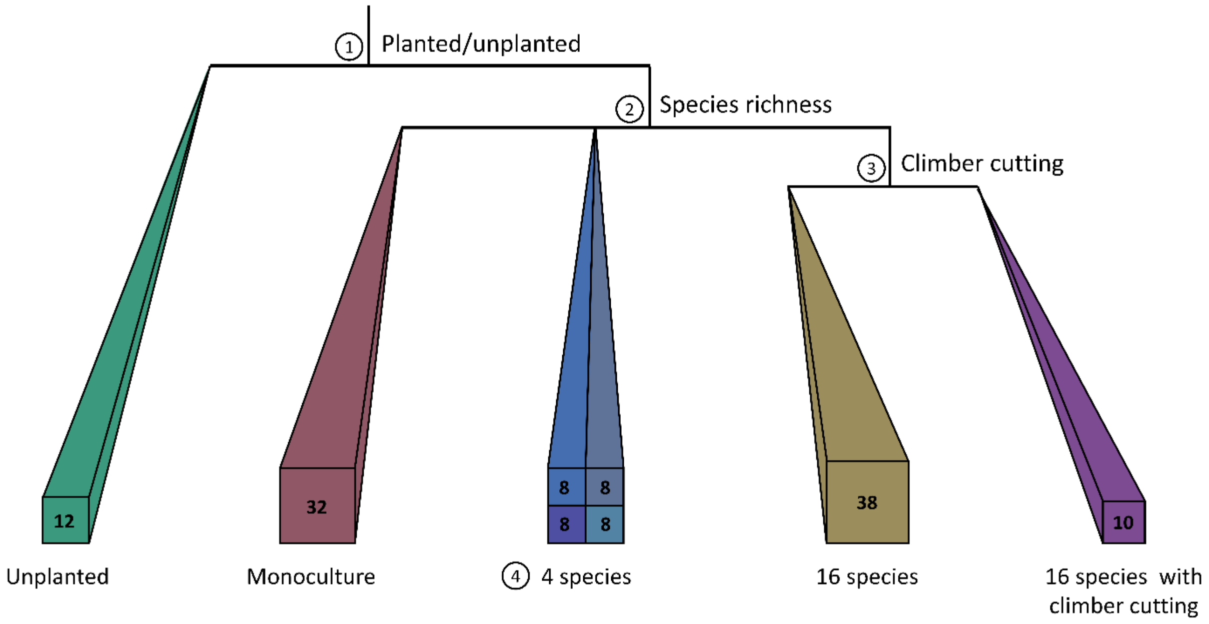

Fig. S5. Summary of comparisons made by the mixed-effects model analysis. After performing an initial overall test for differences among treatment levels we performed a series of *a priori* contrasts for the comparisons of interest contained within the overall design: 1) enrichment-plant vs. unplanted control plots; 2) Number (richness) of species of enrichment planted tree (both as a factor with three levels and as a regression on the $\log_2$ number of species; 3) Effects of climber cutting (16-species plots only); 4) A two-factor factorial comparison (within the 4-species plots only) of the effects of canopy structural complexity (higher and lower) and genus diversity (4 species drawn from 2 versus 4 genera).

Table S1. The 16 species of the Dipterocarpaceae family studied by the Sabah Biodiversity Experiment with relevant traits

Species	Relative canopy height	Ecology	Timber group	IUCN status
Dipterocarpus conformis Slooten	Tall	Rare, Hill dipterocarp forest, clay rich soils, below 800 m	-	Endangered
Dryobalanops lanceolata Burck	Tall	Widespread on fertile soils, abundant on undulating land on volcanic/calcareous soils, up to 700 m	-	Least Concern
Hopea ferruginea Parijs	Short	Deep fertile soils in mixed dipterocarp forest, below 750 m	-	Critically endangered
Hopea sangal Korth.	Short	Often on or near riverbanks in low country and up to 500 m	-	Vulnerable
Parashorea Malaanonan (Blanco) Merr.	Tall	Abundant in lowlands, typically E. Sabah, recorded up to 1300 m	-	Least concern
Parashorea tomentella (Symington) Meijer	Tall	Common on flat and undulating land, up to 200 m	-	Least concern
Shorea argentifolia Symington	Medium	Locally frequent in forests, especially clay soils on undulating land and in valleys, below 600 m	Red Meranti	Least concern
Shorea beccariana Bruck	Medium	Common on leached lowland soils and dry ridges up to 1350 m	Red Meranti	Least Concern
Shorea faguetiana Heim.	Tall	Well-drained clay soils on low hills, and particularly ridge tops at 150-100 m (typically 700 m)	Yellow Meranti	Endangered
Shorea gibbosa Brandis	Tall	Common on deep fertile soils, below 600 m	Yellow Meranti	Critically endangered
Shorea johorensis Foxw.	Tall	E. Borneo on well-drained fertile soils, below 600 m	Red Meranti	Critically endangered
Shorea leprosula Miq.	Medium	Deep clay soils in mixed dipterocarp forest below 700 m	Red Meranti	Near-threatened
Shorea macrophylla Ashton	Medium	Locally abundant on periodically flooded alluvium and riverbanks but rarer on hillsides, below 600 m	Red Meranti	Least Concern
Shorea macroptera King	Medium	Clay soils on low hills up to 600 m	Red Meranti	Least Concern
Shorea ovalis Korth.	Medium	Scattered in mixed dipterocarp forests, usually in moist or low-lying ground, up to 500 m	Red Meranti	Least Concern
Shorea parvifolia Dyer	Medium	Perhaps the commonest dipterocarp, on clay soils on hills below 800 m	Red Meranti	Least Concern

Table S2. Mixed-effects model fixed effects estimates of aboveground biomass (AGB), leaf area index (LAI), and % vegetation cover. Columns list treatment level, sample size and estimated means for each level of the primary treatment with upper and lower bounds of likelihood profile 95% confidence intervals. Mixed-effects models ANOVA *F*-tests of the treatment factor (5 levels; Satterthwaite degrees of freedom) are: AGB: $F_{4,118.5} = 132.39$, $P = < 2.2e\text{-}16$; LAI: $F_{4,118.1} = 31.84$, $P = < 2.2e\text{-}16$; Cover: $F_{4,118.11} = 14.772$, $P = 8.17e\text{-}10$. R-squared values (R2m and R2c) are for the model with all treatments included.

Index	Treatment	Number of plots	Estimate	CI 2.5% limit	CI 97.5% limit	R2m	R2c
AGB (Mg ha-1)	Unplanted	12	182.67	174.39	190.94	0.780	0.815
	Monoculture	32	213.89	206.99	220.79	-	-
	4 species	32	231.90	225.01	238.80	-	-
	16 species	38	261.55	254.51	268.49	-	-
	16 species with liana removal	10	265.73	256.70	274.68	-	-
LAI (m^2 m^{-2})	Unplanted	12	4.57	3.99	5.16	0.364	0.641
	Monoculture	32	4.82	4.24	5.40	-	-
	4 species	32	4.96	4.44	5.47	-	-
	16 species	38	5.52	5.01	6.03	-	-
	16 species with liana removal	10	5.74	5.21	6.29	-	-
Cover (%)	Unplanted	12	62.05	56.81	67.28	0.210	0.563
	Monoculture	32	65.33	60.19	70.47	-	-
	4 species	32	67.03	61.89	72.17	-	-
	16 species	38	69.27	64.13	74.39	-	-
	16 species with liana removal	10	69.57	64.32	74.88	-	-

Table S3. Estimates of AGB, LAI and % vegetation cover for unplanted vs planted plots with the mean differences and 95% CI limits. R-squared values (R2m and R2c) are for the model with all treatments included.

Index	Treatment	Number of plots	Estimate	CI 2.5% limit	CI 97.5% limit	*R2m*	*R2c*
AGB (Mg ha^{-1})	Unplanted	12	182.67	153.06	212.27	0.303	0.705
	Planted	110	225.99	217.50	234.55	-	-
	Difference	-	43.33	13.33	73.20	-	-
LAI (m^2 m^{-2})	Unplanted	12	4.57	3.89	5.26	0.042	0.572
	Planted	110	4.96	4.40	5.53	-	-
	Difference	-	0.39	-0.18	0.95	-	-
Cover (%)	Unplanted	12	62.05	56.63	67.47	0.082	0.519
	Planted	110	66.67	61.71	71.68	-	-
	Difference	-	4.63	1.08	8.07	-	-

Table S4. AGB, LAI, and % vegetation cover changes per doubling in species richness (i.e. slope of the mixed-effects regression slopes versus species richness (log-2 transformed)

Dataset	Change per doubling in species richness	CI 2.5% limit	CI 97.5% limit	*R2m*	*R2c*
AGB (Mg ha^{-1})	12.89	10.33	15.09	0.715	0.732
LAI (m^2 m^{-2})	0.23	0.16	0.30	0.322	0.628
Cover (%)	1.06	0.44	1.66	0.126	0.513

Table S5. % vegetation cover estimates per time period and species richness group with 95% CI limits. Mixed-effects model *R2m* = 0.734, *R2c* = 0.920.

Time period	Species richness	Cover estimate (%)	CI 2.5% limit	CI 97.5% limit
1999-2002	0	71.95	71.21	72.70
1999-2002	1	72.51	72.05	72.97
1999-2002	4	72.47	72.01	72.92
1999-2002	16	72.46	72.01	72.76
2003-2008	0	72.46	71.71	73.20
2003-2008	1	75.44	74.98	75.89
2003-2008	4	75.39	74.93	75.85
2003-2008	16	76.42	75.97	76.71
2008-2012	0	72.51	71.77	73.25
2008-2012	1	75.20	74.74	75.66
2008-2012	4	77.03	76.57	77.49
2008-2012	16	78.59	78.13	79.07

Table S6. 4-species mixture compositions

Plot ID	Genera number	Structural complexity	Species 1	Species 2	Species 3	Species 4
4.1	2	Low	*P. malaanonan*	*P. tomentella*	*S. beccariana*	*S. leprosula*
4.2	2	Low	*P. malaanonan*	*P. tomentella*	*S. macroptera*	*S. ovalis*
4.3	2	Low	*H. sangal*	*H. ferruginea*	*S. macrophylla*	*S. parvifolia*
4.4	2	Low	*H. sangal*	*H. ferruginea*	*S. argentifolia*	*S. parvifolia*
4.5	2	High	*H. sangal*	*H. ferruginea*	*S. beccariana*	*S. johorensis*
4.6	2	High	*H. sangal*	*H. ferruginea*	*S. macroptera*	*S. gibbosa*
4.7	2	High	*H. sangal*	*H. ferruginea*	*S. macrophylla*	*S. faguetiana*
4.8	2	High	*H. sangal*	*H. ferruginea*	*S. argentifolia*	*S. johorensis*
4.9	4	Low	*D. conformis*	*D. lanceolata*	*P. malaanonan*	*S. faguetiana*
4.10	4	Low	*D. conformis*	*D. lanceolata*	*P. tomentella*	*S. johorensis*
4.11	4	Low	*D. conformis*	*D. lanceolata*	*P. tomentella*	*S. gibbosa*
4.12	4	Low	*D. conformis*	*D. lanceolata*	*P. malaanonan*	*S. johorensis*
4.13	4	High	*D. conformis*	*D. lanceolata*	*S. macrophylla*	*H. sangal*
4.14	4	High	*D. conformis*	*D. lanceolata*	*S. ovalis*	*H. ferruginea*
4.15	4	High	*D. conformis*	*D. lanceolata*	*S. ovalis*	*H. sangal*
4.16	4	High	*D. conformis*	*D. lanceolata*	*S. macrophylla*	*H. ferruginea*

Table S7. AGB, LAI, and % vegetation cover estimates (with 95% CI limits) by genus diversity and canopy structural complexity combinations, within 4-species plot combinations. R-squared values (R2m and R2c) are for the model with all treatments included.

Index	Genera	Canopy structural complexity	Number of plots	Estimate	CI 2.5% limit	CI 97.5% limit	R2m	R2c
AGB (Mg ha-1)	2 genera	Low	8	231.53	222.99	240.07	0.798	0.813
		High	8	228.93	220.43	237.44	-	-
	4 genera	Low	8	234.86	226.32	243.40	-	-
		High	8	232.26	223.72	240.80	-	-
LAI (m^2 m^{-2})	2 genera	Low	8	4.97	4.37	5.56	0.362	0.647
		High	8	4.93	4.34	5.52	-	-
	4 genera	Low	8	4.98	4.39	5.57	-	-
		High	8	4.95	4.36	5.54	-	-
Cover (%)	2 genera	Low	8	67.12	61.87	72.37	0.210	0.560
		High	8	66.55	61.29	71.80	-	-
	4 genera	Low	8	67.50	62.25	72.76	-	-
		High	8	66.93	61.68	72.19	-	-

Table S8. AGB, LAI, and % vegetation cover estimates (with 95% CI limits) for untreated and liana-removed 16-species plots. R-squared values (R2m and R2c) are for the model with all treatments included.

Index	Liana removal	Number of plots	Estimate	CI 2.5% limit	CI 97.5% limit	R2m	R2c
AGB (Mg ha-1)	No	38	261.55	254.51	268.49	0.798	0.813
	Yes	10	265.73	256.71	274.81	-	-
	Difference	-	4.18	-5.62	12.73	-	-
LAI (m^2 m^{-2})	No	38	5.52	4.94	6.10	0.362	0.647
	Yes	10	5.74	5.15	6.34	-	-
	Difference	-	0.22	-0.05	0.48	-	-
Cover (%)	No	38	69.27	64.13	74.39	0.210	0.560
	Yes	10	69.57	64.32	74.88	-	-
	Difference	-	0.30	-2.13	2.63	-	-

Table S9. Aboveground biomass change per unit increase in Functional Diversity (FD) and Phylogenetic Diversity (PD) with 95% CI limits.

Index	Species richness range	Change per unit Index increase (Mg ha^{-1})	CI 2.5% limit	CI 97.5% limit	R2m	R2c
FD	1, 4, 16	12.44	9.15	15.57	0.609	0.745
FD	4 only	4.22	-44.35	52.78	0.001	0.118
PD	1, 4, 16	605.10	517.53	676.92	0.700	0.712
PD	4 only	116.97	-99.43	333.37	0.032	0.151

Table S10. Remote sensing datasets used in this study.

Data	Spatial Resolution	Temporal Resolution	Time Cover
Landsat VCF vegetation cover	30 m	5-year	2000, 2005, 2010*
RapidEye-based vegetation cover	5 m	-	Aug. 2012
RapidEye-based leaf area index	5 m	-	Aug. 2012
RapidEye-based aboveground biomass	5 m	-	Aug. 2012
MODIS MCD15A3H LAI	500 m	4-day	2001-2017

***Mid-points of the monitoring time periods ('epochs') – see main text for details.**

Supplementary auxiliary files:

Supplementary material - analysis

RMarkdown for paper
