# Supplementary material for: Positive effects of tree diversity on tropical forest restoration in a field-scale experiment
Source: Sci Adv. 2023 Sep 15;9(37):eadf0938. doi: 10.1126/sciadv.adf0938 (PMC10846868; doi:10.1126/sciadv.adf0938)

Positive effects of tree diversity on tropical forest restoration in a field-scale experiment
Supplementary material - analysis

Ryan Veryard

06 April, 2023

Contents

1 Introduction **2**

2 Read and Check Data **2**

2.1 `Sat_data` import 2

2.2 `Rapideye_data` and `Landsat_data` import 4

2.3 Descriptive summary of data 6

3 Effect of planting treatment **8**

3.1 Model output 9

4 Effect of planting **15**

4.1 Modelling 15

4.2 Graphing 17

5 Effect of planted species richness **19**

5.1 Modelling 19

5.2 Graphing 20

6 Change in response to planted species richness over time **24**

6.1 Modelling 24

6.2 Graphing 28

7 Effect of genus diversity and canopy strucutral complexity **29**

7.1 Modelling 29

7.2 Graphing 34

8 Effect of phylogenetic and functional diversity **42**

8.1 Modelling . . . 42

8.2 Graphing . . . 44

9 Effect of liana removal **46**

9.1 Modelling . . . 46

9.2 Graphing . . . 49

1 Introduction

This document accompanies the research article `Positive effects of tree diversity on tropical forest restoration in a field-scale experiment`.

For a detailed overview of the experimental design of this study, please see the supplementary methods document.

Our analysis was performed in R (version 3.6.3), using primarily the tidyverse (version 1.2.1) and lme4 (version 1.1-21) packages.

2 Read and Check Data

Read in the data files used in this analysis, and check that it is encoded correctly.

```
library(scales)
library(lme4)
library(RColorBrewer)
library(lmerTest)
library(tidyverse)
library(picante)

select <- dplyr::select
theme_set(theme_bw())

cols <- c(brewer.pal(11, "Spectral")[1], brewer.pal(11,
    "Spectral")[11])
```

2.1 Sat_data import

First the main dataset is loaded in, which combines the Landsat and RapidEye data in a single object.

```
Sat_data <- readRDS("Data/SBE_Data.rds")
```

```
str(Sat_data)
```

```
## tibble [744 x 17] (S3: tbl_df/tbl/data.frame)
##  $ Block          : Factor w/ 2 levels "North","South": 2 2 2 2 2 2 2 2 2 2 ...
##  $ Plot           : Factor w/ 124 levels "1","2","3","4",..: 1 1 1 1 1 1 6 6 6 6 ...
```

```
##  $ Spp_richness   : num [1:744] 4 4 4 4 4 4 4 4 4 4 ...
##  $ Spp_comp       : Factor w/ 35 levels "0_Unplanted",..: 24 24 24 24 24 24 32 32 32 32 ...
##  $ Climber_cutting: Factor w/ 2 levels "No","Yes": 1 1 1 1 1 1 1 1 1 1 ...
##  $ Satellite      : Factor w/ 2 levels "Landsat","RapidEye": 1 1 1 2 2 2 1 1 1 2 ...
##  $ Index          : Factor w/ 3 levels "Biomass","Cover",..: 2 2 2 3 1 2 2 2 2 3 ...
##  $ Year           : Factor w/ 3 levels "2000","2005",..: 1 2 3 NA NA NA 1 2 3 NA ...
##  $ Score          : num [1:744] 72.13 73.94 77.17 4.92 233.15 ...
##  $ Gen_div        : Factor w/ 4 levels "0","2","4","5": 3 3 3 3 3 3 2 2 2 2 ...
##  $ Canopy_type    : Factor w/ 3 levels "0","Thin","Thick": 3 3 3 3 3 3 3 3 3 3 ...
##  $ Canopy_str     : Factor w/ 7 levels "0","S","M","T",..: 7 7 7 7 7 7 7 7 7 7 ...
##  $ Planting       : Factor w/ 2 levels "Control","Planted": 2 2 2 2 2 2 2 2 2 2 ...
##  $ Treatment      : Factor w/ 5 levels "0_spp","1_spp",..: 3 3 3 3 3 3 3 3 3 3 ...
##  $ Random         : Factor w/ 2 levels "No","Yes": 2 2 2 2 2 2 2 2 2 2 ...
##  $ PD             : num [1:744] 0.0655 0.0655 0.0655 0.0655 0.0655 ...
##  $ FD             : num [1:744] 2.2 2.2 2.2 2.2 2.2 ...
```

```
summary(Sat_data)
```

```
##    Block          Plot      Spp_richness                         Spp_comp
##  North:360   1      :  6   Min.   : 0.000   16_spp                   :228
##  South:384   2      :  6   1st Qu.: 1.000   0_Unplanted              : 72
##              3      :  6   Median : 4.000   16_cut                   : 60
##              4      :  6   Mean   : 7.484   1_Dipterocarpus_conformis: 12
##              5      :  6   3rd Qu.:16.000   1_Dryobalanops_lanceolate: 12
##              6      :  6   Max.   :16.000   1_Hopea_ferruginea       : 12
##              (Other):708                    (Other)                  :348
##  Climber_cutting    Satellite       Index       Year          Score
##  No :684         Landsat :372   Biomass:124   2000:124   Min.   :  3.671
##  Yes: 60         RapidEye:372   Cover  :496   2005:124   1st Qu.: 67.436
##                                 LAI    :124   2010:124   Median : 73.481
##                                               NA's:372   Mean   : 88.475
##                                                          3rd Qu.: 77.374
##                                                          Max.   :290.831
##
##  Gen_div Canopy_type Canopy_str    Planting    Treatment   Random
##  0: 72   0    : 72   0  : 72    Control: 72   0_spp : 72   No :132
##  2:288   Thin :288   S  : 24    Planted:672   1_spp :192   Yes:612
##  4: 96   Thick:384   M  : 84                  4_spp :192
##  5:288               T  :120                  16_spp:228
##                      SM : 24                  16_cut: 60
##                      MT : 36
##                      SMT:384
##        PD                FD
##  Min.   :0.01467   Min.   :0.000
##  1st Qu.:0.01846   1st Qu.:0.000
##  Median :0.05935   Median :2.095
##  Mean   :0.05880   Mean   :1.932
##  3rd Qu.:0.09438   3rd Qu.:3.594
##  Max.   :0.09438   Max.   :3.594
##  NA's   :72
```

2.2 Rapideye_data and Landsat_data import

For ease of some of the analyses contained within this document, the original `Sat_data` dataset has been divided into two smaller datasets, `Rapideye_data` and `Landsat_data`, representing data from the RapidEye and Landsat Satellites. More detail of how these two dataset structures is explained below.

```
Rapideye_data <- readRDS("Data/RapidEye_SBE_Data.rds")
```

```
str(Rapideye_data)
```

```
## tibble [124 x 16] (S3: tbl_df/tbl/data.frame)
##  $ Block          : Factor w/ 2 levels "North","South": 2 2 2 2 2 2 2 2 2 2 ...
##  $ Plot           : Factor w/ 124 levels "1","2","3","4",..: 1 6 9 12 16 19 25 28 35 39 ...
##  $ Spp_richness   : num [1:124] 4 4 4 4 4 4 4 4 4 4 ...
##  $ Spp_comp       : Factor w/ 35 levels "0_Unplanted",..: 24 32 29 21 22 31 28 33 23 35 ...
##  $ Climber_cutting: Factor w/ 2 levels "No","Yes": 1 1 1 1 1 1 1 1 1 1 ...
##  $ Gen_div        : Factor w/ 4 levels "0","2","4","5": 3 2 2 3 3 2 2 2 3 3 ...
##  $ Canopy_type    : Factor w/ 3 levels "0","Thin","Thick": 3 3 2 2 2 3 2 3 2 2 ...
##  $ Canopy_str     : Factor w/ 7 levels "0","S","M","T",..: 7 7 5 4 4 7 6 7 6 4 ...
##  $ Planting       : Factor w/ 2 levels "Control","Planted": 2 2 2 2 2 2 2 2 2 2 ...
##  $ Treatment      : Factor w/ 5 levels "0_spp","1_spp",..: 3 3 3 3 3 3 3 3 3 3 ...
##  $ Random         : Factor w/ 2 levels "No","Yes": 2 2 2 2 2 2 2 2 2 2 ...
##  $ PD             : num [1:124] 0.0655 0.0404 0.0371 0.0564 0.0574 ...
##  $ FD             : num [1:124] 2.2 2.05 2.03 2.14 2.17 ...
##  $ LAI            : num [1:124] 4.92 5.27 4.96 4.59 5.09 ...
##  $ Biomass        : num [1:124] 233 234 226 247 242 ...
##  $ Cover          : num [1:124] 66.4 69.6 67 66.2 68.3 ...
```

```
summary(Rapideye_data)
```

```
##    Block          Plot      Spp_richness                          Spp_comp
##  North:60   1       :  1   Min.   : 0.000   16_spp                    :38
##  South:64   2       :  1   1st Qu.: 1.000   0_Unplanted               :12
##             3       :  1   Median : 4.000   16_cut                    :10
##             4       :  1   Mean   : 7.484   1_Dipterocarpus_conformis: 2
##             5       :  1   3rd Qu.:16.000   1_Dryobalanops_lanceolate: 2
##             6       :  1   Max.   :16.000   1_Hopea_ferruginea       : 2
##             (Other):118                     (Other)                  :58
##  Climber_cutting Gen_div Canopy_type Canopy_str    Planting    Treatment
##  No :114         0:12    0    :12    0  :12     Control: 12   0_spp :12
##  Yes: 10         2:48    Thin :48    S  : 4     Planted:112   1_spp :32
##                  4:16    Thick:64    M  :14                   4_spp :32
##                  5:48                T  :20                   16_spp:38
##                                      SM : 4                   16_cut:10
##                                      MT : 6
##                                      SMT:64
##  Random        PD                  FD               LAI             Biomass
##  No : 22   Min.   :0.01467   Min.   :0.000   Min.   :3.671   Min.   :167.4
##  Yes:102   1st Qu.:0.01846   1st Qu.:0.000   1st Qu.:4.704   1st Qu.:215.2
##            Median :0.05935   Median :2.095   Median :5.103   Median :234.7
##            Mean   :0.05880   Mean   :1.932   Mean   :5.114   Mean   :234.2
##            3rd Qu.:0.09438   3rd Qu.:3.594   3rd Qu.:5.528   3rd Qu.:259.6
```

```
##           Max.   :0.09438   Max.   :3.594   Max.   :6.308   Max.   :290.8
##           NA's   :12
##      Cover
##  Min.   :54.16
##  1st Qu.:64.19
##  Median :67.37
##  Mean   :66.93
##  3rd Qu.:70.06
##  Max.   :76.45
##
```

```
Landsat_data <- readRDS("Data/Landsat_SBE_Data.rds")
```

```
str(Landsat_data)
```

```
## tibble [372 x 15] (S3: tbl_df/tbl/data.frame)
##  $ Block          : Factor w/ 2 levels "North","South": 2 2 2 2 2 2 2 2 2 2 ...
##  $ Plot           : Factor w/ 124 levels "1","2","3","4",..: 1 1 1 6 6 6 9 9 9 12 ...
##  $ Spp_richness   : num [1:372] 4 4 4 4 4 4 4 4 4 4 ...
##  $ Spp_comp       : Factor w/ 35 levels "0_Unplanted",..: 24 24 24 32 32 32 29 29 29 21 ...
##  $ Climber_cutting: Factor w/ 2 levels "No","Yes": 1 1 1 1 1 1 1 1 1 1 ...
##  $ Year           : Factor w/ 3 levels "2000","2005",..: 1 2 3 1 2 3 1 2 3 1 ...
##  $ Cover          : num [1:372] 72.1 73.9 77.2 73.5 77.4 ...
##  $ Gen_div        : Factor w/ 4 levels "0","2","4","5": 3 3 3 2 2 2 2 2 2 3 ...
##  $ Canopy_type    : Factor w/ 3 levels "0","Thin","Thick": 3 3 3 3 3 3 2 2 2 2 ...
##  $ Canopy_str     : Factor w/ 7 levels "0","S","M","T",..: 7 7 7 7 7 7 5 5 5 4 ...
##  $ Planting       : Factor w/ 2 levels "Control","Planted": 2 2 2 2 2 2 2 2 2 2 ...
##  $ Treatment      : Factor w/ 5 levels "0_spp","1_spp",..: 3 3 3 3 3 3 3 3 3 3 ...
##  $ Random         : Factor w/ 2 levels "No","Yes": 2 2 2 2 2 2 2 2 2 2 ...
##  $ PD             : num [1:372] 0.0655 0.0655 0.0655 0.0404 0.0404 ...
##  $ FD             : num [1:372] 2.2 2.2 2.2 2.05 2.05 ...
```

```
summary(Landsat_data)
```

```
##    Block          Plot      Spp_richness                          Spp_comp
##  North:180   1       :  3   Min.   : 0.000   16_spp                    :114
##  South:192   2       :  3   1st Qu.: 1.000   0_Unplanted               : 36
##              3       :  3   Median : 4.000   16_cut                    : 30
##              4       :  3   Mean   : 7.484   1_Dipterocarpus_conformis:  6
##              5       :  3   3rd Qu.:16.000   1_Dryobalanops_lanceolate:  6
##              6       :  3   Max.   :16.000   1_Hopea_ferruginea       :  6
##              (Other):354                     (Other)                   :174
##  Climber_cutting   Year         Cover        Gen_div Canopy_type Canopy_str
##  No :342         2000:124   Min.   :68.77   0: 36   0    : 36   0  : 36
##  Yes: 30         2005:124   1st Qu.:72.87   2:144   Thin :144   S  : 12
##                  2010:124   Median :75.06   4: 48   Thick:192   M  : 42
##                             Mean   :74.86   5:144               T  : 60
##                             3rd Qu.:76.71                       SM : 12
##                             Max.   :81.45                       MT : 18
##                                                                 SMT:192
##     Planting     Treatment    Random          PD                FD
##  Control: 36   0_spp : 36   No : 66   Min.   :0.01467   Min.   :0.000
```

```
##  Planted:336   1_spp : 96   Yes:306   1st Qu.:0.01846   1st Qu.:0.000
##                4_spp : 96             Median :0.05935   Median :2.095
##                16_spp:114             Mean   :0.05880   Mean   :1.932
##                16_cut: 30             3rd Qu.:0.09438   3rd Qu.:3.594
##                                       Max.   :0.09438   Max.   :3.594
##                                       NA's   :36
```

2.3 Descriptive summary of data

The structure of the `Sat_data` dataset incorporates the key elements of the Sabah Biodiversity Experiment Study Design.

For ease of analysis, Landsat and RapidEye datasets were separated into different datasets. Landsat vegetation cover scores were recorded in the column `Score` within the `Landsat_data` dataset, and `Year` specifies each epoch, encoded as a factor. Within the `Rapideye_data` dataset a separate response column stores each of the `Biomass`, `Cover`, and `LAI` values.

Finally, each dataset contains two columns representing calculated phylogenetic and functional diversity measures for each plot's planted species richness:

- Faith's PD [`PD`].
- Functional diversity (as calculated by `Petchy & Gaston (2002)`) [`FD`].

A descriptive summary of the major differences between RapidEye and Landsat data can be seen below:

Data	Spatial Resolution	Temporal Resolution	Time Cover
Landsat VCF vegetation cover	30 m	5-year	2000, 2005, 2010
RapidEye-based vegetation cover	5 m	/	Aug. 2012
RapidEye-based leaf area index (LAI)	5 m	/	Aug. 2012
RapidEye-based aboveground biomass	5 m	/	Aug. 2012

We have the correct number of treatment groups, as specified in experimental design. Although we expect 16 plots of the treatment type `16_cut`, since the 6 plots within the southern block were *de facto* uncut during August 2012 (when the RapidEye imagery was taken), those plots are classified here as `16_spp`.

```
Rapideye_data %>%
    group_by(Treatment) %>%
    summarise(n())
```

```
## # A tibble: 5 x 2
##   Treatment `n()`
##   <fct>     <int>
## 1 0_spp        12
## 2 1_spp        32
## 3 4_spp        32
## 4 16_spp       38
## 5 16_cut       10
```

We obtain, as expected, three times this in the `Landsat_data` dataset and six times these values in the `Sat_data` dataset.

```
Landsat_data %>%
    group_by(Treatment) %>%
    summarise(n())
```

```
## # A tibble: 5 x 2
##   Treatment `n()`
##   <fct>     <int>
## 1 0_spp        36
## 2 1_spp        96
## 3 4_spp        96
## 4 16_spp      114
## 5 16_cut       30
```

```
Sat_data %>%
    group_by(Treatment) %>%
    summarise(n())
```

```
## # A tibble: 5 x 2
##   Treatment `n()`
##   <fct>     <int>
## 1 0_spp        72
## 2 1_spp       192
## 3 4_spp       192
## 4 16_spp      228
## 5 16_cut       60
```

There are no inaccuracies with labelling between species composition, expected species richness, and planted vs controlled plots. This is correct for all three datasets:

```
Rapideye_data %>%
    group_by(Spp_comp, Spp_richness, Planting) %>%
    summarise(n())
```

```
## # A tibble: 35 x 4
## # Groups:   Spp_comp, Spp_richness [35]
##    Spp_comp                  Spp_richness Planting `n()`
##    <fct>                            <dbl> <fct>    <int>
##  1 0_Unplanted                          0 Control     12
##  2 1_Dipterocarpus_conformis            1 Planted      2
##  3 1_Dryobalanops_lanceolate            1 Planted      2
##  4 1_Hopea_ferruginea                   1 Planted      2
##  5 1_Hopea_sangal                       1 Planted      2
##  6 1_Parashorea_malaanonan              1 Planted      2
##  7 1_Parashorea_tomentalla              1 Planted      2
##  8 1_Shorea_argentifolia                1 Planted      2
##  9 1_Shorea_beccariana                  1 Planted      2
## 10 1_Shorea_faguetiana                  1 Planted      2
## # ... with 25 more rows
```

```
Landsat_data %>%
    group_by(Spp_comp, Spp_richness, Planting) %>%
    summarise(n())
```

```
## # A tibble: 35 x 4
## # Groups:   Spp_comp, Spp_richness [35]
##    Spp_comp                  Spp_richness Planting `n()`
##    <fct>                            <dbl> <fct>    <int>
##  1 0_Unplanted                          0 Control     36
##  2 1_Dipterocarpus_conformis            1 Planted      6
##  3 1_Dryobalanops_lanceolate            1 Planted      6
##  4 1_Hopea_ferruginea                   1 Planted      6
##  5 1_Hopea_sangal                       1 Planted      6
##  6 1_Parashorea_malaanonan              1 Planted      6
##  7 1_Parashorea_tomentalla              1 Planted      6
##  8 1_Shorea_argentifolia                1 Planted      6
##  9 1_Shorea_beccariana                  1 Planted      6
## 10 1_Shorea_faguetiana                  1 Planted      6
## # ... with 25 more rows
```

```
Sat_data %>%
    group_by(Spp_comp, Spp_richness, Planting) %>%
    summarise(n())
```

```
## # A tibble: 35 x 4
## # Groups:   Spp_comp, Spp_richness [35]
##    Spp_comp                  Spp_richness Planting `n()`
##    <fct>                            <dbl> <fct>    <int>
##  1 0_Unplanted                          0 Control     72
##  2 1_Dipterocarpus_conformis            1 Planted     12
##  3 1_Dryobalanops_lanceolate            1 Planted     12
##  4 1_Hopea_ferruginea                   1 Planted     12
##  5 1_Hopea_sangal                       1 Planted     12
##  6 1_Parashorea_malaanonan              1 Planted     12
##  7 1_Parashorea_tomentalla              1 Planted     12
##  8 1_Shorea_argentifolia                1 Planted     12
##  9 1_Shorea_beccariana                  1 Planted     12
## 10 1_Shorea_faguetiana                  1 Planted     12
## # ... with 25 more rows
```

3 Effect of planting treatment

The distribution of our RapidEye data (Fig.1) for each of our five treatments (each level of species richness as well as the 16-species mixture with climber cutting):

```
temp <- RColorBrewer::brewer.pal(5, "Spectral")
temp2 <- c("#a56327", "#dfc27d", "#8dc476", "#80cdc1",
    "#0d8672")

treatment_labels <- c("0", "1", "4", "16", "16*")
bio_label <- expression(paste("Aboveground Biomass (Mg ",
```

```
    Ha^-1, ")"))

Sat_data %>%
    filter(Satellite == "RapidEye") %>%
    ggplot(aes(Treatment, Score)) + geom_violin(aes(fill = Treatment),
    alpha = 0.6) + facet_wrap(~Index, scales = "free",
    strip.position = "left", labeller = as_labeller(c(Biomass = "Aboveground Biomass (Mg ha^-1)",
        LAI = "Leaf Area Index", Cover = "Cover (%)"))) +
    ylab(NULL) + theme(strip.background = element_blank(),
    strip.placement = "outside", strip.text = element_text(size = 11)) +
    geom_jitter(shape = 16, size = 0.9, position = position_jitter(0.1)) +
    scale_fill_manual(values = temp) + scale_x_discrete(labels = treatment_labels) +
    labs(x = "Number of Enrichment Planted Tree Species") +
    theme(legend.position = "none")
```

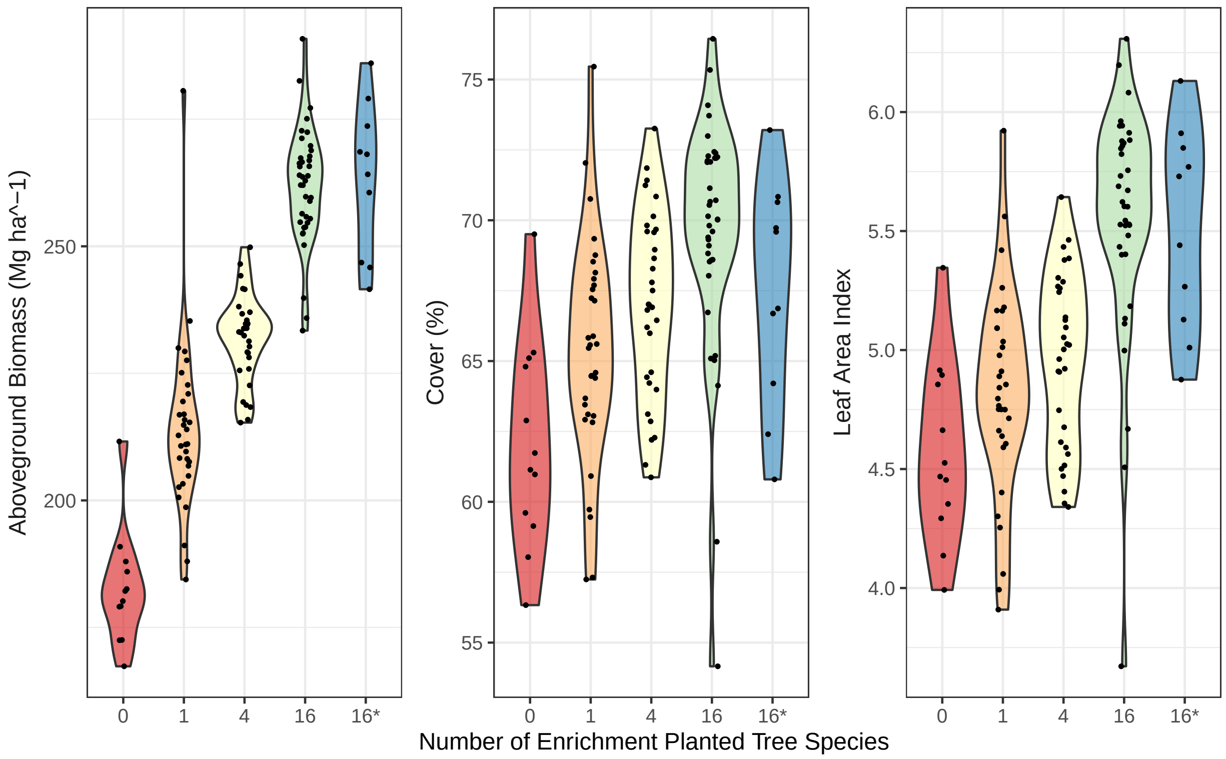

Figure 1: Data points for experimental plots overlaid on violin plots showing (left to right) aboveground biomass, percent vegetation cover, and Leaf Area Index in relation to enrichment planting with seedlings of 0, 1, 4, or 16 species of dipterocarp tree species (16*: enrichment planting with sixteen species plus liana cutting). Note: this figure relates to Fig. 1 (A to C) within the main manuscript.

3.1 Model output

Model used:

$mod\_1 \leftarrow lmer(y \sim Treatment + (1|Block) + (1|Spp\_comp), data = table\_s2\_data)$

Overall we observe an effect of the treatment utilised for each measured index (AGB, Cover, and LAI):

AGB: $F_{4,118.5} = 132.39$, p = < 2.2e-16 LAI: $F_{4,118.1} = 31.84$, p = < 2.2e-16 Cover: $F_{4,118.11} = 14.772$, p = 8.17e-10

```
table_s2_data <- Rapideye_data %>%
    mutate(Treatment = factor(Treatment, levels = c("0_spp",
        "1_spp", "4_spp", "16_spp", "16_cut")))
mod_1_Biomass <- lmer(Biomass ~ Treatment + (1 | Block) +
    (1 | Spp_comp), data = table_s2_data)

anova(mod_1_Biomass, type = "I")
```

```
## Type I Analysis of Variance Table with Satterthwaite's method
##           Sum Sq Mean Sq NumDF DenDF F value    Pr(>F)
## Treatment  83452   20863     4 118.5  132.39 < 2.2e-16
```

```
table_s2_data <- Rapideye_data %>%
    mutate(Treatment = factor(Treatment, levels = c("0_spp",
        "1_spp", "4_spp", "16_spp", "16_cut")))
mod_1_LAI <- lmer(LAI ~ Treatment + (1 | Block) + (1 |
    Spp_comp), data = table_s2_data)

anova(mod_1_LAI, type = "I")
```

```
## Type I Analysis of Variance Table with Satterthwaite's method
##           Sum Sq Mean Sq NumDF DenDF F value    Pr(>F)
## Treatment 17.159  4.2898     4 118.1   31.84 < 2.2e-16
```

```
table_s2_data <- Rapideye_data %>%
    mutate(Treatment = factor(Treatment, levels = c("0_spp",
        "1_spp", "4_spp", "16_spp", "16_cut")))
mod_1_Cover <- lmer(Cover ~ Treatment + (1 | Block) + (1 |
    Spp_comp), data = table_s2_data)

anova(mod_1_Cover, type = "I")
```

```
## Type I Analysis of Variance Table with Satterthwaite's method
##           Sum Sq Mean Sq NumDF  DenDF F value   Pr(>F)
## Treatment 643.62  160.91     4 118.11  14.772 8.17e-10
```

Relevel and return each model to get treatment means and standard error of means and/or confidence interval bounds:

```
table_s2_data <- Rapideye_data %>%
    mutate(Treatment = factor(Treatment, levels = c("0_spp",
        "1_spp", "4_spp", "16_spp", "16_cut")))
mod_1_Biomass <- lmer(Biomass ~ Treatment + (1 | Block) +
    (1 | Spp_comp), data = table_s2_data)
mean_0 <- summary(mod_1_Biomass)$coefficients[1, 1]
se_0 <- summary(mod_1_Biomass)$coefficients[1, 2]
lower_0 <- confint(mod_1_Biomass)[4, 1]
upper_0 <- confint(mod_1_Biomass)[4, 2]
```

```
table_s2_data <- Rapideye_data %>%
    mutate(Treatment = factor(Treatment, levels = c("1_spp",
        "4_spp", "16_spp", "16_cut", "0_spp")))
mod_1_Biomass <- lmer(Biomass ~ Treatment + (1 | Block) +
    (1 | Spp_comp), data = table_s2_data)
mean_1 <- summary(mod_1_Biomass)$coefficients[1, 1]
se_1 <- summary(mod_1_Biomass)$coefficients[1, 2]
lower_1 <- confint(mod_1_Biomass)[4, 1]
upper_1 <- confint(mod_1_Biomass)[4, 2]

table_s2_data <- Rapideye_data %>%
    mutate(Treatment = factor(Treatment, levels = c("4_spp",
        "16_spp", "16_cut", "0_spp", "1_spp")))
mod_1_Biomass <- lmer(Biomass ~ Treatment + (1 | Block) +
    (1 | Spp_comp), data = table_s2_data)
mean_4 <- summary(mod_1_Biomass)$coefficients[1, 1]
se_4 <- summary(mod_1_Biomass)$coefficients[1, 2]
lower_4 <- confint(mod_1_Biomass)[4, 1]
upper_4 <- confint(mod_1_Biomass)[4, 2]

table_s2_data <- Rapideye_data %>%
    mutate(Treatment = factor(Treatment, levels = c("16_spp",
        "16_cut", "0_spp", "1_spp", "4_spp")))
mod_1_Biomass <- lmer(Biomass ~ Treatment + (1 | Block) +
    (1 | Spp_comp), data = table_s2_data)
mean_16_spp <- summary(mod_1_Biomass)$coefficients[1, 1]
se_16_spp <- summary(mod_1_Biomass)$coefficients[1, 2]
lower_16_spp <- confint(mod_1_Biomass)[4, 1]
upper_16_spp <- confint(mod_1_Biomass)[4, 2]

table_s2_data <- Rapideye_data %>%
    mutate(Treatment = factor(Treatment, levels = c("16_cut",
        "16_spp", "0_spp", "1_spp", "4_spp")))
mod_1_Biomass <- lmer(Biomass ~ Treatment + (1 | Block) +
    (1 | Spp_comp), data = table_s2_data)
mean_16_cut <- summary(mod_1_Biomass)$coefficients[1, 1]
se_16_cut <- summary(mod_1_Biomass)$coefficients[1, 2]
lower_16_cut <- confint(mod_1_Biomass)[4, 1]
upper_16_cut <- confint(mod_1_Biomass)[4, 2]

table_s2_Biomass <- tibble(treatment = c("Unplanted", "Monoculture",
    "4 species", "16 species", "16 species with liana removal"),
    Biomass_estimate = c(mean_0, mean_1, mean_4, mean_16_spp,
        mean_16_cut), Biomass_SE = c(se_0, se_1, se_4,
        se_16_spp, se_16_cut), Biomass_lower = c(lower_0,
        lower_1, lower_4, lower_16_spp, lower_16_cut),
    Biomass_upper = c(upper_0, upper_1, upper_4, upper_16_spp,
        upper_16_cut))

table_s2_data <- Rapideye_data %>%
    mutate(Treatment = factor(Treatment, levels = c("0_spp",
        "1_spp", "4_spp", "16_spp", "16_cut")))
mod_1_LAI <- lmer(LAI ~ Treatment + (1 | Block) + (1 |
```

```
    Spp_comp), data = table_s2_data)
confint(mod_1_LAI)[4, 1]
```

```
## [1] 3.986466
```

```
confint(mod_1_LAI)[4, 2]
```

```
## [1] 5.163051
```

```
mean_0 <- summary(mod_1_LAI)$coefficients[1, 1]
se_0 <- summary(mod_1_LAI)$coefficients[1, 2]
lower_0 <- confint(mod_1_LAI)[4, 1]
upper_0 <- confint(mod_1_LAI)[4, 2]


table_s2_data <- Rapideye_data %>%
    mutate(Treatment = factor(Treatment, levels = c("1_spp",
        "4_spp", "16_spp", "16_cut", "0_spp")))
mod_1_LAI <- lmer(LAI ~ Treatment + (1 | Block) + (1 |
    Spp_comp), data = table_s2_data)
mean_1 <- summary(mod_1_LAI)$coefficients[1, 1]
se_1 <- summary(mod_1_LAI)$coefficients[1, 2]
lower_1 <- confint(mod_1_LAI)[4, 1]
upper_1 <- confint(mod_1_LAI)[4, 2]

table_s2_data <- Rapideye_data %>%
    mutate(Treatment = factor(Treatment, levels = c("4_spp",
        "16_spp", "16_cut", "0_spp", "1_spp")))
mod_1_LAI <- lmer(LAI ~ Treatment + (1 | Block) + (1 |
    Spp_comp), data = table_s2_data)
mean_4 <- summary(mod_1_LAI)$coefficients[1, 1]
se_4 <- summary(mod_1_LAI)$coefficients[1, 2]
lower_4 <- confint(mod_1_LAI)[4, 1]
upper_4 <- confint(mod_1_LAI)[4, 2]

table_s2_data <- Rapideye_data %>%
    mutate(Treatment = factor(Treatment, levels = c("16_spp",
        "16_cut", "0_spp", "1_spp", "4_spp")))
mod_1_LAI <- lmer(LAI ~ Treatment + (1 | Block) + (1 |
    Spp_comp), data = table_s2_data)
mean_16_spp <- summary(mod_1_LAI)$coefficients[1, 1]
se_16_spp <- summary(mod_1_LAI)$coefficients[1, 2]
lower_16_spp <- confint(mod_1_LAI)[4, 1]
upper_16_spp <- confint(mod_1_LAI)[4, 2]

table_s2_data <- Rapideye_data %>%
    mutate(Treatment = factor(Treatment, levels = c("16_cut",
        "0_spp", "1_spp", "4_spp", "16_spp")))
mod_1_LAI <- lmer(LAI ~ Treatment + (1 | Block) + (1 |
    Spp_comp), data = table_s2_data)
mean_16_cut <- summary(mod_1_LAI)$coefficients[1, 1]
se_16_cut <- summary(mod_1_LAI)$coefficients[1, 2]
```

```
lower_16_cut <- confint(mod_1_LAI)[4, 1]
upper_16_cut <- confint(mod_1_LAI)[4, 2]

table_s2_LAI <- tibble(treatment = c("Unplanted", "Monoculture",
    "4 species", "16 species", "16 species with liana removal"),
    LAI_estimate = c(mean_0, mean_1, mean_4, mean_16_spp,
        mean_16_cut), LAI_SE = c(se_0, se_1, se_4, se_16_spp,
        se_16_cut), LAI_lower = c(lower_0, lower_1, lower_4,
        lower_16_spp, lower_16_cut), LAI_upper = c(upper_0,
        upper_1, upper_4, upper_16_spp, upper_16_cut))


table_s2_data <- Rapideye_data %>%
    mutate(Treatment = factor(Treatment, levels = c("0_spp",
        "1_spp", "4_spp", "16_spp", "16_cut")))
mod_1_Cover <- lmer(Cover ~ Treatment + (1 | Block) + (1 |
    Spp_comp), data = table_s2_data)
confint(mod_1_Cover)[4, 1]
```

```
## [1] 56.81364
```

```
confint(mod_1_Cover)[4, 2]
```

```
## [1] 67.27936
```

```
mean_0 <- summary(mod_1_Cover)$coefficients[1, 1]
se_0 <- summary(mod_1_Cover)$coefficients[1, 2]
lower_0 <- confint(mod_1_Cover)[4, 1]
upper_0 <- confint(mod_1_Cover)[4, 2]


table_s2_data <- Rapideye_data %>%
    mutate(Treatment = factor(Treatment, levels = c("1_spp",
        "4_spp", "16_spp", "16_cut", "0_spp")))
mod_1_Cover <- lmer(Cover ~ Treatment + (1 | Block) + (1 |
    Spp_comp), data = table_s2_data)
mean_1 <- summary(mod_1_Cover)$coefficients[1, 1]
se_1 <- summary(mod_1_Cover)$coefficients[1, 2]
lower_1 <- confint(mod_1_Cover)[4, 1]
upper_1 <- confint(mod_1_Cover)[4, 2]

table_s2_data <- Rapideye_data %>%
    mutate(Treatment = factor(Treatment, levels = c("4_spp",
        "16_spp", "16_cut", "0_spp", "1_spp")))
mod_1_Cover <- lmer(Cover ~ Treatment + (1 | Block) + (1 |
    Spp_comp), data = table_s2_data)
mean_4 <- summary(mod_1_Cover)$coefficients[1, 1]
se_4 <- summary(mod_1_Cover)$coefficients[1, 2]
lower_4 <- confint(mod_1_Cover)[4, 1]
upper_4 <- confint(mod_1_Cover)[4, 2]

table_s2_data <- Rapideye_data %>%
```

```r
    mutate(Treatment = factor(Treatment, levels = c("16_spp",
        "16_cut", "0_spp", "1_spp", "4_spp")))
mod_1_Cover <- lmer(Cover ~ Treatment + (1 | Block) + (1 |
    Spp_comp), data = table_s2_data)
mean_16_spp <- summary(mod_1_Cover)$coefficients[1, 1]
se_16_spp <- summary(mod_1_Cover)$coefficients[1, 2]
lower_16_spp <- confint(mod_1_Cover)[4, 1]
upper_16_spp <- confint(mod_1_Cover)[4, 2]

table_s2_data <- Rapideye_data %>%
    mutate(Treatment = factor(Treatment, levels = c("16_cut",
        "0_spp", "1_spp", "4_spp", "16_spp")))
mod_1_Cover <- lmer(Cover ~ Treatment + (1 | Block) + (1 |
    Spp_comp), data = table_s2_data)
mean_16_cut <- summary(mod_1_Cover)$coefficients[1, 1]
se_16_cut <- summary(mod_1_Cover)$coefficients[1, 2]
lower_16_cut <- confint(mod_1_Cover)[4, 1]
upper_16_cut <- confint(mod_1_Cover)[4, 2]

table_s2_Cover <- tibble(treatment = c("Unplanted", "Monoculture",
    "4 species", "16 species", "16 species with liana removal"),
    Cover_estimate = c(mean_0, mean_1, mean_4, mean_16_spp,
        mean_16_cut), Cover_SE = c(se_0, se_1, se_4, se_16_spp,
        se_16_cut), Cover_lower = c(lower_0, lower_1, lower_4,
        lower_16_spp, lower_16_cut), Cover_upper = c(upper_0,
        upper_1, upper_4, upper_16_spp, upper_16_cut))

table_s2_Cover
```

```
## # A tibble: 5 x 5
##   treatment                     Cover_estimate Cover_SE Cover_lower Cover_upper
##   <chr>                                  <dbl>    <dbl>       <dbl>       <dbl>
## 1 Unplanted                               62.0     2.30        56.8        67.3
## 2 Monoculture                             65.3     2.17        60.2        70.5
## 3 4 species                               67.0     2.17        61.9        72.2
## 4 16 species                              69.3     2.16        64.1        74.4
## 5 16 species with liana removal           69.6     2.36        64.3        74.9
```

Combining each output into a single table (table S2):

```r
table_s2 <- table_s2_Biomass %>%
    merge(table_s2_LAI, by = "treatment") %>%
    merge(table_s2_Cover, by = "treatment")
table_s2
```

```
##                       treatment Biomass_estimate Biomass_SE Biomass_lower
## 1                    16 species         261.5512   3.252924      254.5086
## 2 16 species with liana removal         265.7326   4.827987      256.7029
## 3                     4 species         231.8966   3.366087      224.8100
## 4                   Monoculture         213.8921   3.366087      206.8055
## 5                     Unplanted         182.6662   4.420203      174.4684
##   Biomass_upper LAI_estimate    LAI_SE LAI_lower LAI_upper Cover_estimate
```

```
## 1      268.4878     5.521419 0.2431198  4.943732  6.097975       69.26514
## 2      274.6759     5.744273 0.2649006  5.205906  6.286233       69.56770
## 3      238.9831     4.956316 0.2444237  4.378267  5.534358       67.02580
## 4      220.9785     4.822589 0.2444237  4.244540  5.400631       65.32841
## 5      190.8638     4.574765 0.2583798  3.986466  5.163051       62.04656
##   Cover_SE Cover_lower Cover_upper
## 1 2.162387    64.12876    74.39124
## 2 2.360185    64.32015    74.87814
## 3 2.174239    61.88613    72.16542
## 4 2.174239    60.18874    70.46803
## 5 2.301010    56.81364    67.27936
```

4 Effect of planting

To compare the effect of planting diversity (i.e. 1, 4, or 16 species plot mixtures) we aggregate data into planted vs unplanted plots. This creates unequal sample sizes (12 unplanted vs 112 planted plots), but this was not the primary purpose of this experimental design.

4.1 Modelling

Model used:

$$mod\_2 \leftarrow lmer(y \sim Planting + (1|Block) + (1|Spp\_comp), data = table\_s3\_data)$$

Creating a table of planted vs unplanted estimates for AGB, LAI, and Cover (table S3):

```
table_s3_data <- Rapideye_data %>%
    mutate(Spp_richness = factor(Spp_richness, levels = c("1",
        "4", "16", "0")))
mod_2_Biomass <- lmer(Biomass ~ Planting + (1 | Block) +
    (1 | Spp_comp), data = table_s3_data)
mean_unplanted <- summary(mod_2_Biomass)$coefficients[1,
    1]
se_unplanted <- summary(mod_2_Biomass)$coefficients[1,
    2]
lower_unplanted <- confint(mod_2_Biomass)[4, 1]
upper_unplanted <- confint(mod_2_Biomass)[4, 2]

mean_difference <- summary(mod_2_Biomass)$coefficients[2,
    1]
se_difference <- summary(mod_2_Biomass)$coefficients[2,
    2]
lower_difference <- confint(mod_2_Biomass)[5, 1]
upper_difference <- confint(mod_2_Biomass)[5, 2]


table_s3_data <- Rapideye_data %>%
    mutate(Spp_richness = factor(Spp_richness, levels = c("1",
        "4", "16", "0"))) %>%
    mutate(Planting = factor(Planting, levels = c("Planted",
        "Control")))
```

```
mod_2_Biomass <- lmer(Biomass ~ Planting + (1 | Block) +
    (1 | Spp_comp), data = table_s3_data)
mean_planted <- summary(mod_2_Biomass)$coefficients[1,
    1]
se_planted <- summary(mod_2_Biomass)$coefficients[1, 2]
lower_planted <- confint(mod_2_Biomass)[4, 1]
upper_planted <- confint(mod_2_Biomass)[4, 2]

table_s3_Biomass <- tibble(treatment = c("Unplanted", "Planted",
    "Difference"), Biomass_estimate = c(mean_unplanted,
    mean_planted, mean_difference), Biomass_SE = c(se_unplanted,
    se_planted, se_difference), Biomass_lower = c(lower_unplanted,
    lower_planted, lower_difference), Biomass_upper = c(upper_unplanted,
    upper_planted, upper_difference))




table_s3_data <- Rapideye_data %>%
    mutate(Spp_richness = factor(Spp_richness, levels = c("1",
        "4", "16", "0")))
mod_2_LAI <- lmer(LAI ~ Planting + (1 | Block) + (1 | Spp_comp),
    data = table_s3_data)
mean_unplanted <- summary(mod_2_LAI)$coefficients[1, 1]
se_unplanted <- summary(mod_2_LAI)$coefficients[1, 2]
lower_unplanted <- confint(mod_2_LAI)[4, 1]
upper_unplanted <- confint(mod_2_LAI)[4, 2]

mean_difference <- summary(mod_2_LAI)$coefficients[2, 1]
se_difference <- summary(mod_2_LAI)$coefficients[2, 2]
lower_difference <- confint(mod_2_LAI)[5, 1]
upper_difference <- confint(mod_2_LAI)[5, 2]


table_s3_data <- Rapideye_data %>%
    mutate(Spp_richness = factor(Spp_richness, levels = c("1",
        "4", "16", "0"))) %>%
    mutate(Planting = factor(Planting, levels = c("Planted",
        "Control")))
mod_2_LAI <- lmer(LAI ~ Planting + (1 | Block) + (1 | Spp_comp),
    data = table_s3_data)
mean_planted <- summary(mod_2_LAI)$coefficients[1, 1]
se_planted <- summary(mod_2_LAI)$coefficients[1, 2]
lower_planted <- confint(mod_2_LAI)[4, 1]
upper_planted <- confint(mod_2_LAI)[4, 2]

table_s3_LAI <- tibble(treatment = c("Unplanted", "Planted",
    "Difference"), LAI_estimate = c(mean_unplanted, mean_planted,
    mean_difference), LAI_SE = c(se_unplanted, se_planted,
    se_difference), LAI_lower = c(lower_unplanted, lower_planted,
    lower_difference), LAI_upper = c(upper_unplanted, upper_planted,
    upper_difference))
```

```r
table_s3_data <- Rapideye_data %>%
    mutate(Spp_richness = factor(Spp_richness, levels = c("1",
        "4", "16", "0")))
mod_2_Cover <- lmer(Cover ~ Planting + (1 | Block) + (1 |
    Spp_comp), data = table_s3_data)
mean_unplanted <- summary(mod_2_Cover)$coefficients[1,
    1]
se_unplanted <- summary(mod_2_Cover)$coefficients[1, 2]
lower_unplanted <- confint(mod_2_Cover)[4, 1]
upper_unplanted <- confint(mod_2_Cover)[4, 2]

mean_difference <- summary(mod_2_Cover)$coefficients[2,
    1]
se_difference <- summary(mod_2_Cover)$coefficients[2, 2]
lower_difference <- confint(mod_2_Cover)[5, 1]
upper_difference <- confint(mod_2_Cover)[5, 2]


table_s3_data <- Rapideye_data %>%
    mutate(Spp_richness = factor(Spp_richness, levels = c("1",
        "4", "16", "0"))) %>%
    mutate(Planting = factor(Planting, levels = c("Planted",
        "Control")))
mod_2_Cover <- lmer(Cover ~ Planting + (1 | Block) + (1 |
    Spp_comp), data = table_s3_data)
mean_planted <- summary(mod_2_Cover)$coefficients[1, 1]
se_planted <- summary(mod_2_Cover)$coefficients[1, 2]
lower_planted <- confint(mod_2_Cover)[4, 1]
upper_planted <- confint(mod_2_Cover)[4, 2]

table_s3_Cover <- tibble(treatment = c("Unplanted", "Planted",
    "Difference"), Cover_estimate = c(mean_unplanted, mean_planted,
    mean_difference), Cover_SE = c(se_unplanted, se_planted,
    se_difference), Cover_lower = c(lower_unplanted, lower_planted,
    lower_difference), Cover_upper = c(upper_unplanted,
    upper_planted, upper_difference))


table_s3 <- table_s3_Biomass %>%
    merge(table_s3_LAI, by = "treatment") %>%
    merge(table_s3_Cover, by = "treatment")
```

Here significant differences between planted and unplanted plots' AGB and Cover estimates were identified, and a positive but non-significant difference between planted and unplanted plot's LAI estimates.

4.2 Graphing

```r
temp1 <- table_s3 %>%
    select(treatment, contains("estimate")) %>%
```

```
    pivot_longer(contains("estimate"), names_to = "index",
        values_to = "estimate") %>%
    mutate(index = case_when(str_detect(index, "Biomass") ~
        "atop(Aboveground~Biomass,(Mg~ha^{-1}))", str_detect(index,
        "Cover") ~ "Vegetation~cover~('%')", str_detect(index,
        "LAI") ~ "Leaf~Area~Index"))

temp2 <- table_s3 %>%
    select(treatment, contains("upper")) %>%
    pivot_longer(contains("upper"), names_to = "index",
        values_to = "upper_limit") %>%
    mutate(index = case_when(str_detect(index, "Biomass") ~
        "atop(Aboveground~Biomass,(Mg~ha^{-1}))", str_detect(index,
        "Cover") ~ "Vegetation~cover~('%')", str_detect(index,
        "LAI") ~ "Leaf~Area~Index"))

temp3 <- table_s3 %>%
    select(treatment, contains("lower")) %>%
    pivot_longer(contains("lower"), names_to = "index",
        values_to = "lower_limit") %>%
    mutate(index = case_when(str_detect(index, "Biomass") ~
        "atop(Aboveground~Biomass,(Mg~ha^{-1}))", str_detect(index,
        "Cover") ~ "Vegetation~cover~('%')", str_detect(index,
        "LAI") ~ "Leaf~Area~Index"))

fig1def_data <- merge(temp1, temp2, by = c("treatment",
    "index")) %>%
    merge(temp3, by = c("treatment", "index")) %>%
    mutate(treatment = ifelse(treatment == "Unplanted",
        "Control", treatment)) %>%
    mutate(index = factor(index, levels = c("atop(Aboveground~Biomass,(Mg~ha^{-1}))",
        "Vegetation~cover~('%')", "Leaf~Area~Index"))) %>%
    filter(treatment != "Difference")
fig1def_data
```

```
##   treatment                                  index   estimate upper_limit
## 1   Planted atop(Aboveground~Biomass,(Mg~ha^{-1})) 225.991906  234.550476
## 2   Planted                        Leaf~Area~Index   4.963516    5.529689
## 3   Planted                 Vegetation~cover~('%')  66.672774   71.683539
## 4   Control atop(Aboveground~Biomass,(Mg~ha^{-1})) 182.666151  212.270023
## 5   Control                        Leaf~Area~Index   4.574765    5.260443
## 6   Control                 Vegetation~cover~('%')  62.046556   67.465192
##   lower_limit
## 1  217.497023
## 2    4.400213
## 3   61.712060
## 4  153.062280
## 5    3.889104
## 6   56.627978
```

```
fig_1def <- ggplot(fig1def_data) + geom_pointrange(aes(x = treatment,
    y = estimate, ymin = lower_limit, ymax = upper_limit,
    colour = treatment), size = 1, fill = "white", shape = 22) +
```

```
    facet_wrap(~index, scales = "free_y", strip.position = "left",
        labeller = label_parsed) + scale_colour_manual(values = c(cols[1],
    cols[2])) + theme(legend.position = "none") + labs(x = "Replanting treatment",
    y = NULL) + theme(strip.background = element_blank(),
    strip.placement = "outside", strip.text = element_text(size = 11),
    panel.spacing = unit(0, "lines"))

fig_1def
```

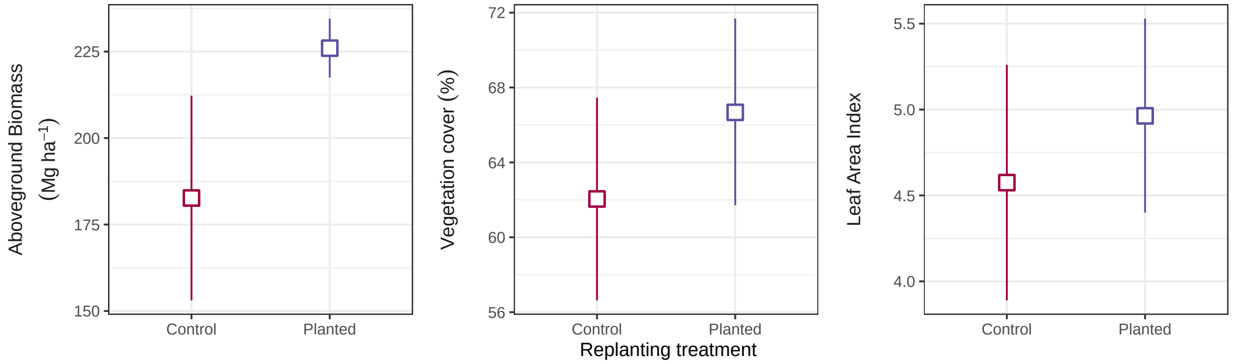

5 Effect of planted species richness

5.1 Modelling

Model used:

$$mod\_3 \leftarrow lmer(y \sim log2(Spp\_richness) + factor(Spp\_richness) + Treatment + (1|Block) + (1|Spp\_comp), subset = Spp\_ri$$ [illegible]

This model gives us estimates for the change in AGB, LAI, or Cover for a doubling in species richness (table S4):

```
table_s4_data <- Rapideye_data
mod_3_Biomass <- lmer(Biomass ~ log2(Spp_richness) + factor(Spp_richness) +
    Treatment + (1 | Block) + (1 | Spp_comp), subset = Spp_richness >
    0, data = table_s4_data)

slope_estimate_Biomass <- summary(mod_3_Biomass)$coefficients[2,
    1]
slope_se_Biomass <- summary(mod_3_Biomass)$coefficients[2,
    2]
slope_lower_Biomass <- confint(mod_3_Biomass)[5, 1]
slope_upper_Biomass <- confint(mod_3_Biomass)[5, 2]



table_s4_data <- Rapideye_data
mod_3_LAI <- lmer(LAI ~ log2(Spp_richness) + factor(Spp_richness) +
```

```
    Treatment + (1 | Block) + (1 | Spp_comp), subset = Spp_richness >
    0, data = table_s4_data)

slope_estimate_LAI <- summary(mod_3_LAI)$coefficients[2,
    1]
slope_se_LAI <- summary(mod_3_LAI)$coefficients[2, 2]
slope_lower_LAI <- confint(mod_3_LAI)[5, 1]
slope_upper_LAI <- confint(mod_3_LAI)[5, 2]



table_s4_data <- Rapideye_data
mod_3_Cover <- lmer(Cover ~ log2(Spp_richness) + factor(Spp_richness) +
    Treatment + (1 | Block) + (1 | Spp_comp), subset = Spp_richness >
    0, data = table_s4_data)

slope_estimate_Cover <- summary(mod_3_Cover)$coefficients[2,
    1]
slope_se_Cover <- summary(mod_3_Cover)$coefficients[2,
    2]
slope_lower_Cover <- confint(mod_3_Cover)[5, 1]
slope_upper_Cover <- confint(mod_3_Cover)[5, 2]



table_s4 <- tibble(index = c("Biomass", "LAI", "Cover"),
    slope_estimate = c(slope_estimate_Biomass, slope_estimate_LAI,
        slope_estimate_Cover), slope_se = c(slope_se_Biomass,
        slope_se_LAI, slope_se_Cover), slope_lower = c(slope_lower_Biomass,
        slope_lower_LAI, slope_lower_Cover), slope_upper = c(slope_upper_Biomass,
        slope_upper_LAI, slope_upper_Cover))

table_s4
```

```
## # A tibble: 3 x 5
##   index   slope_estimate slope_se slope_lower slope_upper
##   <chr>            <dbl>    <dbl>       <dbl>       <dbl>
## 1 Biomass         12.9     1.19        10.3        15.1
## 2 LAI              0.231   0.0349       0.161       0.298
## 3 Cover            1.06    0.313        0.436       1.66
```

This shows a significant effect of planting for each of the three measured indices.

5.2 Graphing

A slightly modified version of model 3 for visualization purposes that omits species richness in order to get a single intercept for the log-2 effect of species richness. This model is used for visualization of the species richness effect, model 3 is a more complete version preferred for inference.

```
table_s4_2_data <- Rapideye_data
mod_3b_Biomass <- lmer(Biomass ~ log2(Spp_richness) + (1 |
    Block) + (1 | Spp_comp), subset = Spp_richness > 0,
```

```
    data = table_s4_2_data)

intercept_estimate_Biomass <- summary(mod_3b_Biomass)$coefficients[1,
    1]
intercept_se_Biomass <- summary(mod_3b_Biomass)$coefficients[1,
    2]
slope_estimate_Biomass <- summary(mod_3b_Biomass)$coefficients[2,
    1]
slope_se_Biomass <- summary(mod_3b_Biomass)$coefficients[2,
    2]
intercept_lower_Biomass <- confint(mod_3b_Biomass)[4, 1]
intercept_upper_Biomass <- confint(mod_3b_Biomass)[4, 2]
slope_lower_Biomass <- confint(mod_3b_Biomass)[5, 1]
slope_upper_Biomass <- confint(mod_3b_Biomass)[5, 2]



table_s4_2_data <- Rapideye_data
mod_3b_LAI <- lmer(LAI ~ log2(Spp_richness) + (1 | Block) +
    (1 | Spp_comp), subset = Spp_richness > 0, data = table_s4_2_data)

intercept_estimate_LAI <- summary(mod_3b_LAI)$coefficients[1,
    1]
intercept_se_LAI <- summary(mod_3b_LAI)$coefficients[1,
    2]
slope_estimate_LAI <- summary(mod_3b_LAI)$coefficients[2,
    1]
slope_se_LAI <- summary(mod_3b_LAI)$coefficients[2, 2]
intercept_lower_LAI <- confint(mod_3b_LAI)[4, 1]
intercept_upper_LAI <- confint(mod_3b_LAI)[4, 2]
slope_lower_LAI <- confint(mod_3b_LAI)[5, 1]
slope_upper_LAI <- confint(mod_3b_LAI)[5, 2]



table_s4_2_data <- Rapideye_data
mod_3b_Cover <- lmer(Cover ~ log2(Spp_richness) + (1 |
    Block) + (1 | Spp_comp), subset = Spp_richness > 0,
    data = table_s4_2_data)

intercept_estimate_Cover <- summary(mod_3b_Cover)$coefficients[1,
    1]
intercept_se_Cover <- summary(mod_3b_Cover)$coefficients[1,
    2]
slope_estimate_Cover <- summary(mod_3b_Cover)$coefficients[2,
    1]
slope_se_Cover <- summary(mod_3b_Cover)$coefficients[2,
    2]
intercept_lower_Cover <- confint(mod_3b_Cover)[4, 1]
intercept_upper_Cover <- confint(mod_3b_Cover)[4, 2]
slope_lower_Cover <- confint(mod_3b_Cover)[5, 1]
slope_upper_Cover <- confint(mod_3b_Cover)[5, 2]
```

```
table_s4_2 <- tibble(index = c("Biomass", "LAI", "Cover"),
    intercept_estimate = c(intercept_estimate_Biomass,
        intercept_estimate_LAI, intercept_estimate_Cover),
    intercept_se = c(intercept_se_Biomass, intercept_se_LAI,
        intercept_se_Cover), intercept_lower = c(intercept_lower_Biomass,
        intercept_lower_LAI, intercept_lower_Cover), intercept_upper = c(intercept_upper_Biomass,
        intercept_upper_LAI, intercept_upper_Cover), slope_estimate = c(slope_estimate_Biomass,
        slope_estimate_LAI, slope_estimate_Cover), slope_se = c(slope_se_Biomass,
        slope_se_LAI, slope_se_Cover), slope_lower = c(slope_lower_Biomass,
        slope_lower_LAI, slope_lower_Cover), slope_upper = c(slope_upper_Biomass,
        slope_upper_LAI, slope_upper_Cover))
```

```
fig_2a_data_intercept <- table_s4_2 %>%
    filter(index == "Biomass")
fig_2a_data_slope <- table_s4 %>%
    filter(index == "Biomass")
```

This table is used to generate fig 2a, S2, and S3:

```
# width = 400, height = 320
fig_2a <- Rapideye_data %>%
    filter(Spp_richness > 0) %>%
    ggplot(aes(Spp_richness, Biomass)) + geom_jitter(width = 0.07,
    height = 0, size = 1) + geom_abline(slope = fig_2a_data_slope[[1,
    2]], intercept = fig_2a_data_intercept[[1, 2]], colour = "blue") +
    labs(x = "Number of enrichment planted tree species",
        y = expression(paste("Aboveground biomass (Mg  ",
            Ha^-1, ")"))) + scale_x_continuous(trans = log2_trans()) +
    scale_y_continuous(sec.axis = sec_axis(~100 * ./(fig_2a_data_intercept[[1,
        2]]), name = "Percent biomass relative\nto single species mixtures"))

fig_2a
```

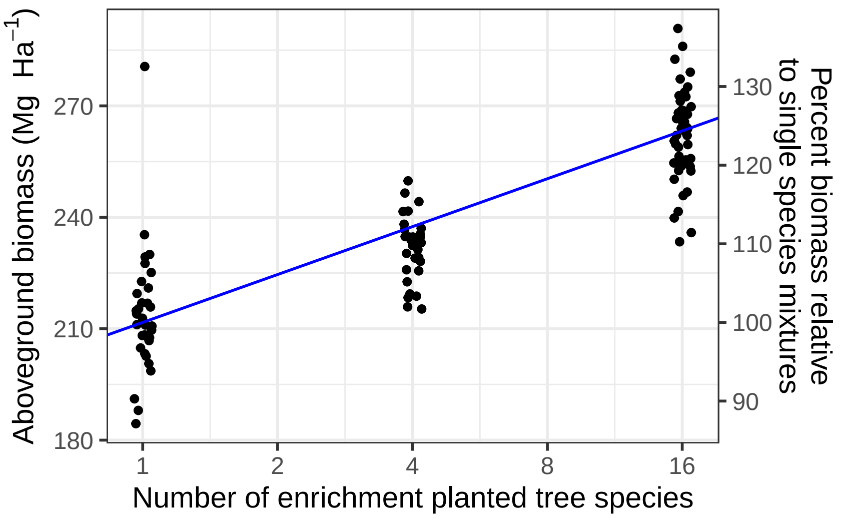

```
fig_s2_data_intercept <- table_s4_2 %>%
    filter(index == "Cover")
fig_s2_data_slope <- table_s4 %>%
    filter(index == "Cover")
```

```
# width = 450, height = 320
fig_s2 <- Rapideye_data %>%
    filter(Spp_richness > 0) %>%
    ggplot(aes(Spp_richness, Cover)) + geom_jitter(width = 0.07,
    height = 0, size = 1) + geom_abline(slope = fig_s2_data_slope[[1,
    2]], intercept = fig_s2_data_intercept[[1, 2]], colour = "blue") +
    labs(x = "Number of enrichment planted tree species",
        y = "Vegetation cover (%)") + scale_x_continuous(trans = log2_trans()) +
    scale_y_continuous(sec.axis = sec_axis(~100 * ./(fig_s2_data_intercept[[1,
        2]]), name = "Percent cover relative\nto single species mixtures"))

fig_s2
```

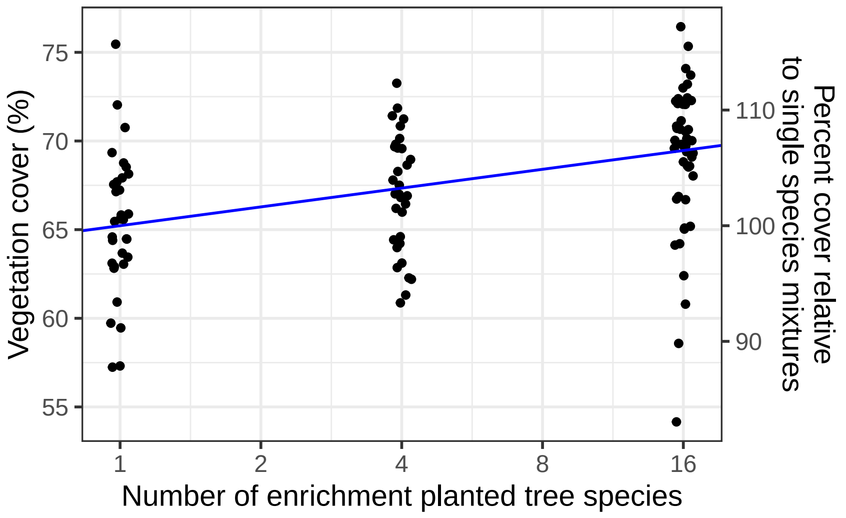


```
fig_s3_data_intercept <- table_s4_2 %>%
    filter(index == "LAI")
fig_s3_data_slope <- table_s4 %>%
    filter(index == "LAI")
```

```
# width = 450, height = 320
fig_s3 <- Rapideye_data %>%
    filter(Spp_richness > 0) %>%
    ggplot(aes(Spp_richness, LAI)) + geom_jitter(width = 0.07,
    height = 0, size = 1) + geom_abline(slope = fig_s3_data_slope[[1,
    2]], intercept = fig_s3_data_intercept[[1, 2]], colour = "blue") +
    labs(x = "Number of enrichment planted tree species",
        y = "Leaf Area Index") + scale_x_continuous(trans = log2_trans()) +
    scale_y_continuous(sec.axis = sec_axis(~100 * ./(fig_s3_data_intercept[[1,
        2]]), name = "Percent LAI relative\nto single species mixtures"))

fig_s3
```

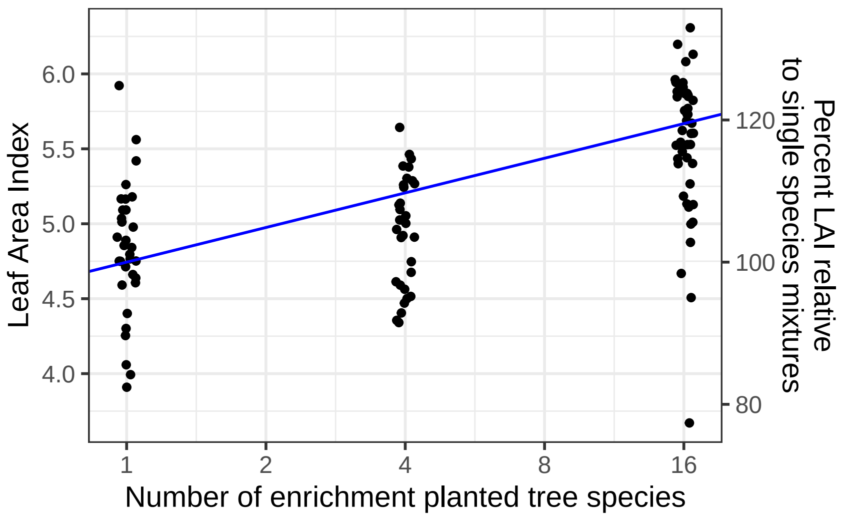

6 Change in response to planted species richness over time

To obtain an estimate for the response to species richness over time, we can make use of the three time periods that are contained with the Landsat data. This data only includes values for canopy cover.

6.1 Modelling

Here individual estimates for each treatment group at each time period are estimated, rather than treating time and species richness as continuous variates.

Model used:

$$mod\_4 \leftarrow lmer(Cover \sim factor(Year)*factor(Spp\_richness)+(1|Block)+(1|Spp\_comp)+(1|Plot), data = table\_s5\_data$$

```
table_s5_data <- Landsat_data %>%
    mutate(Spp_richness = factor(Spp_richness, levels = c("0",
        "1", "4", "16")), Year = factor(Year, levels = c("2000",
        "2005", "2010")))


mod_4 <- lmer(Cover ~ factor(Year) * factor(Spp_richness) +
    (1 | Block) + (1 | Spp_comp) + (1 | Plot), data = table_s5_data)

# summary(mod_4)$coefficients

mean_2000_0 <- summary(mod_4)$coefficients[1, 1]
se_2000_0 <- summary(mod_4)$coefficients[1, 2]
lower_2000_0 <- confint(mod_4)[5, 1]
upper_2000_0 <- confint(mod_4)[5, 2]

table_s5_data <- Landsat_data %>%
    mutate(Spp_richness = factor(Spp_richness, levels = c("1",
        "4", "16", "0")), Year = factor(Year, levels = c("2000",
```

```r
        "2005", "2010")))


mod_4 <- lmer(Cover ~ factor(Year) * factor(Spp_richness) +
    (1 | Block) + (1 | Spp_comp) + (1 | Plot), data = table_s5_data)

# summary(mod_4)$coefficients

mean_2000_1 <- summary(mod_4)$coefficients[1, 1]
se_2000_1 <- summary(mod_4)$coefficients[1, 2]
lower_2000_1 <- confint(mod_4)[5, 1]
upper_2000_1 <- confint(mod_4)[5, 2]


table_s5_data <- Landsat_data %>%
    mutate(Spp_richness = factor(Spp_richness, levels = c("4",
        "16", "0", "1")), Year = factor(Year, levels = c("2000",
        "2005", "2010")))


mod_4 <- lmer(Cover ~ factor(Year) * factor(Spp_richness) +
    (1 | Block) + (1 | Spp_comp) + (1 | Plot), data = table_s5_data)

# summary(mod_4)$coefficients

mean_2000_4 <- summary(mod_4)$coefficients[1, 1]
se_2000_4 <- summary(mod_4)$coefficients[1, 2]
lower_2000_4 <- confint(mod_4)[5, 1]
upper_2000_4 <- confint(mod_4)[5, 2]



table_s5_data <- Landsat_data %>%
    mutate(Spp_richness = factor(Spp_richness, levels = c("16",
        "0", "1", "4")), Year = factor(Year, levels = c("2000",
        "2005", "2010")))


mod_4 <- lmer(Cover ~ factor(Year) * factor(Spp_richness) +
    (1 | Block) + (1 | Spp_comp) + (1 | Plot), data = table_s5_data)

# summary(mod_4)$coefficients

mean_2000_16 <- summary(mod_4)$coefficients[1, 1]
se_2000_16 <- summary(mod_4)$coefficients[1, 2]
lower_2000_16 <- confint(mod_4)[5, 1]
upper_2000_16 <- confint(mod_4)[5, 2]

## 2005

table_s5_data <- Landsat_data %>%
    mutate(Spp_richness = factor(Spp_richness, levels = c("0",
        "1", "4", "16")), Year = factor(Year, levels = c("2005",
```

```
        "2010", "2000")))


mod_4 <- lmer(Cover ~ factor(Year) * factor(Spp_richness) +
    (1 | Block) + (1 | Spp_comp) + (1 | Plot), data = table_s5_data)

# summary(mod_4)$coefficients

mean_2005_0 <- summary(mod_4)$coefficients[1, 1]
se_2005_0 <- summary(mod_4)$coefficients[1, 2]
lower_2005_0 <- confint(mod_4)[5, 1]
upper_2005_0 <- confint(mod_4)[5, 2]


table_s5_data <- Landsat_data %>%
    mutate(Spp_richness = factor(Spp_richness, levels = c("1",
        "4", "16", "0")), Year = factor(Year, levels = c("2005",
        "2010", "2000")))


mod_4 <- lmer(Cover ~ factor(Year) * factor(Spp_richness) +
    (1 | Block) + (1 | Spp_comp) + (1 | Plot), data = table_s5_data)

# summary(mod_4)$coefficients

mean_2005_1 <- summary(mod_4)$coefficients[1, 1]
se_2005_1 <- summary(mod_4)$coefficients[1, 2]
lower_2005_1 <- confint(mod_4)[5, 1]
upper_2005_1 <- confint(mod_4)[5, 2]

table_s5_data <- Landsat_data %>%
    mutate(Spp_richness = factor(Spp_richness, levels = c("4",
        "16", "0", "1")), Year = factor(Year, levels = c("2005",
        "2010", "2000")))


mod_4 <- lmer(Cover ~ factor(Year) * factor(Spp_richness) +
    (1 | Block) + (1 | Spp_comp) + (1 | Plot), data = table_s5_data)

# summary(mod_4)$coefficients

mean_2005_4 <- summary(mod_4)$coefficients[1, 1]
se_2005_4 <- summary(mod_4)$coefficients[1, 2]
lower_2005_4 <- confint(mod_4)[5, 1]
upper_2005_4 <- confint(mod_4)[5, 2]


table_s5_data <- Landsat_data %>%
    mutate(Spp_richness = factor(Spp_richness, levels = c("16",
        "0", "1", "4")), Year = factor(Year, levels = c("2005",
        "2010", "2000")))
```

```r
mod_4 <- lmer(Cover ~ factor(Year) * factor(Spp_richness) +
    (1 | Block) + (1 | Spp_comp) + (1 | Plot), data = table_s5_data)

# summary(mod_4)$coefficients

mean_2005_16 <- summary(mod_4)$coefficients[1, 1]
se_2005_16 <- summary(mod_4)$coefficients[1, 2]
lower_2005_16 <- confint(mod_4)[5, 1]
upper_2005_16 <- confint(mod_4)[5, 2]

table_s5_data <- Landsat_data %>%
    mutate(Spp_richness = factor(Spp_richness, levels = c("0",
        "1", "4", "16")), Year = factor(Year, levels = c("2010",
        "2000", "2005")))


mod_4 <- lmer(Cover ~ factor(Year) * factor(Spp_richness) +
    (1 | Block) + (1 | Spp_comp) + (1 | Plot), data = table_s5_data)

# summary(mod_4)$coefficients

mean_2010_0 <- summary(mod_4)$coefficients[1, 1]
se_2010_0 <- summary(mod_4)$coefficients[1, 2]
lower_2010_0 <- confint(mod_4)[5, 1]
upper_2010_0 <- confint(mod_4)[5, 2]

table_s5_data <- Landsat_data %>%
    mutate(Spp_richness = factor(Spp_richness, levels = c("1",
        "4", "16", "0")), Year = factor(Year, levels = c("2010",
        "2000", "2005")))


mod_4 <- lmer(Cover ~ factor(Year) * factor(Spp_richness) +
    (1 | Block) + (1 | Spp_comp) + (1 | Plot), data = table_s5_data)

# summary(mod_4)$coefficients

mean_2010_1 <- summary(mod_4)$coefficients[1, 1]
se_2010_1 <- summary(mod_4)$coefficients[1, 2]
lower_2010_1 <- confint(mod_4)[5, 1]
upper_2010_1 <- confint(mod_4)[5, 2]

table_s5_data <- Landsat_data %>%
    mutate(Spp_richness = factor(Spp_richness, levels = c("4",
        "16", "0", "1")), Year = factor(Year, levels = c("2010",
        "2000", "2005")))


mod_4 <- lmer(Cover ~ factor(Year) * factor(Spp_richness) +
    (1 | Block) + (1 | Spp_comp) + (1 | Plot), data = table_s5_data)

# summary(mod_4)$coefficients
```

```
mean_2010_4 <- summary(mod_4)$coefficients[1, 1]
se_2010_4 <- summary(mod_4)$coefficients[1, 2]
lower_2010_4 <- confint(mod_4)[5, 1]
upper_2010_4 <- confint(mod_4)[5, 2]


table_s5_data <- Landsat_data %>%
    mutate(Spp_richness = factor(Spp_richness, levels = c("16",
        "0", "1", "4")), Year = factor(Year, levels = c("2010",
        "2000", "2005")))


mod_4 <- lmer(Cover ~ factor(Year) * factor(Spp_richness) +
    (1 | Block) + (1 | Spp_comp) + (1 | Plot), data = table_s5_data)

# summary(mod_4)$coefficients

mean_2010_16 <- summary(mod_4)$coefficients[1, 1]
se_2010_16 <- summary(mod_4)$coefficients[1, 2]
lower_2010_16 <- confint(mod_4)[5, 1]
upper_2010_16 <- confint(mod_4)[5, 2]

table_s5 <- tibble(epoch = rep(c("2000", "2005", "2010"),
    each = 4), species = rep(c("0", "1", "4", "16"), times = 3),
    cover_estimate = c(mean_2000_0, mean_2000_1, mean_2000_4,
        mean_2000_16, mean_2005_0, mean_2005_1, mean_2005_4,
        mean_2005_16, mean_2010_0, mean_2010_1, mean_2010_4,
        mean_2010_16), cover_se = c(se_2000_0, se_2000_1,
        se_2000_4, se_2000_16, se_2005_0, se_2005_1, se_2005_4,
        se_2005_16, se_2010_0, se_2010_1, se_2010_4, se_2010_16),
    cover_lower = c(lower_2000_0, lower_2000_1, lower_2000_4,
        lower_2000_16, lower_2005_0, lower_2005_1, lower_2005_4,
        lower_2005_16, lower_2010_0, lower_2010_1, lower_2010_4,
        lower_2010_16), cover_upper = c(upper_2000_0, upper_2000_1,
        upper_2000_4, upper_2000_16, upper_2005_0, upper_2005_1,
        upper_2005_4, upper_2005_16, upper_2010_0, upper_2010_1,
        upper_2010_4, upper_2010_16))
```

6.2 Graphing

```
dodge <- position_dodge(0.3)
epoch_labels <- c("1999-2002", "2003-2008", "2008-2012")

cols_2 <- RColorBrewer::brewer.pal(5, "Set1")

fig_2b <- table_s5 %>%
    mutate(epoch = as.character(epoch)) %>%
    ggplot(aes(epoch, cover_estimate, colour = as.factor(species),
        group = as.factor(species))) + geom_line(position = dodge,
    aes(linetype = as.factor(species))) + geom_pointrange(aes(x = epoch,
    y = cover_estimate, ymin = cover_lower, ymax = cover_upper,
```

```
    shape = as.factor(species)), position = dodge, fill = "white",
    ) + scale_shape_manual(values = c(21:24)) + scale_colour_manual(values = cols_2) +
    scale_x_discrete(labels = epoch_labels) + labs(x = "Time period",
    y = "Vegetation cover (%)", col = "Tree planted \nspecies richness",
    shape = "Tree planted \nspecies richness", linetype = "Tree planted \nspecies richness") +
    theme(legend.position = "none")

fig_2b
```

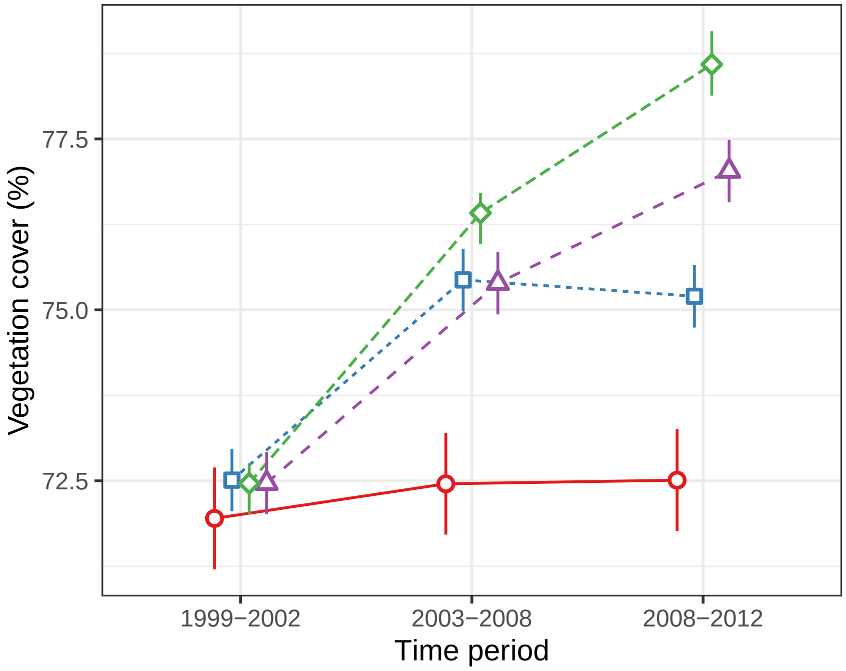

7 Effect of genus diversity and canopy strucutral complexity

7.1 Modelling

Model used:

$$mod\_5 \leftarrow lmer(y \sim factor(Spp\_richness) + Gen\_div + Canopy\_type + Climber\_cutting + (1|Block) + (1|Spp\_comp), data =$$

We tested for an interaction between genus diversity and canopy structural complexity, and found no effect.

```
table_s7_data <- Rapideye_data %>%
    mutate(Spp_richness = factor(Spp_richness, levels = c("4",
        "16", "0", "1"))) %>%
    mutate(Gen_div = factor(Gen_div, levels = c("4", "2",
        "0", "5"))) %>%
    mutate(Canopy_type = factor(Canopy_type, levels = c("Thick",
        "Thin", "0")))
```

```
mod_5_Biomass <- lmer(Biomass ~ factor(Spp_richness) +
    Gen_div + Canopy_type + Climber_cutting + (1 | Block) +
    (1 | Spp_comp), data = table_s7_data)
mod_5b_Biomass <- lmer(Biomass ~ factor(Spp_richness) +
    Gen_div * Canopy_type + Climber_cutting + (1 | Block) +
    (1 | Spp_comp), data = table_s7_data)
```

```
anova(mod_5_Biomass, mod_5b_Biomass)
```

```
## Data: table_s7_data
## Models:
## mod_5_Biomass: Biomass ~ factor(Spp_richness) + Gen_div + Canopy_type + Climber_cutting + (1 | Block
## mod_5b_Biomass: Biomass ~ factor(Spp_richness) + Gen_div * Canopy_type + Climber_cutting + (1 | Bloc
##                npar    AIC    BIC  logLik deviance Chisq Df Pr(>Chisq)
## mod_5_Biomass    10 996.51 1024.7 -488.25   976.51
## mod_5b_Biomass   11 998.51 1029.5 -488.25   976.51 7e-04  1     0.9791
```

```
table_s7_data <- Rapideye_data %>%
    mutate(Spp_richness = factor(Spp_richness, levels = c("4",
        "16", "0", "1"))) %>%
    mutate(Gen_div = factor(Gen_div, levels = c("4", "2",
        "0", "5"))) %>%
    mutate(Canopy_type = factor(Canopy_type, levels = c("Thick",
        "Thin", "0")))
mod_5_Biomass <- lmer(Biomass ~ factor(Spp_richness) +
    Gen_div + Canopy_type + Climber_cutting + (1 | Block) +
    (1 | Spp_comp), data = table_s7_data)

summary(mod_5_Biomass)$coefficients
```

```
##                         Estimate Std. Error         df    t value     Pr(>|t|)
## (Intercept)           232.262969   4.615959   7.272451 50.3173854 1.620988e-10
## factor(Spp_richness)16  29.288919   4.373583 116.075308  6.6967792 7.995965e-10
## factor(Spp_richness)0  -49.596818   5.307499 116.000000 -9.3446682 8.152880e-16
## factor(Spp_richness)1  -17.638238   4.459191 116.000000 -3.9554793 1.319006e-04
## Gen_div2               -3.328448   4.459191 116.000000 -0.7464241 4.569214e-01
## Canopy_typeThin          2.595778   4.459191 116.000000  0.5821186 5.616165e-01
## Climber_cuttingYes       4.176099   4.651873 116.292973  0.8977242 3.711874e-01
```

```
mean_4_thick_4 <- summary(mod_5_Biomass)$coefficients[1,
    1]
se_4_thick_4 <- summary(mod_5_Biomass)$coefficients[1,
    2]
lower_4_thick_4 <- confint(mod_5_Biomass)[4, 1]
upper_4_thick_4 <- confint(mod_5_Biomass)[4, 2]

table_s7_data <- Rapideye_data %>%
    mutate(Spp_richness = factor(Spp_richness, levels = c("4",
        "16", "0", "1"))) %>%
    mutate(Gen_div = factor(Gen_div, levels = c("2", "4",
        "0", "5"))) %>%
    mutate(Canopy_type = factor(Canopy_type, levels = c("Thick",
```

```
        "Thin", "0")))
mod_5_Biomass <- lmer(Biomass ~ factor(Spp_richness) +
    Gen_div + Canopy_type + Climber_cutting + (1 | Block) +
    (1 | Spp_comp), data = table_s7_data)
mean_4_thick_2 <- summary(mod_5_Biomass)$coefficients[1,
    1]
se_4_thick_2 <- summary(mod_5_Biomass)$coefficients[1,
    2]
lower_4_thick_2 <- confint(mod_5_Biomass)[4, 1]
upper_4_thick_2 <- confint(mod_5_Biomass)[4, 2]

table_s7_data <- Rapideye_data %>%
    mutate(Spp_richness = factor(Spp_richness, levels = c("4",
        "16", "0", "1"))) %>%
    mutate(Gen_div = factor(Gen_div, levels = c("4", "2",
        "0", "5"))) %>%
    mutate(Canopy_type = factor(Canopy_type, levels = c("Thin",
        "Thick", "0")))
mod_5_Biomass <- lmer(Biomass ~ factor(Spp_richness) +
    Gen_div + Canopy_type + Climber_cutting + (1 | Block) +
    (1 | Spp_comp), data = table_s7_data)
mean_4_thin_4 <- summary(mod_5_Biomass)$coefficients[1,
    1]
se_4_thin_4 <- summary(mod_5_Biomass)$coefficients[1, 2]
lower_4_thin_4 <- confint(mod_5_Biomass)[4, 1]
upper_4_thin_4 <- confint(mod_5_Biomass)[4, 2]

table_s7_data <- Rapideye_data %>%
    mutate(Spp_richness = factor(Spp_richness, levels = c("4",
        "16", "0", "1"))) %>%
    mutate(Gen_div = factor(Gen_div, levels = c("2", "4",
        "0", "5"))) %>%
    mutate(Canopy_type = factor(Canopy_type, levels = c("Thin",
        "Thick", "0")))
mod_5_Biomass <- lmer(Biomass ~ factor(Spp_richness) +
    Gen_div + Canopy_type + Climber_cutting + (1 | Block) +
    (1 | Spp_comp), data = table_s7_data)
mean_4_thin_2 <- summary(mod_5_Biomass)$coefficients[1,
    1]
se_4_thin_2 <- summary(mod_5_Biomass)$coefficients[1, 2]
lower_4_thin_2 <- confint(mod_5_Biomass)[4, 1]
upper_4_thin_2 <- confint(mod_5_Biomass)[4, 2]

table_s7_Biomass <- tibble(genera = c("2", "4", "2", "4"),
    canopy_type = c("Thin", "Thin", "Thick", "Thick"),
    Biomass_mean = c(mean_4_thin_2, mean_4_thin_4, mean_4_thick_2,
        mean_4_thick_4), Biomass_se = c(se_4_thin_2, se_4_thin_4,
        se_4_thick_2, se_4_thick_4), Biomass_lower = c(lower_4_thin_2,
        lower_4_thin_4, lower_4_thick_2, lower_4_thick_4),
    Biomass_upper = c(upper_4_thin_2, upper_4_thin_4, upper_4_thick_2,
        upper_4_thick_4), )
```

```
table_s7_data <- Rapideye_data %>%
    mutate(Spp_richness = factor(Spp_richness, levels = c("4",
        "16", "0", "1"))) %>%
    mutate(Gen_div = factor(Gen_div, levels = c("4", "2",
        "0", "5"))) %>%
    mutate(Canopy_type = factor(Canopy_type, levels = c("Thick",
        "Thin", "0")))
mod_5_LAI <- lmer(LAI ~ factor(Spp_richness) + Gen_div +
    Canopy_type + Climber_cutting + (1 | Block) + (1 |
    Spp_comp), data = table_s7_data)
mean_4_thick_4 <- summary(mod_5_LAI)$coefficients[1, 1]
se_4_thick_4 <- summary(mod_5_LAI)$coefficients[1, 2]
lower_4_thick_4 <- confint(mod_5_LAI)[4, 1]
upper_4_thick_4 <- confint(mod_5_LAI)[4, 2]

table_s7_data <- Rapideye_data %>%
    mutate(Spp_richness = factor(Spp_richness, levels = c("4",
        "16", "0", "1"))) %>%
    mutate(Gen_div = factor(Gen_div, levels = c("2", "4",
        "0", "5"))) %>%
    mutate(Canopy_type = factor(Canopy_type, levels = c("Thick",
        "Thin", "0")))
mod_5_LAI <- lmer(LAI ~ factor(Spp_richness) + Gen_div +
    Canopy_type + Climber_cutting + (1 | Block) + (1 |
    Spp_comp), data = table_s7_data)
mean_4_thick_2 <- summary(mod_5_LAI)$coefficients[1, 1]
se_4_thick_2 <- summary(mod_5_LAI)$coefficients[1, 2]
lower_4_thick_2 <- confint(mod_5_LAI)[4, 1]
upper_4_thick_2 <- confint(mod_5_LAI)[4, 2]

table_s7_data <- Rapideye_data %>%
    mutate(Spp_richness = factor(Spp_richness, levels = c("4",
        "16", "0", "1"))) %>%
    mutate(Gen_div = factor(Gen_div, levels = c("4", "2",
        "0", "5"))) %>%
    mutate(Canopy_type = factor(Canopy_type, levels = c("Thin",
        "Thick", "0")))
mod_5_LAI <- lmer(LAI ~ factor(Spp_richness) + Gen_div +
    Canopy_type + Climber_cutting + (1 | Block) + (1 |
    Spp_comp), data = table_s7_data)
mean_4_thin_4 <- summary(mod_5_LAI)$coefficients[1, 1]
se_4_thin_4 <- summary(mod_5_LAI)$coefficients[1, 2]
lower_4_thin_4 <- confint(mod_5_LAI)[4, 1]
upper_4_thin_4 <- confint(mod_5_LAI)[4, 2]

table_s7_data <- Rapideye_data %>%
    mutate(Spp_richness = factor(Spp_richness, levels = c("4",
        "16", "0", "1"))) %>%
    mutate(Gen_div = factor(Gen_div, levels = c("2", "4",
        "0", "5"))) %>%
    mutate(Canopy_type = factor(Canopy_type, levels = c("Thin",
        "Thick", "0")))
```

```r
mod_5_LAI <- lmer(LAI ~ factor(Spp_richness) + Gen_div +
    Canopy_type + Climber_cutting + (1 | Block) + (1 |
    Spp_comp), data = table_s7_data)
mean_4_thin_2 <- summary(mod_5_LAI)$coefficients[1, 1]
se_4_thin_2 <- summary(mod_5_LAI)$coefficients[1, 2]
lower_4_thin_2 <- confint(mod_5_LAI)[4, 1]
upper_4_thin_2 <- confint(mod_5_LAI)[4, 2]

table_s7_LAI <- tibble(genera = c("2", "4", "2", "4"),
    canopy_type = c("Thin", "Thin", "Thick", "Thick"),
    LAI_mean = c(mean_4_thin_2, mean_4_thin_4, mean_4_thick_2,
        mean_4_thick_4), LAI_se = c(se_4_thin_2, se_4_thin_4,
        se_4_thick_2, se_4_thick_4), LAI_lower = c(lower_4_thin_2,
        lower_4_thin_4, lower_4_thick_2, lower_4_thick_4),
    LAI_upper = c(upper_4_thin_2, upper_4_thin_4, upper_4_thick_2,
        upper_4_thick_4), )




table_s7_data <- Rapideye_data %>%
    mutate(Spp_richness = factor(Spp_richness, levels = c("4",
        "16", "0", "1"))) %>%
    mutate(Gen_div = factor(Gen_div, levels = c("4", "2",
        "0", "5"))) %>%
    mutate(Canopy_type = factor(Canopy_type, levels = c("Thick",
        "Thin", "0")))
mod_5_Cover <- lmer(Cover ~ factor(Spp_richness) + Gen_div +
    Canopy_type + Climber_cutting + (1 | Block) + (1 |
    Spp_comp), data = table_s7_data)
mean_4_thick_4 <- summary(mod_5_Cover)$coefficients[1,
    1]
se_4_thick_4 <- summary(mod_5_Cover)$coefficients[1, 2]
lower_4_thick_4 <- confint(mod_5_Cover)[4, 1]
upper_4_thick_4 <- confint(mod_5_Cover)[4, 2]

table_s7_data <- Rapideye_data %>%
    mutate(Spp_richness = factor(Spp_richness, levels = c("4",
        "16", "0", "1"))) %>%
    mutate(Gen_div = factor(Gen_div, levels = c("2", "4",
        "0", "5"))) %>%
    mutate(Canopy_type = factor(Canopy_type, levels = c("Thick",
        "Thin", "0")))
mod_5_Cover <- lmer(Cover ~ factor(Spp_richness) + Gen_div +
    Canopy_type + Climber_cutting + (1 | Block) + (1 |
    Spp_comp), data = table_s7_data)
mean_4_thick_2 <- summary(mod_5_Cover)$coefficients[1,
    1]
se_4_thick_2 <- summary(mod_5_Cover)$coefficients[1, 2]
lower_4_thick_2 <- confint(mod_5_Cover)[4, 1]
upper_4_thick_2 <- confint(mod_5_Cover)[4, 2]

table_s7_data <- Rapideye_data %>%
```

```
    mutate(Spp_richness = factor(Spp_richness, levels = c("4",
        "16", "0", "1"))) %>%
    mutate(Gen_div = factor(Gen_div, levels = c("4", "2",
        "0", "5"))) %>%
    mutate(Canopy_type = factor(Canopy_type, levels = c("Thin",
        "Thick", "0")))
mod_5_Cover <- lmer(Cover ~ factor(Spp_richness) + Gen_div +
    Canopy_type + Climber_cutting + (1 | Block) + (1 |
    Spp_comp), data = table_s7_data)
mean_4_thin_4 <- summary(mod_5_Cover)$coefficients[1, 1]
se_4_thin_4 <- summary(mod_5_Cover)$coefficients[1, 2]
lower_4_thin_4 <- confint(mod_5_Cover)[4, 1]
upper_4_thin_4 <- confint(mod_5_Cover)[4, 2]

table_s7_data <- Rapideye_data %>%
    mutate(Spp_richness = factor(Spp_richness, levels = c("4",
        "16", "0", "1"))) %>%
    mutate(Gen_div = factor(Gen_div, levels = c("2", "4",
        "0", "5"))) %>%
    mutate(Canopy_type = factor(Canopy_type, levels = c("Thin",
        "Thick", "0")))
mod_5_Cover <- lmer(Cover ~ factor(Spp_richness) + Gen_div +
    Canopy_type + Climber_cutting + (1 | Block) + (1 |
    Spp_comp), data = table_s7_data)
mean_4_thin_2 <- summary(mod_5_Cover)$coefficients[1, 1]
se_4_thin_2 <- summary(mod_5_Cover)$coefficients[1, 2]
lower_4_thin_2 <- confint(mod_5_Cover)[4, 1]
upper_4_thin_2 <- confint(mod_5_Cover)[4, 2]

table_s7_Cover <- tibble(genera = c("2", "4", "2", "4"),
    canopy_type = c("Thin", "Thin", "Thick", "Thick"),
    Cover_mean = c(mean_4_thin_2, mean_4_thin_4, mean_4_thick_2,
        mean_4_thick_4), Cover_se = c(se_4_thin_2, se_4_thin_4,
        se_4_thick_2, se_4_thick_4), Cover_lower = c(lower_4_thin_2,
        lower_4_thin_4, lower_4_thick_2, lower_4_thick_4),
    Cover_upper = c(upper_4_thin_2, upper_4_thin_4, upper_4_thick_2,
        upper_4_thick_4), )

table_s7 <- table_s7_Biomass %>%
    merge(table_s7_LAI, by = c("genera", "canopy_type")) %>%
    merge(table_s7_Cover, by = c("genera", "canopy_type"))
```

7.2 Graphing

For visualization, we fit models that generate estimates for 2 vs. 4 genera and for low vs. high canopy structural diversity.

```
table_s7_2_data <- Rapideye_data %>%
    mutate(Spp_richness = factor(Spp_richness, levels = c("4",
        "1", "0", "16"))) %>%
    mutate(Gen_div = factor(Gen_div, levels = c("4", "2",
        "0", "5")))
```

```
mod_s7_2_Biomass <- lmer(Biomass ~ factor(Spp_richness) +
    Gen_div + Treatment + (1 | Block) + (1 | Spp_comp),
    data = table_s7_2_data)

mean_4_4 <- summary(mod_s7_2_Biomass)$coefficients[1, 1]
se_4_4 <- summary(mod_s7_2_Biomass)$coefficients[1, 2]
lower_4_4 <- confint(mod_s7_2_Biomass)[4, 1]
upper_4_4 <- confint(mod_s7_2_Biomass)[4, 2]

table_s7_2_data <- Rapideye_data %>%
    mutate(Spp_richness = factor(Spp_richness, levels = c("4",
        "1", "0", "16"))) %>%
    mutate(Gen_div = factor(Gen_div, levels = c("2", "4",
        "0", "5")))
mod_s7_2_Biomass <- lmer(Biomass ~ factor(Spp_richness) +
    Gen_div + Treatment + (1 | Block) + (1 | Spp_comp),
    data = table_s7_2_data)

mean_4_2 <- summary(mod_s7_2_Biomass)$coefficients[1, 1]
se_4_2 <- summary(mod_s7_2_Biomass)$coefficients[1, 2]
lower_4_2 <- confint(mod_s7_2_Biomass)[4, 1]
upper_4_2 <- confint(mod_s7_2_Biomass)[4, 2]

table_s7_2_data <- Rapideye_data %>%
    mutate(Spp_richness = factor(Spp_richness, levels = c("4",
        "1", "0", "16"))) %>%
    mutate(Canopy_type = factor(Canopy_type, levels = c("Thick",
        "Thin", "0")))
mod_s7_2_Biomass <- lmer(Biomass ~ factor(Spp_richness) +
    Canopy_type + Treatment + (1 | Block) + (1 | Spp_comp),
    data = table_s7_2_data)

mean_4_thick <- summary(mod_s7_2_Biomass)$coefficients[1,
    1]
se_4_thick <- summary(mod_s7_2_Biomass)$coefficients[1,
    2]
lower_4_thick <- confint(mod_s7_2_Biomass)[4, 1]
upper_4_thick <- confint(mod_s7_2_Biomass)[4, 2]

table_s7_2_data <- Rapideye_data %>%
    mutate(Spp_richness = factor(Spp_richness, levels = c("4",
        "1", "0", "16"))) %>%
    mutate(Canopy_type = factor(Canopy_type, levels = c("Thin",
        "Thick", "0")))
mod_s7_2_Biomass <- lmer(Biomass ~ factor(Spp_richness) +
    Canopy_type + Treatment + (1 | Block) + (1 | Spp_comp),
    data = table_s7_2_data)

mean_4_thin <- summary(mod_s7_2_Biomass)$coefficients[1,
    1]
se_4_thin <- summary(mod_s7_2_Biomass)$coefficients[1,
    2]
lower_4_thin <- confint(mod_s7_2_Biomass)[4, 1]
```

```
upper_4_thin <- confint(mod_s7_2_Biomass)[4, 2]

table_s7_2_Biomass <- tibble(treatment = c("2_genera",
    "4_genera", "low_complexity", "high_complexity"), Biomass_mean = c(mean_4_2,
    mean_4_4, mean_4_thin, mean_4_thick), Biomass_se = c(se_4_2,
    se_4_4, se_4_thin, se_4_thick), Biomass_lower = c(lower_4_2,
    lower_4_4, lower_4_thin, lower_4_thick), Biomass_upper = c(upper_4_2,
    upper_4_4, upper_4_thin, upper_4_thick), )
```

```
table_s7_2_data <- Rapideye_data %>%
    mutate(Spp_richness = factor(Spp_richness, levels = c("4",
        "1", "0", "16"))) %>%
    mutate(Gen_div = factor(Gen_div, levels = c("4", "2",
        "0", "5")))
mod_s7_2_LAI <- lmer(LAI ~ factor(Spp_richness) + Gen_div +
    Treatment + (1 | Block) + (1 | Spp_comp), data = table_s7_2_data)

mean_4_4 <- summary(mod_s7_2_LAI)$coefficients[1, 1]
se_4_4 <- summary(mod_s7_2_LAI)$coefficients[1, 2]
lower_4_4 <- confint(mod_s7_2_LAI)[4, 1]
upper_4_4 <- confint(mod_s7_2_LAI)[4, 2]

table_s7_2_data <- Rapideye_data %>%
    mutate(Spp_richness = factor(Spp_richness, levels = c("4",
        "1", "0", "16"))) %>%
    mutate(Gen_div = factor(Gen_div, levels = c("2", "4",
        "0", "5")))
mod_s7_2_LAI <- lmer(LAI ~ factor(Spp_richness) + Gen_div +
    Treatment + (1 | Block) + (1 | Spp_comp), data = table_s7_2_data)

mean_4_2 <- summary(mod_s7_2_LAI)$coefficients[1, 1]
se_4_2 <- summary(mod_s7_2_LAI)$coefficients[1, 2]
lower_4_2 <- confint(mod_s7_2_LAI)[4, 1]
upper_4_2 <- confint(mod_s7_2_LAI)[4, 2]

table_s7_2_data <- Rapideye_data %>%
    mutate(Spp_richness = factor(Spp_richness, levels = c("4",
        "1", "0", "16"))) %>%
    mutate(Canopy_type = factor(Canopy_type, levels = c("Thick",
        "Thin", "0")))
mod_s7_2_LAI <- lmer(LAI ~ factor(Spp_richness) + Canopy_type +
    Treatment + (1 | Block) + (1 | Spp_comp), data = table_s7_2_data)

mean_4_thick <- summary(mod_s7_2_LAI)$coefficients[1, 1]
se_4_thick <- summary(mod_s7_2_LAI)$coefficients[1, 2]
lower_4_thick <- confint(mod_s7_2_LAI)[4, 1]
upper_4_thick <- confint(mod_s7_2_LAI)[4, 2]

table_s7_2_data <- Rapideye_data %>%
    mutate(Spp_richness = factor(Spp_richness, levels = c("4",
        "1", "0", "16"))) %>%
    mutate(Canopy_type = factor(Canopy_type, levels = c("Thin",
        "Thick", "0")))
```

```
mod_s7_2_LAI <- lmer(LAI ~ factor(Spp_richness) + Canopy_type +
    Treatment + (1 | Block) + (1 | Spp_comp), data = table_s7_2_data)

mean_4_thin <- summary(mod_s7_2_LAI)$coefficients[1, 1]
se_4_thin <- summary(mod_s7_2_LAI)$coefficients[1, 2]
lower_4_thin <- confint(mod_s7_2_LAI)[4, 1]
upper_4_thin <- confint(mod_s7_2_LAI)[4, 2]

table_s7_2_LAI <- tibble(treatment = c("2_genera", "4_genera",
    "low_complexity", "high_complexity"), LAI_mean = c(mean_4_2,
    mean_4_4, mean_4_thin, mean_4_thick), LAI_se = c(se_4_2,
    se_4_4, se_4_thin, se_4_thick), LAI_lower = c(lower_4_2,
    lower_4_4, lower_4_thin, lower_4_thick), LAI_upper = c(upper_4_2,
    upper_4_4, upper_4_thin, upper_4_thick), )
```

```
table_s7_2_data <- Rapideye_data %>%
    mutate(Spp_richness = factor(Spp_richness, levels = c("4",
        "1", "0", "16"))) %>%
    mutate(Gen_div = factor(Gen_div, levels = c("4", "2",
        "0", "5")))
mod_s7_2_Cover <- lmer(Cover ~ factor(Spp_richness) + Gen_div +
    Treatment + (1 | Block) + (1 | Spp_comp), data = table_s7_2_data)

mean_4_4 <- summary(mod_s7_2_Cover)$coefficients[1, 1]
se_4_4 <- summary(mod_s7_2_Cover)$coefficients[1, 2]
lower_4_4 <- confint(mod_s7_2_Cover)[4, 1]
upper_4_4 <- confint(mod_s7_2_Cover)[4, 2]

table_s7_2_data <- Rapideye_data %>%
    mutate(Spp_richness = factor(Spp_richness, levels = c("4",
        "1", "0", "16"))) %>%
    mutate(Gen_div = factor(Gen_div, levels = c("2", "4",
        "0", "5")))
mod_s7_2_Cover <- lmer(Cover ~ factor(Spp_richness) + Gen_div +
    Treatment + (1 | Block) + (1 | Spp_comp), data = table_s7_2_data)

mean_4_2 <- summary(mod_s7_2_Cover)$coefficients[1, 1]
se_4_2 <- summary(mod_s7_2_Cover)$coefficients[1, 2]
lower_4_2 <- confint(mod_s7_2_Cover)[4, 1]
upper_4_2 <- confint(mod_s7_2_Cover)[4, 2]

table_s7_2_data <- Rapideye_data %>%
    mutate(Spp_richness = factor(Spp_richness, levels = c("4",
        "1", "0", "16"))) %>%
    mutate(Canopy_type = factor(Canopy_type, levels = c("Thick",
        "Thin", "0")))
mod_s7_2_Cover <- lmer(Cover ~ factor(Spp_richness) + Canopy_type +
    Treatment + (1 | Block) + (1 | Spp_comp), data = table_s7_2_data)

mean_4_thick <- summary(mod_s7_2_Cover)$coefficients[1,
    1]
se_4_thick <- summary(mod_s7_2_Cover)$coefficients[1, 2]
lower_4_thick <- confint(mod_s7_2_Cover)[4, 1]
```

```
upper_4_thick <- confint(mod_s7_2_Cover)[4, 2]

table_s7_2_data <- Rapideye_data %>%
    mutate(Spp_richness = factor(Spp_richness, levels = c("4",
        "1", "0", "16"))) %>%
    mutate(Canopy_type = factor(Canopy_type, levels = c("Thin",
        "Thick", "0")))
mod_s7_2_Cover <- lmer(Cover ~ factor(Spp_richness) + Canopy_type +
    Treatment + (1 | Block) + (1 | Spp_comp), data = table_s7_2_data)

mean_4_thin <- summary(mod_s7_2_Cover)$coefficients[1,
    1]
se_4_thin <- summary(mod_s7_2_Cover)$coefficients[1, 2]
lower_4_thin <- confint(mod_s7_2_Cover)[4, 1]
upper_4_thin <- confint(mod_s7_2_Cover)[4, 2]

table_s7_2_Cover <- tibble(treatment = c("2_genera", "4_genera",
    "low_complexity", "high_complexity"), Cover_mean = c(mean_4_2,
    mean_4_4, mean_4_thin, mean_4_thick), Cover_se = c(se_4_2,
    se_4_4, se_4_thin, se_4_thick), Cover_lower = c(lower_4_2,
    lower_4_4, lower_4_thin, lower_4_thick), Cover_upper = c(upper_4_2,
    upper_4_4, upper_4_thin, upper_4_thick), )
```

```
table_s7_2 <- merge(table_s7_2_Biomass, table_s7_2_LAI,
    by = "treatment") %>%
    merge(table_s7_2_Cover, by = "treatment")
```

AGB - Canopy structural complexity (fig 1h)

```
fig_1H_data <- table_s7_2 %>%
    select(1, 2, contains("Biomass")) %>%
    filter(str_detect(treatment, "complexity")) %>%
    mutate(canopy_type = c("High", "Low")) %>%
    mutate(canopy_type = factor(canopy_type, levels = c("Low",
        "High")))


fig_1h <- fig_1H_data %>%
    ggplot(aes(x = canopy_type, y = Biomass_mean)) + geom_pointrange(aes(ymin = Biomass_lower,
    ymax = Biomass_upper, colour = canopy_type, shape = canopy_type),
    size = 1, fill = "white", shape = 22) + scale_colour_manual(values = c(cols[1],
    cols[2])) + theme(legend.position = "none") + labs(y = expression(atop("Aboveground biomass",
    paste("(Mg  ", Ha^-1, ")"))), x = "Canopy structural complexity")


fig_1h
```

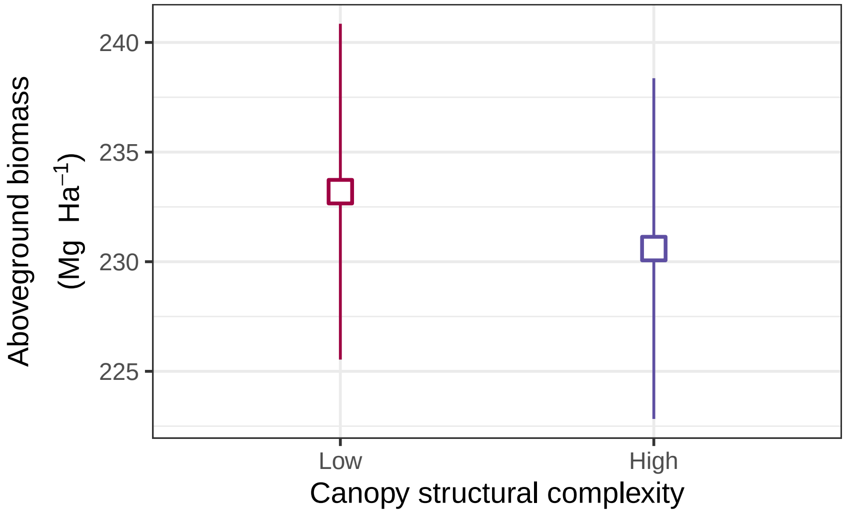

7.2.1 AGB - Genus diversity (fig 1i)

```
fig_1i_data <- table_s7_2 %>%
    select(1, 2, contains("Biomass")) %>%
    filter(str_detect(treatment, "genera")) %>%
    mutate(genera = c("2", "4"))

fig_1i <- fig_1i_data %>%
    ggplot(aes(x = genera, y = Biomass_mean)) + geom_pointrange(aes(ymin = Biomass_lower,
    ymax = Biomass_upper, colour = genera, shape = genera),
    size = 1, fill = "white", shape = 22) + scale_colour_manual(values = c(cols[1],
    cols[2])) + theme(legend.position = "none") + labs(y = expression(atop("Aboveground biomass",
    paste("(Mg  ", Ha^-1, ")"))), x = "Genus diversity")


fig_1i
```

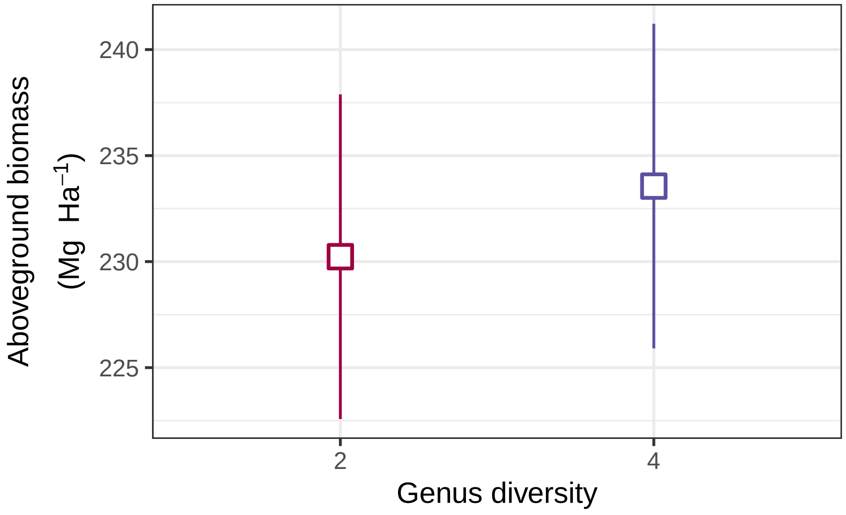

7.2.2 LAI - Canopy structural complexity (fig S4c)

```
fig_s4_data <- table_s7_2 %>%
    select(1, contains("LAI")) %>%
    filter(str_detect(treatment, "complexity")) %>%
    mutate(canopy_type = c("High", "Low")) %>%
    select(-treatment)

fig_s4b <- fig_s4_data %>%
    ggplot(aes(x = canopy_type, y = LAI_mean)) + geom_pointrange(aes(ymin = LAI_lower,
    ymax = LAI_upper, colour = canopy_type, shape = canopy_type),
    size = 1, fill = "white", shape = 22) + scale_colour_manual(values = c(cols[1],
    cols[2])) + theme(legend.position = "none") + scale_y_continuous(limits = c(4,
    6.5)) + labs(y = "", x = "")

fig_s4b
```

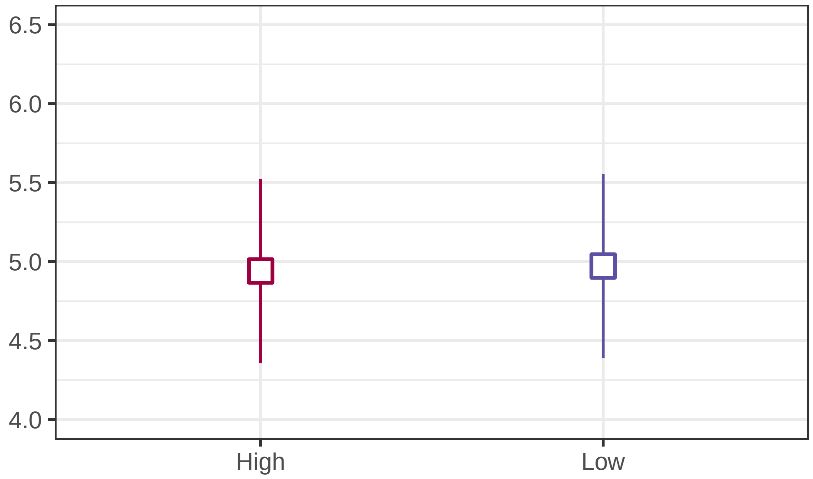

7.2.3 LAI - Genus diversity (fig S4c)

```
fig_s4_c_data <- table_s7_2 %>%
    select(1, contains("LAI")) %>%
    filter(str_detect(treatment, "genera")) %>%
    mutate(genera = c("2", "4"))

fig_s4c <- fig_s4_c_data %>%
    ggplot(aes(x = genera, y = LAI_mean)) + geom_pointrange(aes(ymin = LAI_lower,
    ymax = LAI_upper, colour = genera, shape = genera),
    size = 1, fill = "white", shape = 22) + scale_colour_manual(values = c(cols[1],
    cols[2])) + theme(legend.position = "none") + scale_y_continuous(limits = c(4,
    6.5)) + labs(y = "", x = "")

fig_s4c
```

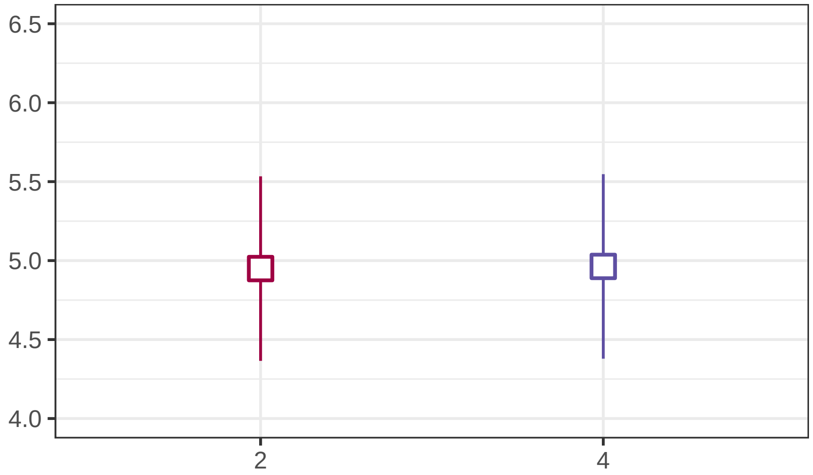

7.2.4 Cover - Canopy structural complexity (fig S4e)

```
fig_s4_e_data <- table_s7_2 %>%
    select(1, contains("Cover")) %>%
    filter(str_detect(treatment, "complexity")) %>%
    mutate(canopy_type = c("High", "Low")) %>%
    select(-treatment)

fig_s4e <- fig_s4_e_data %>%
    ggplot(aes(x = canopy_type, y = Cover_mean)) + geom_pointrange(aes(ymin = Cover_lower,
    ymax = Cover_upper, colour = canopy_type, shape = canopy_type),
    size = 1, fill = "white", shape = 22) + scale_colour_manual(values = c(cols[1],
    cols[2])) + scale_y_continuous(limits = c(60, 80)) +
    theme(legend.position = "none") + labs(y = "", x = "Canopy structural complexity")

fig_s4e
```

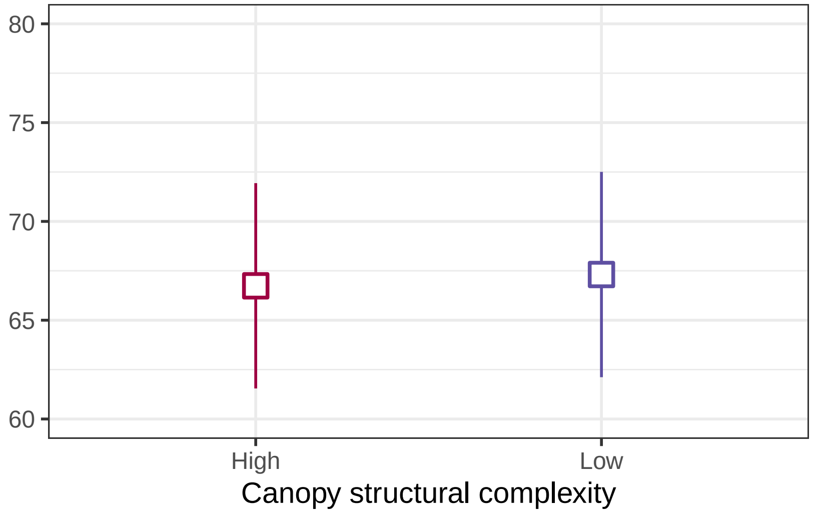

7.2.5 Cover - Genus diversity (fig S4f)

```
fig_s4_f_data <- table_s7_2 %>%
    select(1, contains("Cover")) %>%
    filter(str_detect(treatment, "genera")) %>%
    mutate(genera = c("2", "4")) %>%
    select(-treatment)

fig_s4f <- fig_s4_f_data %>%
    ggplot(aes(x = genera, y = Cover_mean)) + geom_pointrange(aes(ymin = Cover_lower,
    ymax = Cover_upper, colour = genera, shape = genera),
    size = 1, fill = "white", shape = 22) + scale_colour_manual(values = c(cols[1],
    cols[2])) + scale_y_continuous(limits = c(60, 80)) +
    theme(legend.position = "none") + labs(y = "", x = "Genus diversity")

fig_s4f
```

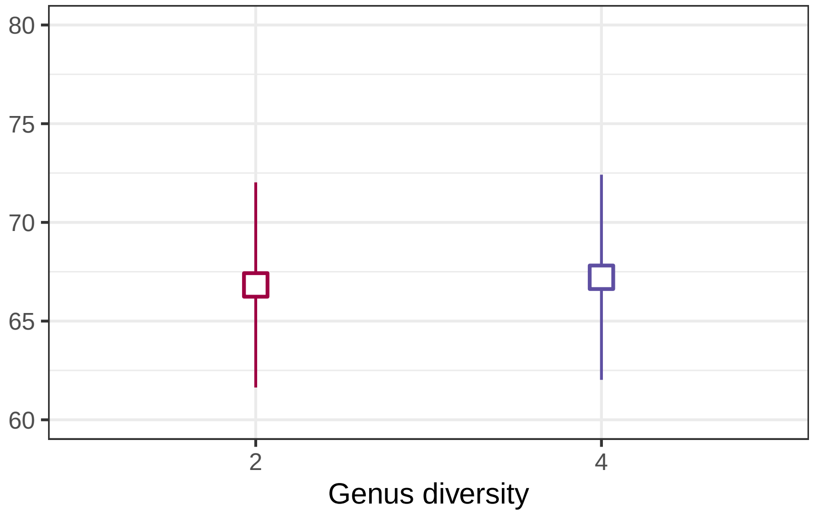

8 Effect of phylogenetic and functional diversity

8.1 Modelling

Model used:

$$mod\_6 \leftarrow lmer(Biomass \sim PD/FD + (1|Block) + (1|Spp\_comp))$$

```
table_s9_data <- Rapideye_data
# str(table_s9_data)
mod_6_mass <- lmer(Biomass ~  FD + (1|Block) + (1|Spp_comp), data = table_s9_data)

summary(mod_6_mass)$coefficients
```

```
##               Estimate Std. Error        df   t value     Pr(>|t|)
## (Intercept) 209.43783   3.679065  3.707485 56.926924 1.363949e-06
## FD           12.44149   1.594186 32.596377  7.804294 5.883259e-09
```

```
intercept_FD_all <- summary(mod_6_mass)$coefficients[1,1]
slope_FD_all <- summary(mod_6_mass)$coefficients[2,1]
intercept_lower_FD_all <- confint(mod_6_mass)[4,1]
intercept_upper_FD_all <- confint(mod_6_mass)[4,2]
slope_lower_FD_all <- confint(mod_6_mass)[5,1]
slope_upper_FD_all <- confint(mod_6_mass)[5,2]
intercept_se_FD_all <- summary(mod_6_mass)$coefficients[1,2]
slope_se_FD_all <- summary(mod_6_mass)$coefficients[2,2]

table_s9_data <- Rapideye_data

# str(table_s9_data)

mod_6_mass <- lmer(Biomass ~  FD + (1|Block) + (1|Spp_comp), subset = Spp_richness == 4, data = table_s9

summary(mod_6_mass)$coefficients
```

```
##                Estimate Std. Error       df   t value    Pr(>|t|)
## (Intercept) 223.054213   51.24936 29.14647 4.3523316 0.0001512488
## FD            4.215548   24.40073 29.00001 0.1727632 0.8640376767
```

```
intercept_FD_4 <- summary(mod_6_mass)$coefficients[1,1]
slope_FD_4 <- summary(mod_6_mass)$coefficients[2,1]
intercept_lower_FD_4 <- confint(mod_6_mass)[4,1]
intercept_upper_FD_4 <- confint(mod_6_mass)[4,2]
slope_lower_FD_4 <- confint(mod_6_mass)[5,1]
slope_upper_FD_4 <- confint(mod_6_mass)[5,2]
intercept_se_FD_4 <- summary(mod_6_mass)$coefficients[1,2]
slope_se_FD_4 <- summary(mod_6_mass)$coefficients[2,2]



table_s9_data <- Rapideye_data
# str(table_s9_data)
mod_6_mass <- lmer(Biomass ~  PD + (1|Block) + (1|Spp_comp), data = table_s9_data)

summary(mod_6_mass)$coefficients
```

```
##             Estimate Std. Error         df  t value     Pr(>|t|)
## (Intercept) 204.2292   3.103134   3.772312 65.81386 6.499639e-07
## PD          605.0990  36.872566 109.094819 16.41055 3.094923e-31
```

```
intercept_PD_all <- summary(mod_6_mass)$coefficients[1,1]
slope_PD_all <- summary(mod_6_mass)$coefficients[2,1]
intercept_lower_PD_all <- confint(mod_6_mass)[4,1]
intercept_upper_PD_all <- confint(mod_6_mass)[4,2]
slope_lower_PD_all <- confint(mod_6_mass)[5,1]
slope_upper_PD_all <- confint(mod_6_mass)[5,2]
```

```
intercept_se_PD_all <- summary(mod_6_mass)$coefficients[1,2]
slope_se_PD_all <- summary(mod_6_mass)$coefficients[2,2]



table_s9_data <- Rapideye_data

# str(table_s9_data)

mod_6_mass <- lmer(Biomass ~  PD + (1|Block) + (1|Spp_comp), subset = Spp_richness == 4, data = table_s9

summary(mod_6_mass)$coefficients
```

```
##              Estimate Std. Error       df   t value     Pr(>|t|)
## (Intercept)  226.346   5.787066 15.66233 39.112394 4.815789e-17
## PD           116.969 108.729690 29.00000  1.075778 2.908955e-01
```

```
intercept_PD_4 <- summary(mod_6_mass)$coefficients[1,1]
slope_PD_4 <- summary(mod_6_mass)$coefficients[2,1]
intercept_lower_PD_4 <- confint(mod_6_mass)[4,1]
intercept_upper_PD_4 <- confint(mod_6_mass)[4,2]
slope_lower_PD_4 <- confint(mod_6_mass)[5,1]
slope_upper_PD_4 <- confint(mod_6_mass)[5,2]
intercept_se_PD_4 <- summary(mod_6_mass)$coefficients[1,2]
slope_se_PD_4 <- summary(mod_6_mass)$coefficients[2,2]



table_s9 <- tibble(
  index = c("FD", "FD", "PD", "PD"),
  range = c("all", "4_spp","all", "4_spp"),
  intercept = c(intercept_FD_all, intercept_FD_4, intercept_PD_all, intercept_PD_4),
 #intercept_se = c(intercept_se_FD_all, intercept_se_FD_4, intercept_se_PD_all, intercept_se_PD_4),
  intercept_lower = c(intercept_lower_FD_all, intercept_lower_FD_4, intercept_lower_PD_all, intercept_lo
  intercept_upper = c(intercept_upper_FD_all, intercept_upper_FD_4, intercept_upper_PD_all, intercept_up
  slope = c(slope_FD_all, slope_FD_4, slope_PD_all, slope_PD_4),
 #slope_se = c(slope_se_FD_all, slope_se_FD_4, slope_se_PD_all, slope_se_PD_4),
  slope_lower = c(slope_lower_FD_all, slope_lower_FD_4, slope_lower_PD_all, slope_lower_PD_4),
  slope_upper = c(slope_upper_FD_all, slope_upper_FD_4, slope_upper_PD_all, slope_upper_PD_4))
```

8.2 Graphing

```
fig_3a_data <- table_s9 %>%
    filter(index == "PD")

data2 <- Sat_data %>%
    filter(Index == "Biomass" & Satellite == "RapidEye",
        Spp_richness == 4)

data_line_all <- tibble(x = seq(from = min(Rapideye_data$PD,
```

```
    na.rm = TRUE), to = max(Rapideye_data$PD, na.rm = TRUE),
    length.out = 10), y = x * fig_3a_data[[1, 6]] + fig_3a_data[[1,
    3]])

data_line_4 <- tibble(x = seq(from = min(Rapideye_data$PD[Rapideye_data$Spp_richness ==
    4], na.rm = TRUE), to = max(Rapideye_data$PD[Rapideye_data$Spp_richness ==
    4], na.rm = TRUE), length.out = 10), y = x * fig_3a_data[[2,
    6]] + fig_3a_data[[2, 3]])

fig_3a <- Sat_data %>%
    filter(Index == "Biomass") %>%
    filter(Spp_richness != 4) %>%
    ggplot(aes(PD, Score)) + geom_point() + geom_point(data = data2,
    aes(colour = Gen_div, shape = Canopy_type)) + geom_line(data = data_line_all,
    mapping = aes(x = x, y = y), size = 1, colour = "blue") +
    geom_line(data = data_line_4, mapping = aes(x = x,
        y = y), size = 1, linetype = 2, colour = "black") +
    labs(x = "Phylogenetic diversity", y = expression(paste("Aboveground biomass",
        " ", "(Mg h", a^-1, sep = "", ")")), shape = "Canopy\nstructural\ncomplexity",
        colour = "Genus\ndiversity") + scale_colour_brewer(palette = "Set1") +
    scale_shape_manual(values = c(15, 17)) + theme_bw()

fig_3a
```

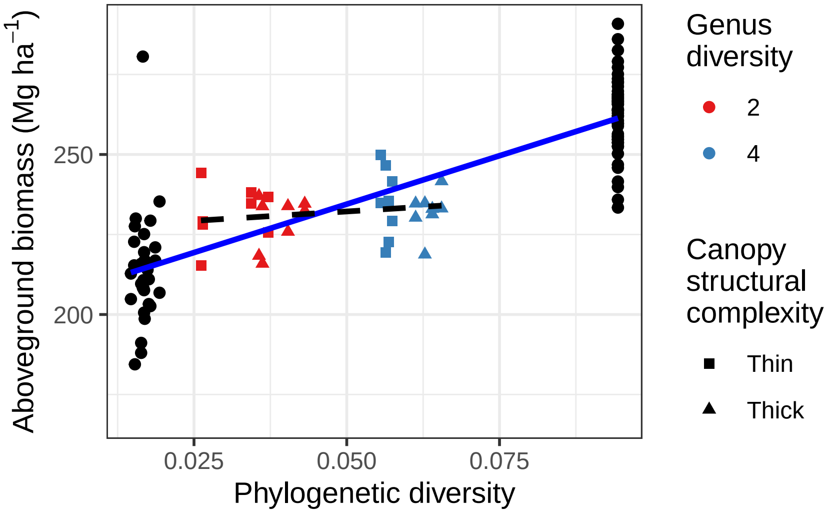


```
fig_3b_data <- table_s9 %>%
    filter(index == "FD")

data2 <- Sat_data %>%
    filter(Index == "Biomass" & Satellite == "RapidEye",
        Spp_richness == 4)

data_line_all <- tibble(x = seq(from = min(Rapideye_data$FD,
    na.rm = TRUE), to = max(Rapideye_data$FD, na.rm = TRUE),
    length.out = 10), y = x * fig_3b_data[[1, 6]] + fig_3b_data[[1,
    3]])
```

```
data_line_4 <- tibble(x = seq(from = min(Rapideye_data$FD[Rapideye_data$Spp_richness ==
    4], na.rm = TRUE), to = max(Rapideye_data$FD[Rapideye_data$Spp_richness ==
    4], na.rm = TRUE), length.out = 10), y = x * fig_3b_data[[2,
    6]] + fig_3b_data[[2, 3]])

fig_3b <- Sat_data %>%
    filter(Index == "Biomass") %>%
    filter(Spp_richness != 4) %>%
    ggplot(aes(FD, Score)) + geom_point() + geom_point(data = data2,
    aes(colour = Gen_div, shape = Canopy_type)) + geom_line(data = data_line_all,
    mapping = aes(x = x, y = y), size = 1, colour = "blue") +
    geom_line(data = data_line_4, mapping = aes(x = x,
        y = y), size = 1, linetype = 2, colour = "black") +
    # geom_smooth(data = data3, mapping = aes(x=FD,
    # y=Score), method = 'lm', se = FALSE) +
    # geom_smooth(data = data2, mapping = aes(x=FD,
    # y=Score), method = 'lm', se = FALSE, linetype =
    # 2, colour = 'black')+
labs(x = "Functional diversity", y = expression(paste("Aboveground biomass",
    " ", "(Mg h", a^-1, sep = "", ")")), shape = "Canopy\nstructural\ncomplexity",
    colour = "Genus\ndiversity") + scale_colour_brewer(palette = "Set1") +
    scale_shape_manual(values = c(15, 17)) + theme_bw()

fig_3b
```

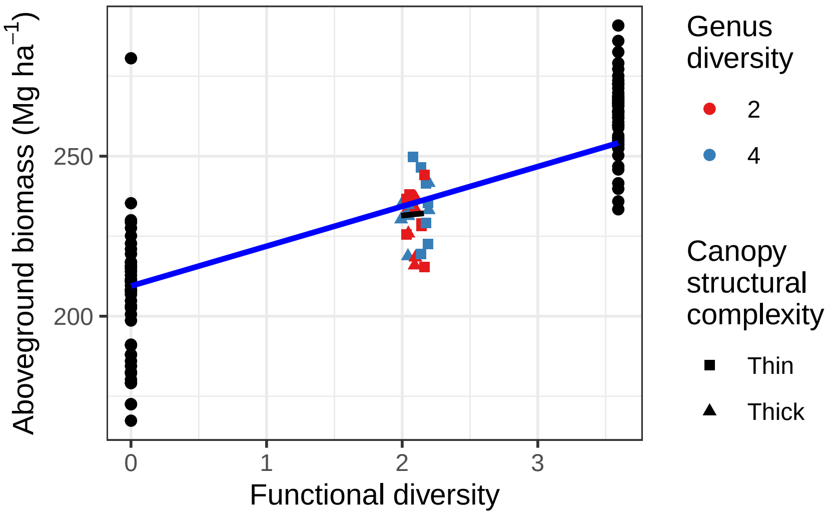

9 Effect of liana removal

9.1 Modelling

Model used:

$$mod\_5 \leftarrow lmer(y \sim factor(Spp\_richness) + Gen\_div + Canopy\_type + Climber_cutting + (1|Block) + (1|Spp\_comp))$$

```r
table_s8_data <- Rapideye_data %>%
    mutate(Climber_cutting = factor(Climber_cutting, levels = c("No",
        "Yes"))) %>%
    mutate(Spp_richness = factor(Spp_richness, levels = c("16",
        "4", "0", "1")))
mod_5_Biomass <- lmer(Biomass ~ factor(Spp_richness) +
    Gen_div + Canopy_type + Climber_cutting + (1 | Block) +
    (1 | Spp_comp), data = table_s8_data)
mean_16_spp <- summary(mod_5_Biomass)$coefficients[1, 1]
se_16_spp <- summary(mod_5_Biomass)$coefficients[1, 2]
lower_16_spp <- confint(mod_5_Biomass)[4, 1]
upper_16_spp <- confint(mod_5_Biomass)[4, 2]

mean_difference <- summary(mod_5_Biomass)$coefficients[7,
    1]
se_difference <- summary(mod_5_Biomass)$coefficients[7,
    2]
lower_difference <- confint(mod_5_Biomass)[10, 1]
upper_difference <- confint(mod_5_Biomass)[10, 2]


table_s8_data <- Rapideye_data %>%
    mutate(Climber_cutting = factor(Climber_cutting, levels = c("Yes",
        "No"))) %>%
    mutate(Spp_richness = factor(Spp_richness, levels = c("16",
        "4", "0", "1")))
mod_5_Biomass <- lmer(Biomass ~ factor(Spp_richness) +
    Gen_div + Canopy_type + Treatment + (1 | Block) + (1 |
    Spp_comp), data = table_s8_data)
mean_16_cut <- summary(mod_5_Biomass)$coefficients[1, 1]
se_16_cut <- summary(mod_5_Biomass)$coefficients[1, 2]
lower_16_cut <- confint(mod_5_Biomass)[4, 1]
upper_16_cut <- confint(mod_5_Biomass)[4, 2]

table_s8_Biomass <- tibble(Liana_removal = c("No", "Yes",
    "Difference"), Biomass_estimate = c(mean_16_spp, mean_16_cut,
    mean_difference), Biomass_SE = c(se_16_spp, se_16_cut,
    se_difference), Biomass_lower = c(lower_16_spp, lower_16_cut,
    lower_difference), Biomass_upper = c(upper_16_spp,
    upper_16_cut, upper_difference))


table_s8_data <- Rapideye_data %>%
    mutate(Climber_cutting = factor(Climber_cutting, levels = c("No",
        "Yes"))) %>%
    mutate(Spp_richness = factor(Spp_richness, levels = c("16",
        "4", "0", "1")))
mod_5_LAI <- lmer(LAI ~ factor(Spp_richness) + Gen_div +
    Canopy_type + Climber_cutting + (1 | Block) + (1 |
    Spp_comp), data = table_s8_data)
mean_16_spp <- summary(mod_5_LAI)$coefficients[1, 1]
se_16_spp <- summary(mod_5_LAI)$coefficients[1, 2]
lower_16_spp <- confint(mod_5_LAI)[4, 1]
```

```
upper_16_spp <- confint(mod_5_LAI)[4, 2]

mean_difference <- summary(mod_5_LAI)$coefficients[7, 1]
se_difference <- summary(mod_5_LAI)$coefficients[7, 2]
lower_difference <- confint(mod_5_LAI)[10, 1]
upper_difference <- confint(mod_5_LAI)[10, 2]


table_s8_data <- Rapideye_data %>%
    mutate(Climber_cutting = factor(Climber_cutting, levels = c("Yes",
        "No"))) %>%
    mutate(Spp_richness = factor(Spp_richness, levels = c("16",
        "4", "0", "1")))
mod_5_LAI <- lmer(LAI ~ factor(Spp_richness) + Gen_div +
    Canopy_type + Treatment + (1 | Block) + (1 | Spp_comp),
    data = table_s8_data)
mean_16_cut <- summary(mod_5_LAI)$coefficients[1, 1]
se_16_cut <- summary(mod_5_LAI)$coefficients[1, 2]
lower_16_cut <- confint(mod_5_LAI)[4, 1]
upper_16_cut <- confint(mod_5_LAI)[4, 2]

table_s8_LAI <- tibble(Liana_removal = c("No", "Yes", "Difference"),
    LAI_estimate = c(mean_16_spp, mean_16_cut, mean_difference),
    LAI_SE = c(se_16_spp, se_16_cut, se_difference), LAI_lower = c(lower_16_spp,
        lower_16_cut, lower_difference), LAI_upper = c(upper_16_spp,
        upper_16_cut, upper_difference))


table_s8_data <- Rapideye_data %>%
    mutate(Climber_cutting = factor(Climber_cutting, levels = c("No",
        "Yes"))) %>%
    mutate(Spp_richness = factor(Spp_richness, levels = c("16",
        "4", "0", "1")))
mod_5_Cover <- lmer(Cover ~ factor(Spp_richness) + Gen_div +
    Canopy_type + Climber_cutting + (1 | Block) + (1 |
    Spp_comp), data = table_s8_data)
mean_16_spp <- summary(mod_5_Cover)$coefficients[1, 1]
se_16_spp <- summary(mod_5_Cover)$coefficients[1, 2]
lower_16_spp <- confint(mod_5_Cover)[4, 1]
upper_16_spp <- confint(mod_5_Cover)[4, 2]

mean_difference <- summary(mod_5_Cover)$coefficients[7,
    1]
se_difference <- summary(mod_5_Cover)$coefficients[7, 2]
lower_difference <- confint(mod_5_Cover)[10, 1]
upper_difference <- confint(mod_5_Cover)[10, 2]


table_s8_data <- Rapideye_data %>%
    mutate(Climber_cutting = factor(Climber_cutting, levels = c("Yes",
        "No"))) %>%
    mutate(Spp_richness = factor(Spp_richness, levels = c("16",
        "4", "0", "1")))
```

```
mod_5_Cover <- lmer(Cover ~ factor(Spp_richness) + Gen_div +
    Canopy_type + Treatment + (1 | Block) + (1 | Spp_comp),
    data = table_s8_data)
mean_16_cut <- summary(mod_5_Cover)$coefficients[1, 1]
se_16_cut <- summary(mod_5_Cover)$coefficients[1, 2]
lower_16_cut <- confint(mod_5_Cover)[4, 1]
upper_16_cut <- confint(mod_5_Cover)[4, 2]

table_s8_Cover <- tibble(Liana_removal = c("No", "Yes",
    "Difference"), Cover_estimate = c(mean_16_spp, mean_16_cut,
    mean_difference), Cover_SE = c(se_16_spp, se_16_cut,
    se_difference), Cover_lower = c(lower_16_spp, lower_16_cut,
    lower_difference), Cover_upper = c(upper_16_spp, upper_16_cut,
    upper_difference))


table_s8 <- table_s8_Biomass %>%
    merge(table_s8_LAI, by = "Liana_removal") %>%
    merge(table_s8_Cover, by = "Liana_removal")
```

9.2 Graphing

```
fig_1g_data <- table_s8 %>%
    select(1, 2, 4, 5) %>%
    filter(Liana_removal != "Difference")

fig_1g <- fig_1g_data %>%
    ggplot(aes(x = Liana_removal, y = Biomass_estimate)) +
    geom_pointrange(aes(ymin = Biomass_lower, ymax = Biomass_upper,
        colour = Liana_removal, shape = Liana_removal),
        size = 1, fill = "white", shape = 22) + scale_colour_manual(values = c(cols[1],
    cols[2])) + theme(legend.position = "none") + labs(y = expression(atop("Aboveground biomass",
    paste("(Mg  ", Ha^-1, ")"))), x = "Liana removal")


fig_1g
```

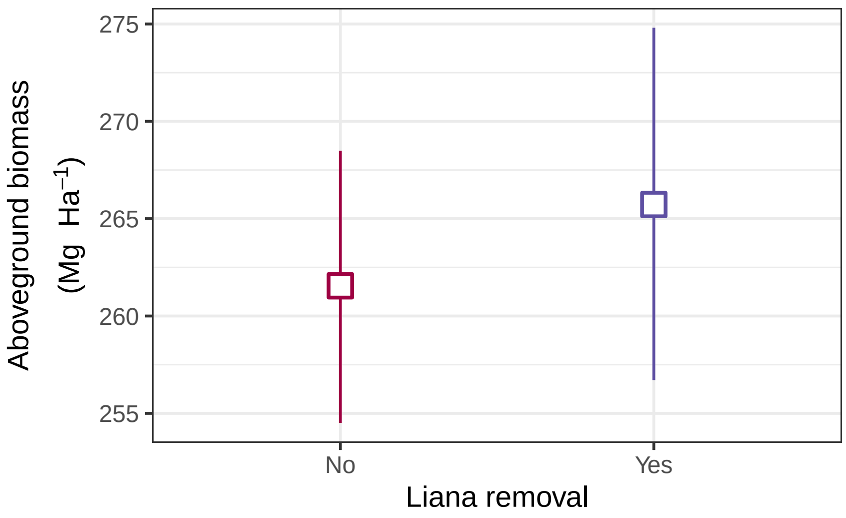


```
fig_s4_a_data <- table_s8 %>%
    select(c(1, contains("LAI"))) %>%
    filter(Liana_removal != "Difference")

fig_s4a <- fig_s4_a_data %>%
    ggplot(aes(x = Liana_removal, y = LAI_estimate)) +
    geom_pointrange(aes(ymin = LAI_lower, ymax = LAI_upper,
        colour = Liana_removal, shape = Liana_removal),
        size = 1, fill = "white", shape = 22) + scale_colour_manual(values = c(cols[1],
    cols[2])) + theme(legend.position = "none") + labs(y = "Leaf Area Index",
    x = "")

fig_s4a
```

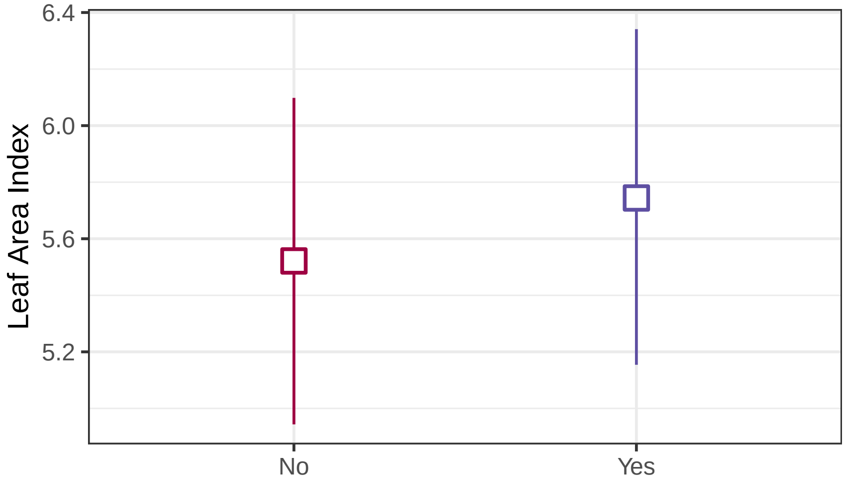


```
fig_s4_d_data <- table_s8 %>%
    select(c(1, 10, 12, 13)) %>%
    filter(Liana_removal != "Difference")
```

```
fig_s4d <- fig_s4_d_data %>%
    ggplot(aes(x = Liana_removal, y = Cover_estimate)) +
    geom_pointrange(aes(ymin = Cover_lower, ymax = Cover_upper,
        colour = Liana_removal, shape = Liana_removal),
        size = 1, fill = "white", shape = 22) + scale_colour_manual(values = c(cols[1],
    cols[2])) + theme(legend.position = "none") + labs(y = "Vegetation cover (%)",
    x = "Liana removal")

fig_s4d
```

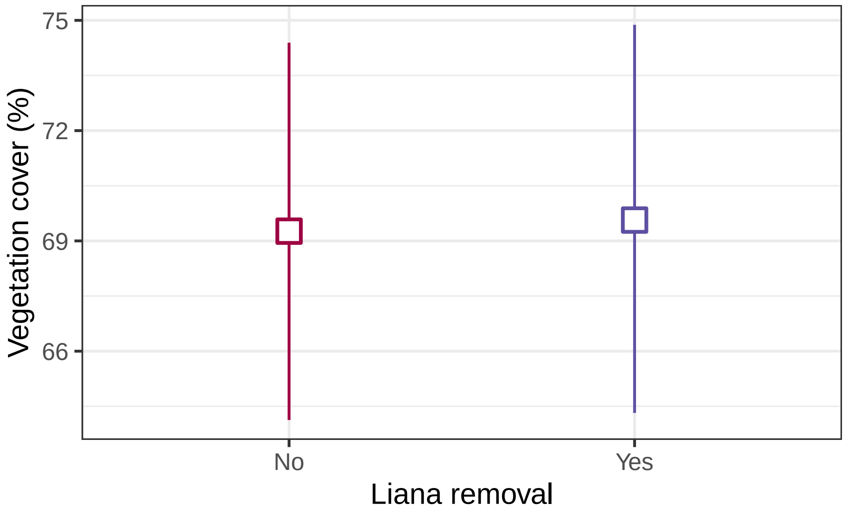

Supplement: Supplementary file 2 — Other file [file sciadv.adf0938_other_file.zip › adf0938_Suppl. Other File Type_seq1_v3.pdf]
