# Supplementary figures and images for: Positive effects of tree diversity on tropical forest restoration in a field-scale experiment
Source: Sci Adv. 2023 Sep 15;9(37):eadf0938. doi: 10.1126/sciadv.adf0938 (PMC10846868; doi:10.1126/sciadv.adf0938)

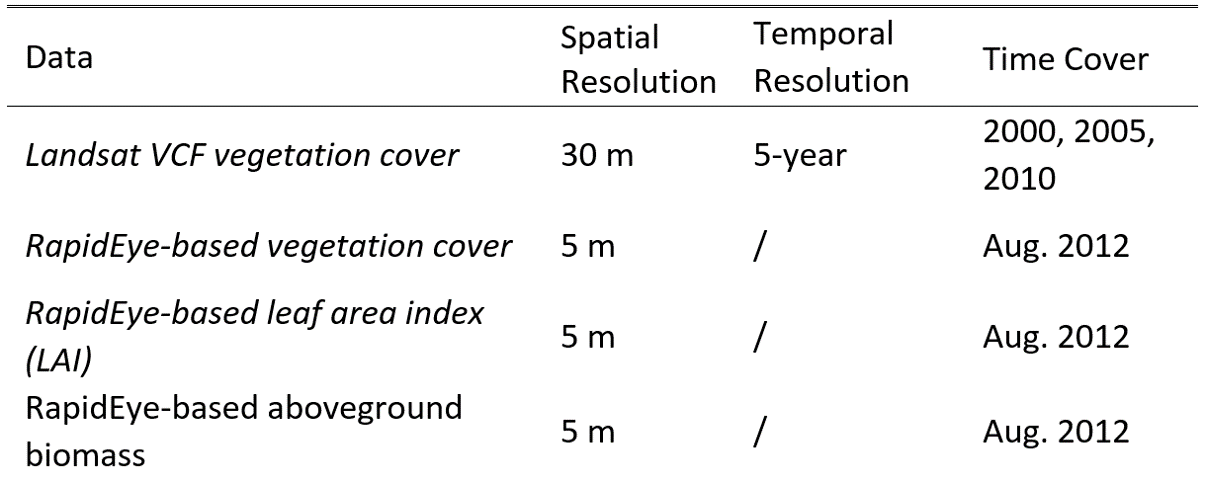

Supplement: Supplementary file 2 — Other file [file sciadv.adf0938_other_file.zip › Rmarkdown and data for paper/Data/table1.png]
